# Supplementary material for: Experimentally validated design principles of heteroatom-doped-graphene-supported calcium single-atom materials for non-dissociative chemisorption solid-state hydrogen storage
Source: Nat Commun. 2024 Jan 31;15:928. doi: 10.1038/s41467-024-45082-9 (PMC10830568; doi:10.1038/s41467-024-45082-9)
Supplement: Supplementary file 1 — Supplementary Information [file 41467_2024_45082_MOESM1_ESM.pdf]

Supplementary Information

Experimentally validated design principles of heteroatom-doped-graphene-supported calcium single-atom materials for non-dissociative chemisorption solid-state hydrogen storage

Yong Gao[1], Zhenglong Li[1], Pan Wang[2], Wen-gang Cui[1], Xiaowei Wang[3], Yaxiong Yang[1], Fan Gao[1], Mingchang Zhang[1], Jiantuo Gan[1], Chenchen Li[1], Yanxia Liu[1], Xinqiang Wang[1], Fulai Qi[1], Jing Zhang[2], Xiao Han[2], Wubin Du[4], Jian Chen[5*], Zhenhai Xia[6*] and Hongge Pan[1*]

[1]Institute of Science and Technology for New Energy Xi'an Technological University, Xi'an 710021, China

[2]School of Materials Science and Engineering, Northwestern Polytechnical University, Xi'an 710072, China,

[3]Department of Materials Science and Engineering, University of North Texas, Denton, TX 76203, USA

[4]School of Materials Science and Engineering, Zhejiang University, Hangzhou, 310058, P. R. China

[5]School of Materials Science and Chemical Engineering, Xi'an Technological University, Xi'an 710021, China

[6]Australian Carbon Materials Centre, School of Chemical Engineering, University of New South Wales, Sydney NSW, 2052 Australia

Corresponding author: E-mail: zhenhai.xia@unsw.edu.au ; E-mail: zhenhai.xia@unsw.edu.au ;
E-mail: Honggepan@zju.edu.cn

1. Supplementary Calculation Methods

1.1. Binding energy calculation of Ca atom anchored on X-G supports

During the formation of X-G-Ca single-atom H_2 storage materials, Ca atoms will be chemisorbed on surfaces of the graphene doped with heteroatoms X (X-G). Thus, the binding energy E_b between the Ca atom and X-G support is given by

$$E_b = E(X - G - Ca) - E(X - G) - E(Ca) \quad (1)$$

where $E(X - G - Ca)$ and $E(X - G)$ are the total energy of substrates with/without Ca chemisorption at ground state, respectively and can be obtained via DFT calculation, and $E(Ca)$ is the energy of single Ca atom with the value of -1.9863 eV calculated from DFT in VASP.

1.2. Adsorption energy and Gibbs free energy change for H_2 molecule on surface of X-G-Ca H_2 storage materials

In the process of H_2 storage, H_2 molecules are adsorbed on H_2 storage sites of X-G-Ca surface, while upon releasing, H_2 molecules adsorbed on sites of X-G-Ca surface will desorb reversibly. Therefore, the process can be written as

$$\mathrm{H_2} + * \underset{\boldsymbol{Desorbing}}{\overset{\boldsymbol{Adsorbing}}{\rightleftharpoons}} \mathrm{H_2}^* \quad (2)$$

where * refers to H_2 adsorption site on X-G-Ca surface, and $\mathrm{H_2}$ and $\mathrm{H_2}^*$ represent the free H_2 molecules in gas and H_2 adsorbed to sites, respectively. The energy change of H_2 molecule adsorption $\Delta E_{H_2^*}$ is expressed as

$$\Delta E_{H_2^*} = E(X - G - Ca - H_2) - E(X - G - Ca) - E(H_2) \quad (3)$$

where $E(X - G - Ca - H_2)$ is the total energy of X-G-Ca substrate adsorbing H_2 molecule, $E(X - G - Ca)$ is the total energy of X-G-Ca substrate without any H_2 molecules adsorption, and $E(H_2)$ is the energy of a H_2 molecule with the value of -6.764eV. Also, based on the above expression, the averaged adsorption energy change $\Delta \bar{E}_{H_2^*}$ upon n H_2 molecules adsorbed on X-G-Ca surface, is expressed as

$$\Delta \bar{E}_{H_2^*} = [E(X - G - Ca - H_2) - E(X - G - Ca) - nE(H_2)]/n \quad (4)$$

In addition, at T=298K and P=1bar, the Gibbs free energy change of H_2 adsorption in Equation (S2) can be expressed by

$$\Delta G_{H_2^*} = \Delta E_{H_2^*} + \Delta E_{ZPE} - T\Delta S_{H_2^*} \quad (5)$$

where $\Delta E_{H_2^*}$ is adsorption energy change as mentioned in Equation (3), ΔE_{ZPE} is zero-point energy change of H_2 molecule at adsorbed/dissociative states, and is calculated to be -0.07eV by DFT methods, $-T\Delta S_{H_2^*} = 0.404\ eV$ is the correction coming from external condition, and *T* and *P* refer to temperature in unit of K and pressure in unit of bar, respectively.

Here, for the simplicity, at *T*=298K and *P*=1bar

$$\Delta G_{H_2^*} = \Delta E_{H_2^*} + 0.334 \quad (6a)$$

At *T*=100K and *P*=1bar,

$$\Delta G_{H_2^*} = \Delta E_{H_2^*} + 0.035 \quad (6b)$$

At *T*=0K, namely ground state,

$$\Delta G_{H_2^*} = \Delta E_{H_2^*} - 0.07 \quad (6c)$$

where $\Delta E_{ZPE} = -0.07eV$.

If $\Delta G_{H_2^*}$ is greater than or equal to zero, H_2 cannot be adsorbed spontaneously under the standard condition. To enhance the H_2 adsorption ability, it needs to change the external driving conditions and/or modify the material structures, which leads to additional free energy change, $\Delta G(T, P, E, B)$. Thus, the total energy change under the conditions can be written as

$$\Delta G = \Delta G_{H_2^*} + \Delta G(T, P, E, B) \quad (7)$$

Here, temperature *T*, pressure *P*, electric field *E* and magnetic field *B* represent external factors that drive ΔG to be less than zero for spontaneous hydrogen adsorption.

Under any temperature *T* and pressure *P*, Equation (5) can be rewritten as $\Delta G_{H_2^*}(T, P) = \Delta E_{H_2^*} + \Delta E_{ZPE} + \Delta G(T, P)$. Here, $\Delta G(T, P)$ is the correction coming from external condition, and *T* and *P* refer to temperature in unit of K and pressure in unit of bar, respectively. $\Delta G(T, P) = \Delta G(T, P_0) + k_B T ln(\frac{P_1}{P_2})$. Here, $\Delta G(T, P_0)$ is the Gibbs free energy change for any temperature *T* at standard pressure of 1bar, and represents the energy change due to varying temperature *T*, and k_B is Boltzmann constant. P_1 and P_2 refer to the initial hydrogen partial pressure and final hydrogen partial pressure.

1.3. H_2 storage capacity, rate and their volcanic trend

According to the above H_2 storage mechanism, we put forward a H_2 storage theory that correlate the H_2 storage capacity and rate with the structures of X-G-Ca materials based on the previous

proposed theoretical model of energy level filling for cations storage[1]. Under certain thermodynamic conditions for adsorbing reaction in Equation (S2) ($H_2 + * \rightarrow H_2{}^*$), we assume that H_2 molecules will adsorb on Ca ion and its surrounding regions with different H_2 adsorption energy $\Delta G_{H_2{}^*}$, and these regions with the lower H_2 adsorption energy $\Delta G_{H_2{}^*}$ are easier to adsorb H_2 molecules under the external driving conditions $\Delta G(T,P)$ (negative ΔG) according to Equation (S7).

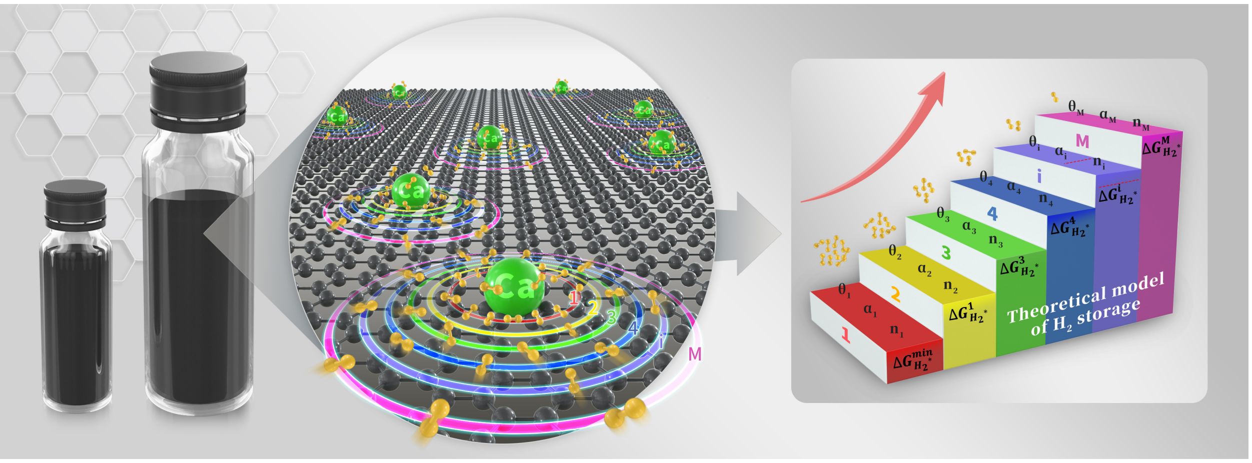

Supplementary Fig.0 The theoretical model of generalized Sabatier principle for hydrogen molecules storage proposed by us.

Thus, as shown in Fig. S0, we divided regions with various $\Delta G^{i}_{H_2{}^*}$ into different energy level i in the order of $\Delta G^{i}_{H_2{}^*}$ from low to high, on which the number of total H_2 molecules storage sites, a_i, the number of H_2 molecules really adsorbed on sites, n_i, and the corresponding coverage, θ_i, were set to build H_2 storage capacity, rate and their respective trend.

Therefore, for the whole X-G-Ca H_2 storage system with M regions corresponding to M energy levels, the total number of H_2 molecules stored, N, and the total number of sites used to adsorb H_2 molecules, A, H_2 storage capacity, $C_{H_2/site}$, and the coverage, θ_i, of H_2 molecules on each energy level, i, can be expressed easily as

$$N = n_1 + n_2 + n_3 + \cdots + n_M \tag{8}$$

$$A = a_1 + a_2 + a_3 + \cdots + a_M \tag{9}$$

$$C_{H_2/site} = \frac{N}{A} \tag{10}$$

$$\theta_i = \frac{n_i}{a_i} \tag{11}$$

In addition, in the consideration of H_2 molecules adsorption rules in accordance with Boltzmann distribution characteristics, the amount of H_2 molecules on each energy level was expressed as

$$n_i = \lambda e^{-\frac{\Delta G^i_{H_2{}^*}}{k_B T}} \tag{12}$$

where λ is pre-factor of Boltzmann distribution rule, and k_B and T are Boltzmann constant and temperature, respectively.

Additionally, in the light of the small increment of H_2 adsorption energy per adsorbing a H_2 molecule from DFT theoretical calculation, the adsorption energy change at each energy level can be expressed approximately as

$$\Delta G^i_{H_2{}^*} = \Delta G^{min}_{H_2{}^*} + \frac{\Delta G^{max}_{H_2{}^*} - \Delta G^{min}_{H_2{}^*}}{M} i \tag{13}$$

Therefore, according to Equations (8), (12) and (13), the total number of H_2 molecules adsorbed on X-G-Ca H_2 storage system can be given by

$$\begin{aligned} N &= n_1 + n_2 + n_3 + \cdots + n_M \\ &= \lambda e^{-\frac{\Delta G^1_{H_2{}^*}}{k_B T}} + \lambda e^{-\frac{\Delta G^2_{H_2{}^*}}{k_B T}} + \lambda e^{-\frac{\Delta G^3_{H_2{}^*}}{k_B T}} + \cdots + \lambda e^{-\frac{\Delta G^M_{H_2{}^*}}{k_B T}} \\ &= \sum_{i=1}^{M} \lambda e^{-\frac{\Delta G^i_{H_2{}^*}}{k_B T}} \\ &= \frac{\lambda M}{\Delta G^{max}_{H_2{}^*} - \Delta G^{min}_{H_2{}^*}} \times \frac{\Delta G^{max}_{H_2{}^*} - \Delta G^{min}_{H_2{}^*}}{M} \sum_{i=1}^{M} e^{-\frac{\Delta G^i_{H_2{}^*}}{k_B T}} \\ &= \frac{\lambda M}{\Delta G^{max}_{H_2{}^*} - \Delta G^{min}_{H_2{}^*}} \int_{\Delta G^{min}_{H_2{}^*}}^{\Delta G^{max}_{H_2{}^*}} e^{-\frac{x}{k_B T}} dx \end{aligned}$$

Namely,

$$N = \frac{\lambda M}{\Delta G^{max}_{H_2{}^*} - \Delta G^{min}_{H_2{}^*}} \int_{\Delta G^{min}_{H_2{}^*}}^{\Delta G^{max}_{H_2{}^*}} e^{-\frac{x}{k_B T}} dx \tag{14}$$

On the other hand, the H_2 molecules coverage θ_i on each energy level i can be related to equilibrium constant K_i by

$$\theta_i = \frac{K_i}{1+K_i} \tag{15}$$

Also, equilibrium constant K_i is able to be related to the change of Gibbs free energy $\Delta G^i_{H_2{}^*}$ before and after H_2 adsorption reaction (S2) by

$$K_i = e^{-\frac{\Delta G^i_{H_2{}^*}}{k_B T}} \tag{16}$$

Thus, combining (15) and (16), the H_2 molecules coverage θ_i on each energy level i was finally given by

$$\theta_i = \frac{1}{1+e^{\frac{\Delta G^{i}_{H_2^*}}{k_B T}}} \tag{17}$$

Hence, combining Equations (11), (12) and (17), the number of H_2 storage sites a_i on each energy level *i* was given by

$$a_i = \lambda\left(1 + e^{-\frac{\Delta G^{i}_{H_2^*}}{k_B T}}\right) \tag{18}$$

Consequently, the total number of H_2 adsorption sites on practical X-G-Ca H_2 storage system surface is able to be given by

$$\begin{aligned} A &= a_1 + a_2 + a_3 + \cdots + a_M \\ &= \lambda\left(1 + e^{-\frac{\Delta G^{1}_{H_2^*}}{k_B T}}\right) + \lambda\left(1 + e^{-\frac{\Delta G^{2}_{H_2^*}}{k_B T}}\right) + \cdots + \lambda\left(1 + e^{-\frac{\Delta G^{M}_{H_2^*}}{k_B T}}\right) \\ &= \frac{\lambda M}{\Delta G^{max}_{H_2^*} - \Delta G^{min}_{H_2^*}} \times \frac{G^{max}_{H_2^*} - \Delta G^{min}_{H_2^*}}{M} \sum_{i=1}^{M}\left(1 + e^{-\frac{\Delta G^{i}_{H_2^*}}{k_B T}}\right) \\ &= \frac{\lambda M}{\Delta G^{max}_{H_2^*} - \Delta G^{min}_{H_2^*}} \int_{\Delta G^{min}_{H_2^*}}^{\Delta G^{max}_{H_2^*}} \left(1 + e^{-\frac{x}{k_B T}}\right) dx \end{aligned} \tag{19}$$

Namely, positive A can be expressed as

$$A = \frac{\lambda M}{\Delta G^{max}_{H_2^*} - \Delta G^{min}_{H_2^*}} \int_{\Delta G^{min}_{H_2^*}}^{\Delta G^{max}_{H_2^*}} \left(1 + e^{-\frac{x}{k_B T}}\right) dx \tag{20}$$

or

$$A = \frac{\lambda M}{\Delta G^{max}_{H_2^*} - \Delta G^{min}_{H_2^*}} \left[\left(\Delta G^{max}_{H_2^*} - \Delta G^{min}_{H_2^*}\right) - k_B T\left(e^{-\frac{\Delta G^{max}_{H_2^*}}{k_B T}} - e^{-\frac{\Delta G^{min}_{H_2^*}}{k_B T}}\right)\right] \tag{21}$$

Therefore, combining Equations (10), (14) and (20), hydrogen storage capacity, namely the mean number of H_2 molecules per unit site, can finally be derived as

$$C_{H_2/site} = \frac{N}{A} = \frac{\frac{\lambda M}{\Delta G^{max}_{H_2^*} - \Delta G^{min}_{H_2^*}} \int_{\Delta G^{min}_{H_2^*}}^{\Delta G^{max}_{H_2^*}} e^{-\frac{x}{k_B T}} dx}{\frac{\lambda M}{\Delta G^{max}_{H_2^*} - \Delta G^{min}_{H_2^*}} \int_{\Delta G^{min}_{H_2^*}}^{\Delta G^{max}_{H_2^*}} \left(1+e^{-\frac{x}{k_B T}}\right) dx} \tag{22}$$

1) For the case of $\Delta G^{max}_{H_2^*} < 0$,

$$C_{H_2/site} = \frac{N}{A} = 0 \tag{23}$$

Its physical meaning is that H_2 cannot desorb reversibly under ambient temperature and pressure (T =298K and P=1bar) from the perspective of thermodynamic. Only when additional external conditions such as higher temperature or lower pressure are provided, can hydrogen gas desorb.

2) For the case of $\Delta G_{H_2^*}^{min} > 0$,

$$C_{H_2/site} = \frac{N}{A} = \frac{\int_{\Delta G_{H_2^*}^{min}}^{\Delta G_{H_2^*}^{max}} e^{-\frac{x}{k_BT}} dx}{\int_{\Delta G_{H_2^*}^{min}}^{\Delta G_{H_2^*}^{max}} \left(1+e^{-\frac{x}{k_BT}}\right) dx} = \frac{-k_BT\left(e^{-\frac{\Delta G_{H_2^*}^{max}}{k_BT}} - e^{-\frac{\Delta G_{H_2^*}^{min}}{k_BT}}\right)}{\left(\Delta G_{H_2^*}^{\text{[illegible]}ax} - \Delta G_{H_2^*}^{min}\right) - k_BT\left(e^{-\frac{\Delta G_{H_2^*}^{max}}{k_BT}} - e^{-\frac{\Delta G_{H_2^*}^{min}}{k_BT}}\right)} \quad (24)$$

Its physical meaning is that H_2 storage is a reversible process in which H_2 is stored upon application of external driving condition $\Delta G(T,P,E,B)$, whereas H_2 is desorbed when external driving condition $\Delta G(T,P,E,B)$ is removed.

3) For the case of $\Delta G_{H_2^*}^{min} < 0$ and $\Delta G_{H_2^*}^{max} > 0$,

$$N = \frac{\lambda M}{\Delta G_{H_2^*}^{max} - \Delta G_{H_2^*}^{min}} \int_{\Delta G_{H_2^*}^{min}}^{\Delta G_{H_2^*}^{max}} e^{-\frac{x}{k_BT}} dx$$

$$= \frac{\lambda M}{\Delta G_{H_2^*}^{max} - \Delta G_{H_2^*}^{min}} \int_{\Delta G_{H_2^*}^{min}}^{0} e^{-\frac{x}{k_BT}} dx + \frac{\lambda M}{\Delta G_{H_2^*}^{max} - \Delta G_{H_2^*}^{min}} \int_{0}^{\Delta G_{H_2^*}^{max}} e^{-\frac{x}{k_BT}} dx$$

$$= 0 + \frac{\lambda M}{\Delta G_{H_2^*}^{max} - \Delta G_{H_2^*}^{min}} \int_{0}^{\Delta G_{H_2^*}^{max}} e^{-\frac{x}{k_BT}} dx$$

$$= \frac{\lambda M k_BT\left(1-e^{-\frac{\Delta G_{H_2^*}^{max}}{k_BT}}\right)}{\Delta G_{H_2^*}^{max} - \Delta G_{H_2^*}^{min}}$$

Thus,

$$C_{H_2/site} = \frac{N}{A} = \frac{k_BT\left(1-e^{-\frac{\Delta G_{H_2^*}^{max}}{k_BT}}\right)}{\left(\Delta G_{H_2^*}^{max} - \Delta G_{H_2^*}^{min}\right) - k_BT\left(e^{-\frac{\Delta G_{H_2^*}^{max}}{k_BT}} - e^{-\frac{\Delta G_{H_2^*}^{min}}{k_BT}}\right)} \quad (25)$$

Conclusively, for the case of $\Delta G_{H_2^*}^{max} > 0$, the reversible H_2 storage capacity $C_{H_2/site}$ can be expressed as

$$C_{H_2/site} = \begin{cases} \dfrac{-k_BT\left(e^{-\frac{\Delta G^{max}_{H_2^*}}{k_BT}}-e^{-\frac{\Delta G^{min}_{H_2^*}}{k_BT}}\right)}{\left(\Delta G^{max}_{H_2^*}-\Delta G^{min}_{H_2^*}\right)-k_BT\left(e^{-\frac{\Delta G^{max}_{H_2^*}}{k_BT}}-e^{-\frac{\Delta G^{min}_{H_2^*}}{k_BT}}\right)}, & \Delta G^{min}_{H_2^*} > 0 \\ \dfrac{k_BT\left(1-e^{-\frac{\Delta G^{max}_{H_2^*}}{k_BT}}\right)}{\left(\Delta G^{max}_{H_2^*}-\Delta G^{min}_{H_2^*}\right)-k_BT\left(e^{-\frac{\Delta G^{max}_{H_2^*}}{k_BT}}-e^{-\frac{\Delta G^{min}_{H_2^*}}{k_BT}}\right)}, & \Delta G^{min}_{H_2^*} < 0 \end{cases} \tag{26}$$

Next, we focus on the express for H_2 storage rate. According to Equation (2), it is obvious that adsorption rate (or reaction rate), namely forward rate r in Equation (2) is equal to one H_2 molecule storage rate on any unit site that can be explained as the one H_2 molecule adsorption speed. Thus, on the basis of Langmuir adsorption isothermal assumption that adsorption rate of adsorbate is proportional to the fraction of uncovered region of substrate surface (1- θ), where θ is coverage of adsorbate, any one H_2 molecule adsorption rate, r_i, can be given as

$$r_i = k_i(1-\theta_i)C_{H_2} \tag{27}$$

where k_i and C_{H_2} are rate constant and molar concentration of H_2 molecules, respectively.

Additionally, based on microkinetic model in which Arrhenius equation describing reaction rate was modified approximately, the rate constant k_i in Equations (27) for particular H_2 molecule adsorption process in X-G-Ca system can be rewritten as

$$k_i = k_0 e^{-\frac{\Delta G^{i}_{H_2^*}}{k_BT}} \tag{28}$$

Thus, any one H_2 molecule adsorption rate r_i can be rewritten as

$$r_i = \frac{k_0 C_{H_2}}{1+e^{\frac{\Delta G^{i}_{H_2^*}}{k_BT}}} \tag{29}$$

Consequently, according to $t_i=\frac{1}{r_i}$, the time t_i spent for adsorbing any one H_2 molecules can be given by

$$t_i = \frac{1+e^{\frac{\Delta G^{i}_{H_2^*}}{k_BT}}}{k_0 C_{H_2}} \tag{30}$$

Moreover, assuming that hydrogen molecules with the same Gibbs free energy change $\Delta G^{i}_{H_2^*}$ (or on the same energy level i) are simultaneously adsorbed on suface of X-G-Ca in the same time t_i,

thus, based on $t = t_1 + t_2 + t_3 + \cdots + t_M$ and Equation (30), the total time t spent for storing N hydrogen molecules can be expressed as

$$t = \frac{M}{\Delta G_{H_2^*}^{max} - \Delta G_{H_2^*}^{min}} \int_{\Delta G_{H_2^*}^{min}}^{\Delta G_{H_2^*}^{max}} \frac{1}{k_0 c_{H_2}} \left(1 + e^{\frac{x}{k_B T}}\right) dx \tag{31}$$

1) For the case of $\Delta G_{H_2^*}^{max} < 0$,

$$t = 0$$

Its physical meaning is that this is a spontaneous H_2 storage process that does not involve time.

2) For the case of $\Delta G_{H_2^*}^{min} > 0$,

$$t = \frac{M}{\Delta G_{H_2^*}^{max} - \Delta G_{H_2^*}^{min}} \int_{\Delta G_{H_2^*}^{min}}^{\Delta G_{H_2^*}^{max}} \frac{1}{k_0 c_{H_2}} \left(1 + e^{\frac{x}{k_B T}}\right) dx = \frac{M\left[\left(\Delta G_{H_2^*}^{max} - \Delta G_{H_2^*}^{min}\right) + k_B T\left(e^{\frac{\Delta G_{H_2^*}^{max}}{k_B T}} - e^{\frac{\Delta G_{H_2^*}^{min}}{k_B T}}\right)\right]}{k_0 c_{H_2}\left(\Delta G_{H_2^*}^{max} - \Delta G_{H_2^*}^{min}\right)} \tag{32}$$

3) For the case of $\Delta G_{H_2^*}^{min} < 0$ and $\Delta G_{H_2^*}^{max} > 0$,

$$t = \frac{M}{\Delta G_{H_2^*}^{max} - \Delta G_{H_2^*}^{min}} \int_{\Delta G_{H_2^*}^{min}}^{\Delta G_{H_2^*}^{max}} \frac{1}{k_0 c_{H_2}} \left(1 + e^{\frac{x}{k_B T}}\right) dx$$

$$= \frac{M}{\Delta G_{H_2^*}^{max} - \Delta G_{H_2^*}^{min}} \int_{\Delta G_{H_2^*}^{min}}^{0} \frac{1}{k_0 c_{H_2}} \left(1 + e^{\frac{x}{k_B T}}\right) dx + \frac{M}{\Delta G_{H_2^*}^{max} - \Delta G_{H_2^*}^{min}} \int_{0}^{\Delta G_{H_2^*}^{max}} \frac{1}{k_0 c_{H_2}} \left(1 + e^{\frac{x}{k_B T}}\right) dx$$

$$= 0 + \frac{M}{\Delta G_{H_2^*}^{max} - \Delta G_{H_2^*}^{min}} \int_{0}^{\Delta G_{H_2^*}^{max}} \frac{1}{k_0 c_{H_2}} \left(1 + e^{\frac{x}{k_B T}}\right) dx$$

$$= \frac{M\left[\Delta G_{H_2^*}^{max} + k_B T\left(e^{\frac{\Delta G_{H_2^*}^{max}}{k_B T}} - 1\right)\right]}{k_0 c_{H_2}\left(\Delta G_{H_2^*}^{max} - \Delta G_{H_2^*}^{min}\right)}$$

Namely,

$$t = \frac{M\left[\Delta G_{H_2^*}^{max} + k_B T\left(e^{\frac{\Delta G_{H_2^*}^{max}}{k_B T}} - 1\right)\right]}{k_0 c_{H_2}\left(\Delta G_{H_2^*}^{max} - \Delta G_{H_2^*}^{min}\right)} \tag{33}$$

Conclusively, for $\Delta G_{H_2^*}^{max} > 0$, the total time t can be given by

$$t = \begin{cases} \dfrac{M\left[\left(\Delta G_{H_2^*}^{max} - \Delta G_{H_2^*}^{min}\right) + k_B T\left(e^{\frac{\Delta G_{H_2^*}^{max}}{k_B T}} - e^{\frac{\Delta G_{H_2^*}^{min}}{k_B T}}\right)\right]}{k_0 c_{H_2}\left(\Delta G_{H_2^*}^{max} - \Delta G_{H_2^*}^{min}\right)}, & \Delta G_{H_2^*}^{min} > 0 \\ \dfrac{M\left[\Delta G_{H_2^*}^{max} + k_B T\left(e^{\frac{\Delta G_{H_2^*}^{max}}{k_B T}} - 1\right)\right]}{k_0 c_{H_2}\left(\Delta G_{H_2^*}^{max} - \Delta G_{H_2^*}^{min}\right)}, & \Delta G_{H_2^*}^{min} < 0 \end{cases} \tag{34}$$

Here,

For $\Delta G_{H_2^*}^{min} > 0$, the total sites A can be expressed as

$$A = \frac{\lambda M}{\Delta G_{H_2^*}^{max} - \Delta G_{H_2^*}^{min}}\left[\left(\Delta G_{H_2^*}^{max} - \Delta G_{H_2^*}^{min}\right) - k_B T\left(e^{-\frac{\Delta G_{H_2^*}^{max}}{k_B T}} - e^{-\frac{\Delta G_{H_2^*}^{min}}{k_B T}}\right)\right] \tag{35}$$

For $\Delta G_{H_2^*}^{min} < 0$, the total possible sites A can be given by

$$A = \frac{\lambda M}{\Delta G_{H_2^*}^{max} - \Delta G_{H_2^*}^{min}}\left[\Delta G_{H_2^*}^{max} - k_B T\left(e^{-\frac{\Delta G_{H_2^*}^{max}}{k_B T}} - 1\right)\right] \tag{36}$$

Thus, combining (34), (35) and (36), according to $\bar{t} = \frac{t}{A}$, the average time $\bar{t}$ spent for storing H_2 molecules on per a site can be derived as

$$\bar{t} = \begin{cases} \dfrac{\left(\Delta G_{H_2^*}^{max} - \Delta G_{H_2^*}^{min}\right) + k_B T\left(e^{\frac{\Delta G_{H_2^*}^{max}}{k_B T}} - e^{\frac{\Delta G_{H_2^*}^{min}}{k_B T}}\right)}{\lambda k_0 c_{H_2}\left[\left(\Delta G_{H_2^*}^{max} - \Delta G_{H_2^*}^{min}\right) - k_B T\left(e^{-\frac{\Delta G_{H_2^*}^{max}}{k_B T}} - e^{-\frac{\Delta G_{H_2^*}^{min}}{k_B T}}\right)\right]}, & \Delta G_{H_2^*}^{min} > 0 \\ \dfrac{\Delta G_{H_2^*}^{max} + k_B T\left(e^{\frac{\Delta G_{H_2^*}^{max}}{k_B T}} - 1\right)}{\lambda k_0 c_{H_2}\left[\Delta G_{H_2^*}^{max} - k_B T\left(e^{-\frac{\Delta G_{H_2^*}^{max}}{k_B T}} - 1\right)\right]}, & \Delta G_{H_2^*}^{min} < 0 \end{cases} \tag{37}$$

Finally, combining (26) and (37), hydrogen storage rate, $v_{H_2/site/s} = \frac{c_{H_2/site}}{\bar{t}}$ can further be expressed as

$$v_{H2/site/s} = \begin{cases} \dfrac{-\lambda k_0 c_{H_2} k_B T\left(e^{-\frac{\Delta G_{H_2^*}^{max}}{k_B T}} - e^{-\frac{\Delta G_{H_2^*}^{min}}{k_B T}}\right)}{\left(\Delta G_{H_2^*}^{max} - \Delta G_{H_2^*}^{min}\right) + k_B T\left(e^{\frac{\Delta G_{H_2^*}^{max}}{k_B T}} - e^{\frac{\Delta G_{H_2^*}^{min}}{k_B T}}\right)}, \ \Delta G_{H_2^*}^{i} > 0 \\ \dfrac{\lambda k_0 c_{H_2} k_B T\left(1 - e^{-\frac{\Delta G_{H_2^*}^{max}}{k_B T}}\right)\left[\Delta G_{H_2^*}^{max} - k_B T\left(e^{-\frac{\Delta G_{H_2^*}^{max}}{k_B T}} - 1\right)\right]}{\left[\left(\Delta G_{H_2^*}^{max} - \Delta G_{H_2^*}^{min}\right) - k_B T\left(e^{-\frac{\Delta G_{H_2^*}^{max}}{k_B T}} - e^{-\frac{\Delta G_{H_2^*}^{min}}{k_B T}}\right)\right]\left[\Delta G_{H_2^*}^{max} + k_B T\left(e^{\frac{\Delta G_{H_2^*}^{max}}{k_B T}} - 1\right)\right]}, \Delta G_{H_2^*}^{i} < 0 \end{cases} \quad (38)$$

It deserves to note that the rate constant k_0, Boltzmann factor λ , and H_2 concentration c_{H_2} in Equations are constants for a particular system.

2. Supplementary Figures

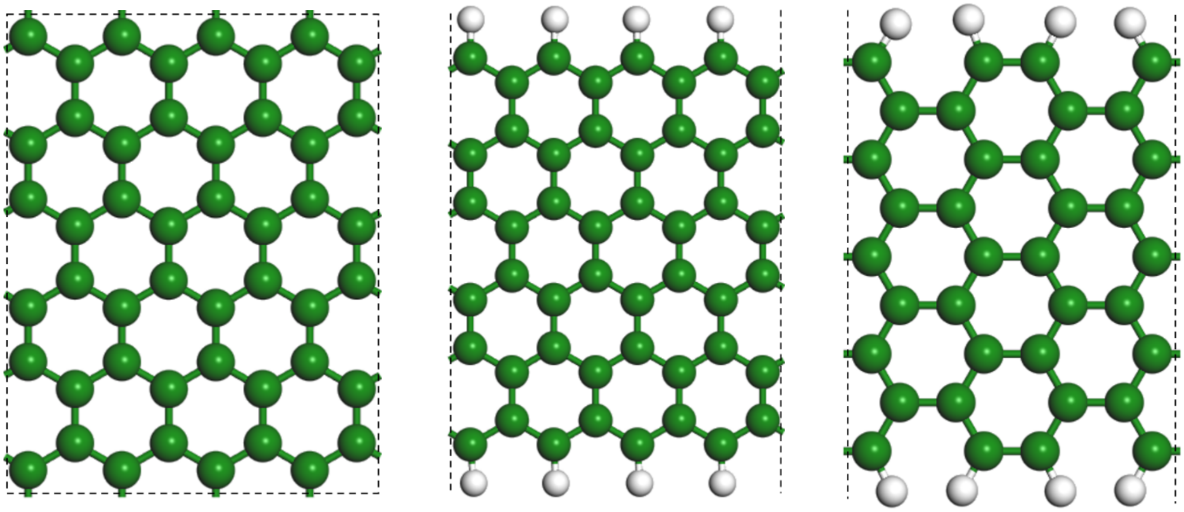

(a) (b) (c)

Supplementary Fig. 1 Summary of graphene models: (a) Graphene sheet model, (b) zigzag graphene nanoribbon and (c) armchair graphene nanoribbons.

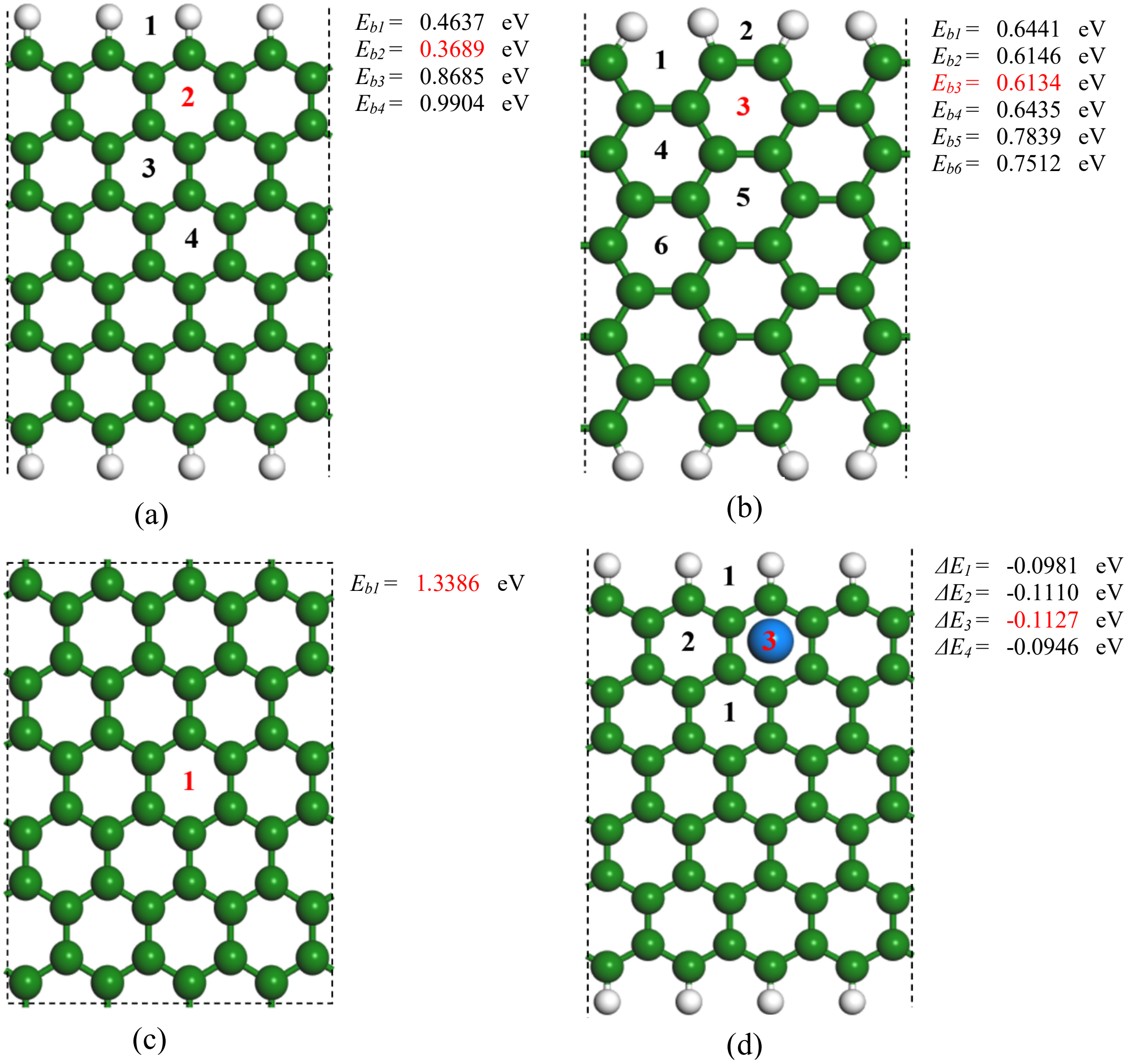

Supplementary Fig. 2 Investigated possible metal calcium atom (Ca) chemisorption sites and corresponding the binding energy upon adsorbing a calcium atom on hollow sites of (a) z-C, (b) a-C and (c) g-C graphene models. And the most stable graphene supported Ca single atom structure (z-C-Ca) (d) selected from all graphene supported Ca single models was used to check the optimal H_2 molecule adsorption sites from all possible H_2 adsorption sites and the adsorption energy change before and after H_2 molecule adsorption was calculated. We denoted possible active sites as the Arabic numbers, in which the red numbers represented the best ones with more negative binding energy (E_b) or adsorption energy(ΔE). (This marking and naming methods is the same in the following Supplementary Figs.)

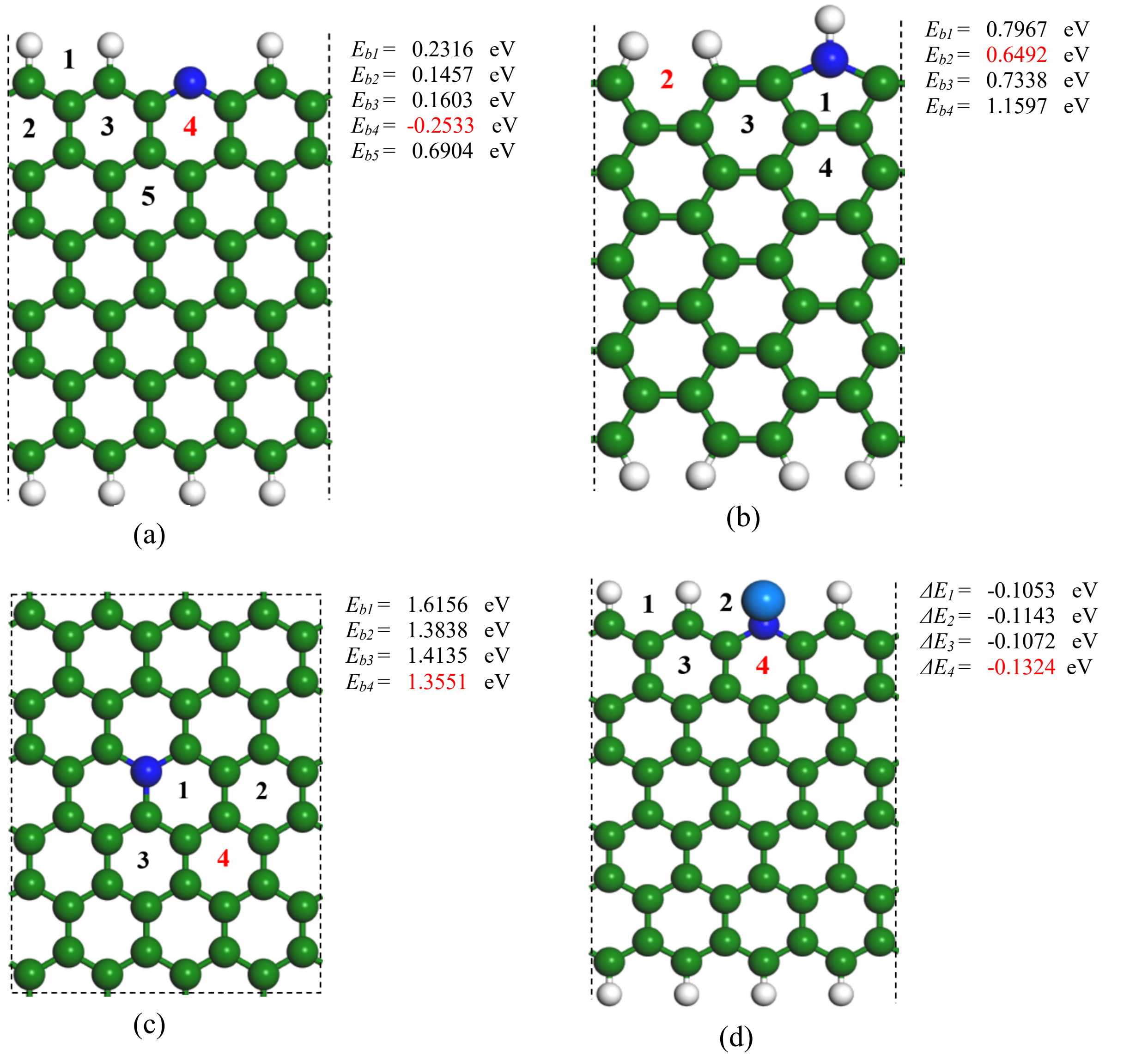

Supplementary Fig. 3 Investigated possible metal calcium atom (Ca) chemisorption sites and corresponding the binding energy upon adsorbing a calcium atom on hollow sites of (a) py-N, (b) pr-N and (c) g-N graphene models. And the most stable N-doped graphene supported Ca single-atom structure (py-N-G-Ca) (d) selected from all N-doped graphene supported Ca single models was used to check the optimal H_2 molecule adsorption sites from all possible H_2 adsorption sites and the adsorption energy change before and after H_2 molecule adsorption was calculated.

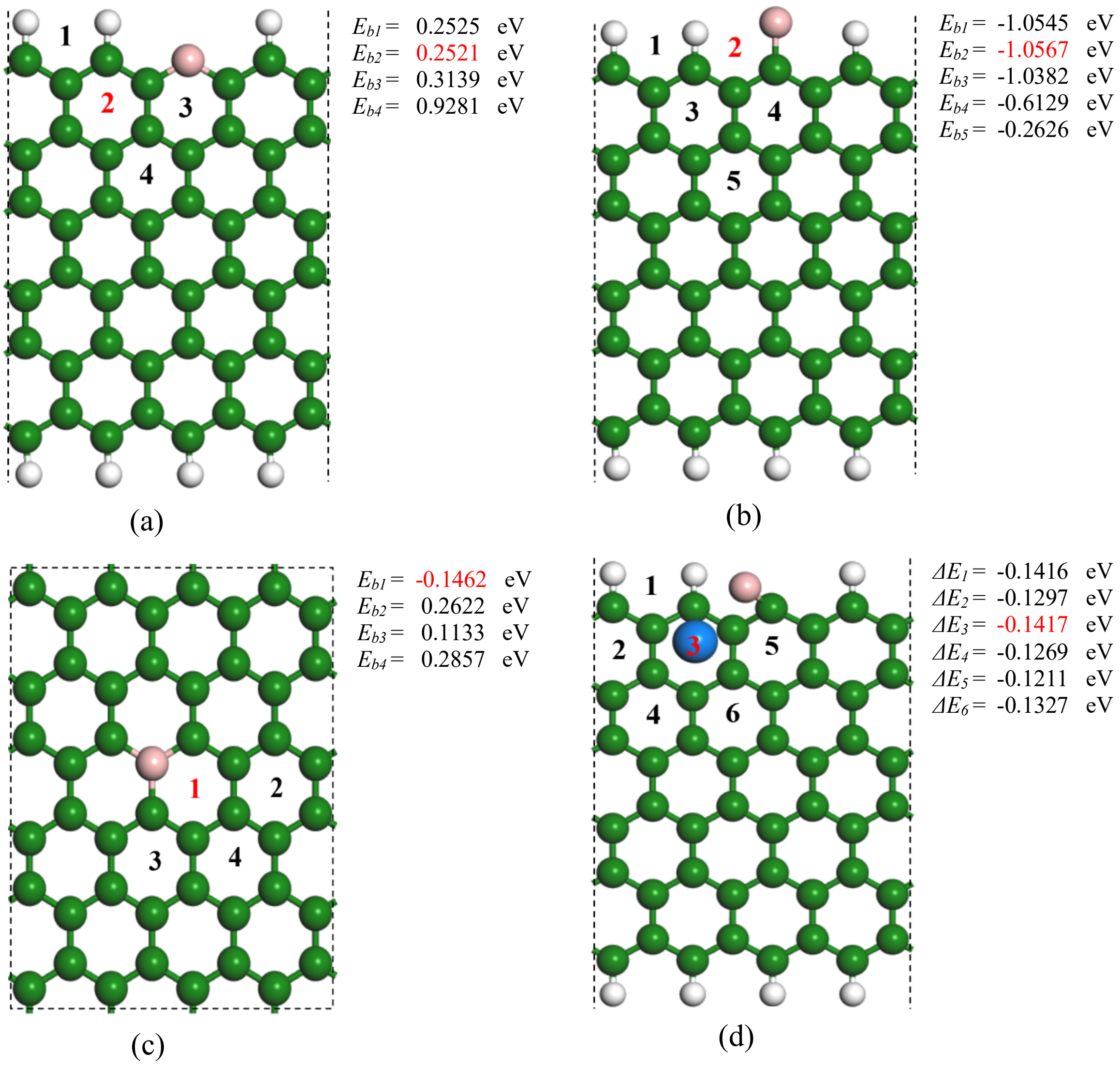

Supplementary Fig. 4 Investigated possible metal calcium atom (Ca) chemisorption sites and corresponding the binding energy upon adsorbing a calcium atom on hollow sites of (a) 2C-B, (b) B=C and (c) B-3C graphene models. And the most stable B-doped graphene supported Ca single-atom structure (B=C-G-Ca) (d) selected from all B-doped graphene supported Ca single-atom models was used to check the optimal H_2 molecule adsorption sites from all possible H_2 adsorption sites and the adsorption energy change before and after H_2 molecule adsorption was calculated.

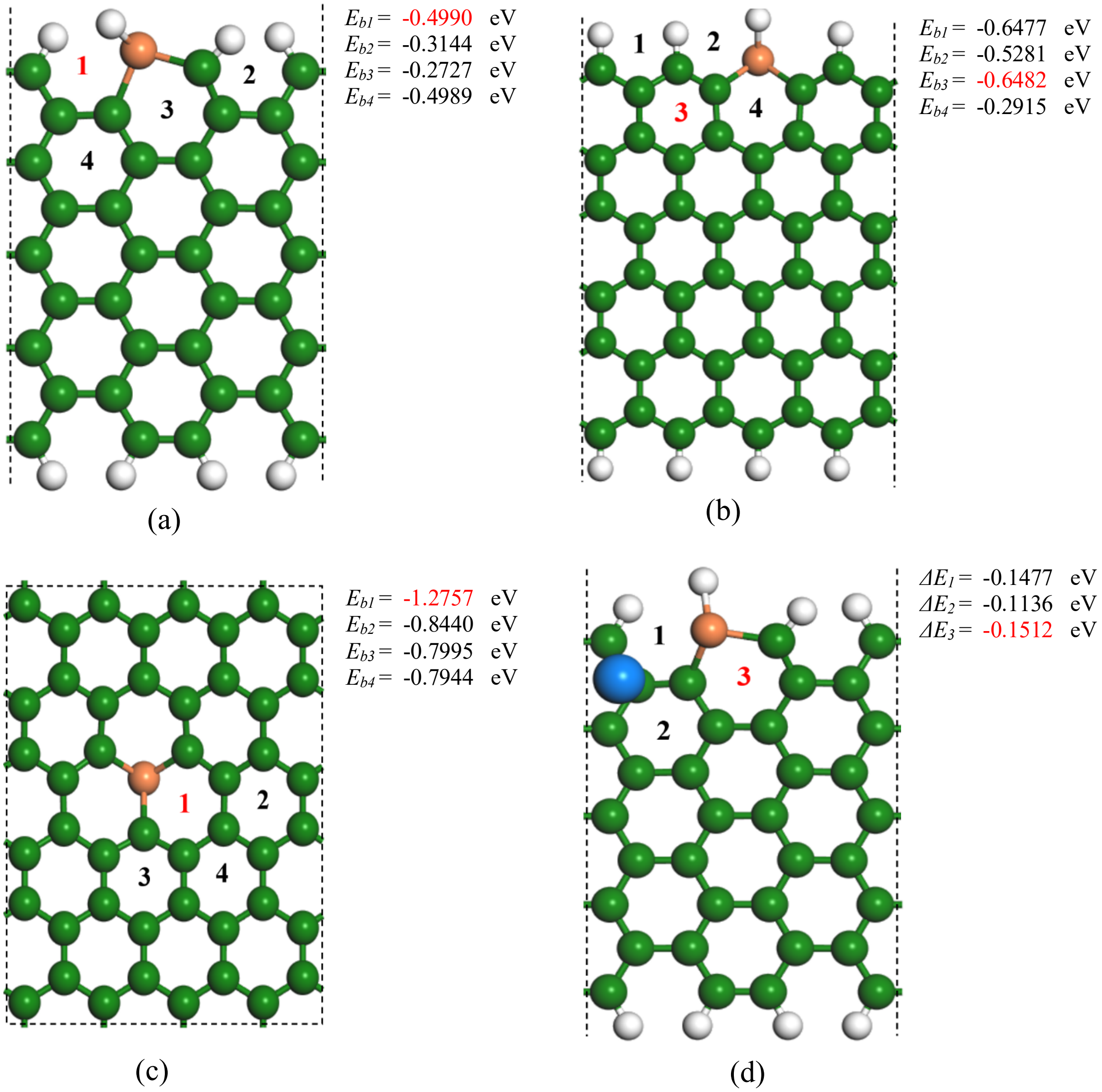

Supplementary Fig. 5 Investigated possible metal calcium atom (Ca) chemisorption sites and corresponding the binding energy upon adsorbing a calcium atom on hollow sites of (a) a-Si, (b) z-Si and (c) g-Si graphene models. And the most stable Si-doped graphene supported Ca single atom structure (a-Si-G-Ca) (d) selected from all Si-doped graphene supported Ca single-atom models was used to check the optimal H_2 molecule adsorption sites from all possible H_2 adsorption sites and the adsorption energy change before and after H_2 molecule adsorption was calculated.

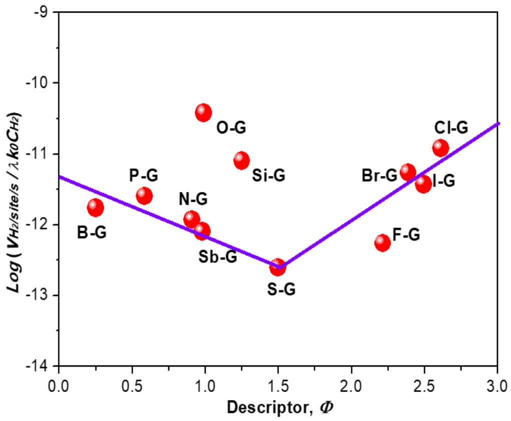

Supplementary Fig. 6 The volcano relationship between logarithm of parameter related to H_2 storage rate ($v_{H_2/site/s}/\lambda k_0 c_{H_2}$)and descriptor proposed *Φ*.

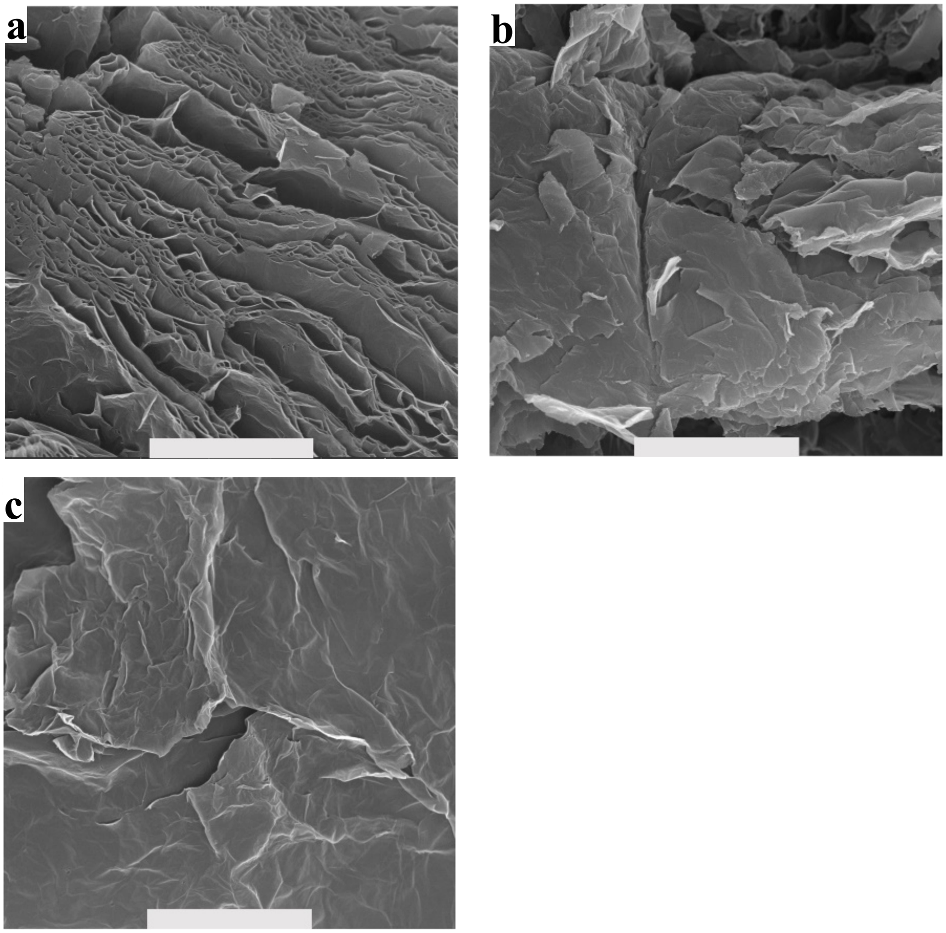

Supplementary Fig 7 Scanning electron microscopy images of different doped-graphene-supported Ca single atoms samples. O-, P-, and N-doped graphene with Ca single atoms samples corresponding to **a**, **b**, and **c**. (scale bar, 2 μm) These SEM images indicated that these carbon-based all-solid-state Ca single atom H_2 storage materials have similar morphology.

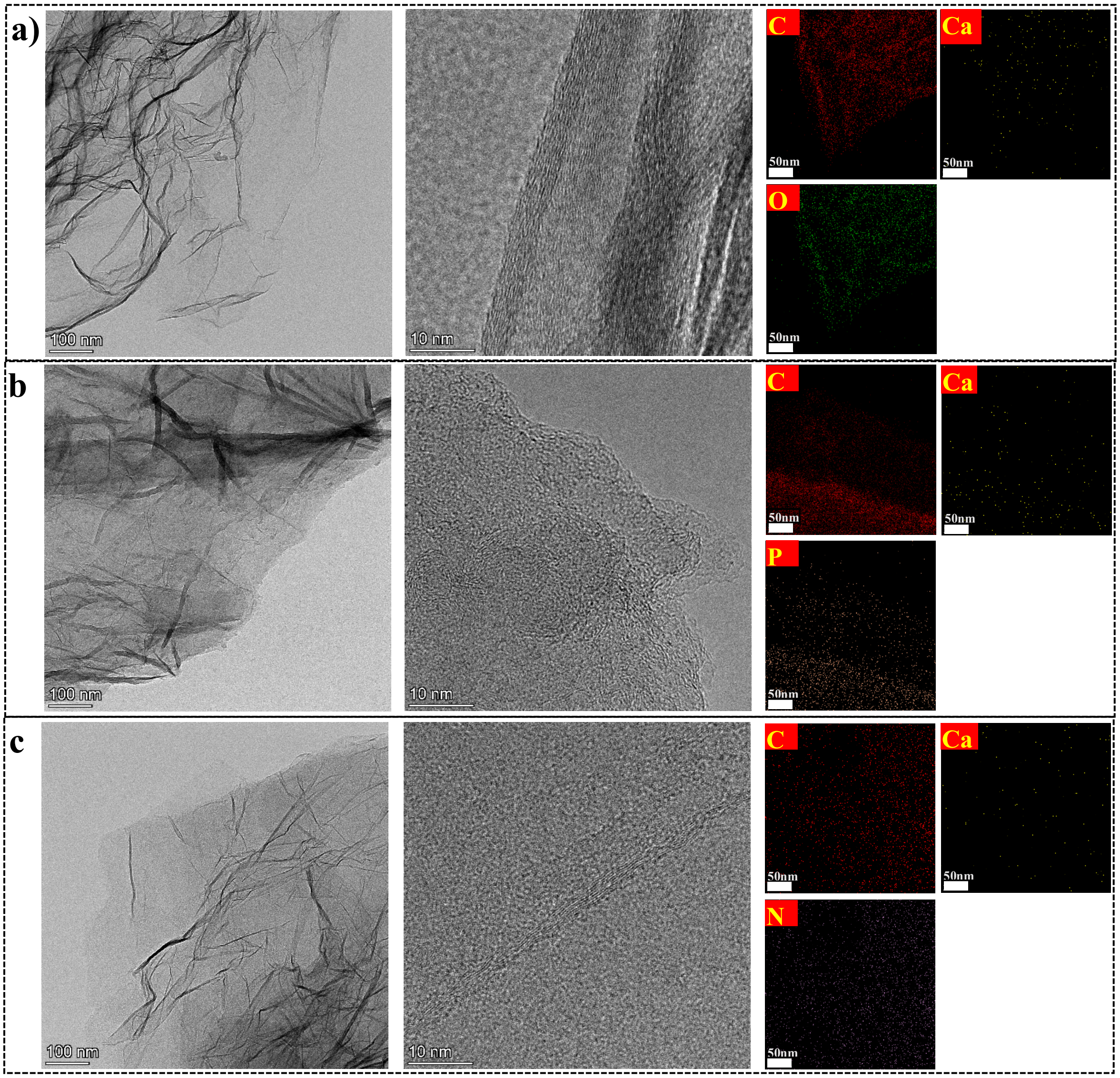

Supplementary Fig 8 Transmission electron microscopy (TEM) images, HR-TEM images and corresponding EDS elemental mapping of different doped-graphene-supported Ca single atoms samples. O-, P-, and N-doped graphene with Ca single atoms samples corresponding to **a**, **b**, and **c**. These TEM images indicated that these carbon-based all-solid-state Ca single atom H_2 storage materials have similar morphology once again.

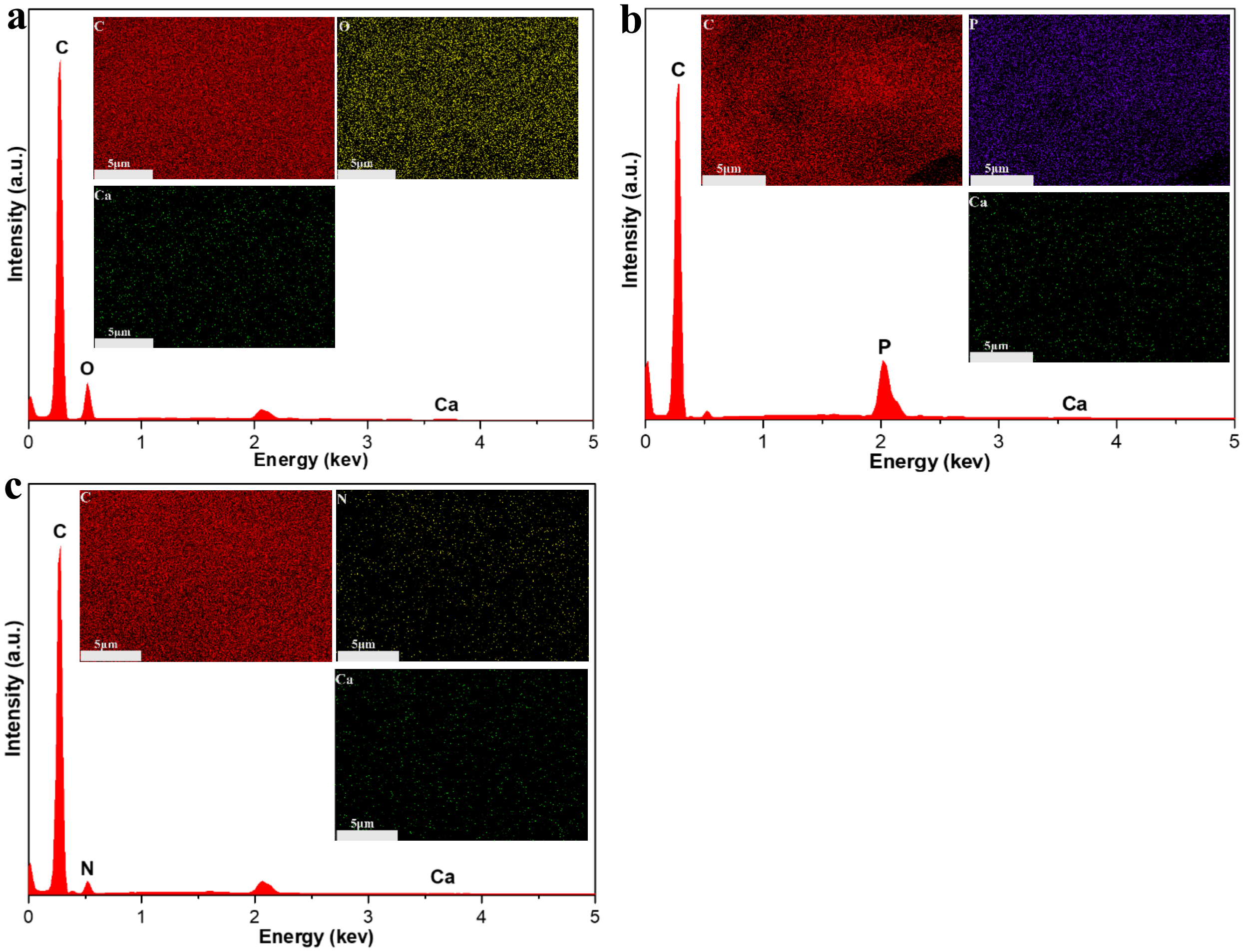

Supplementary Fig 9 Energy dispersive spectroscopy (EDS) measurements of different doped-graphene with Ca single atoms samples including O-, P-, and N-doped graphene with Ca single atoms samples corresponding to a, b and c. Inset are EDS elemental mappings of carbon, dopants and metal atoms including C, P, N, and Ca.

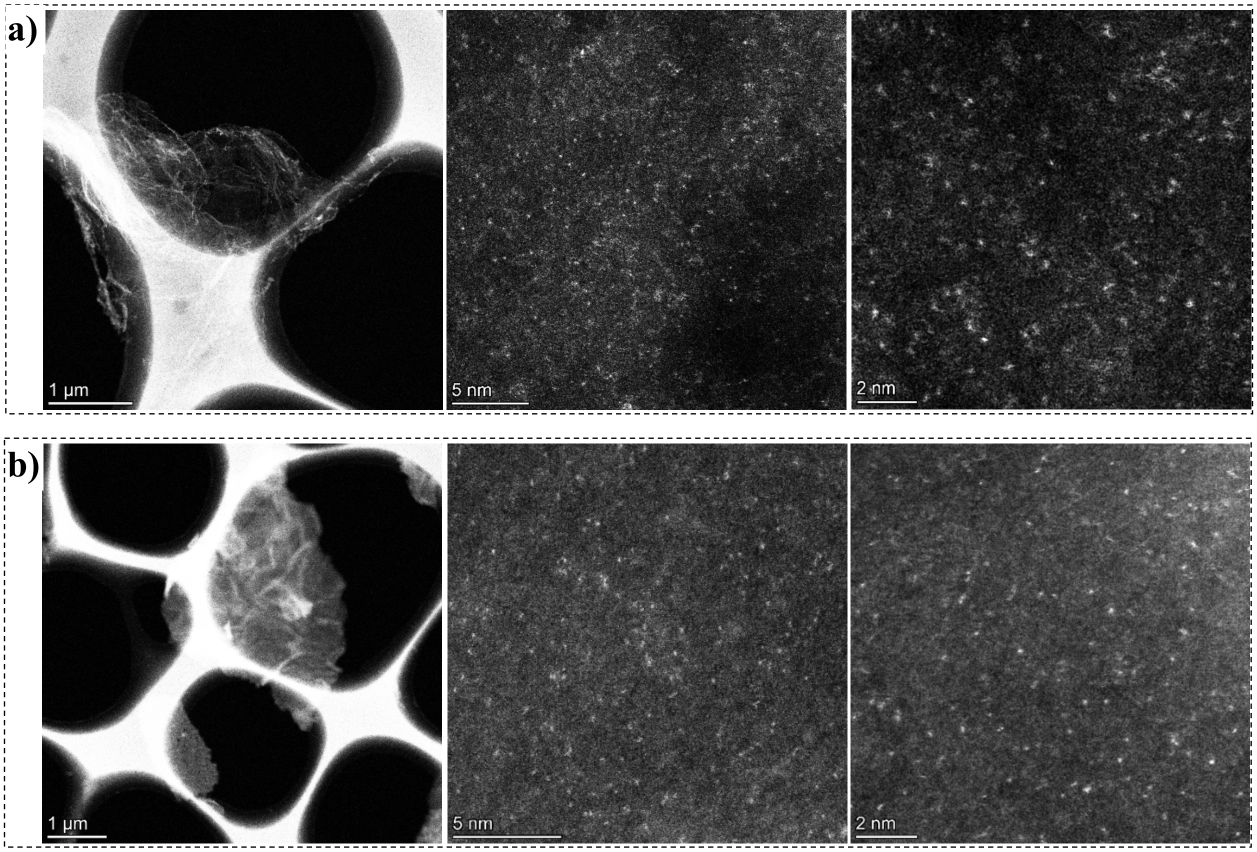

Supplementary Fig 10 Typical aberration corrected HAADF-STEM images of doped-graphene-supported Ca single atoms in different scale bars. a. and **b.** refer to N-G-Ca and P-G-Ca carbon-based Ca single-atom solid-state H_2 storage materials. Clearly, Ca can be dispersed atomically on doped-graphene-support.

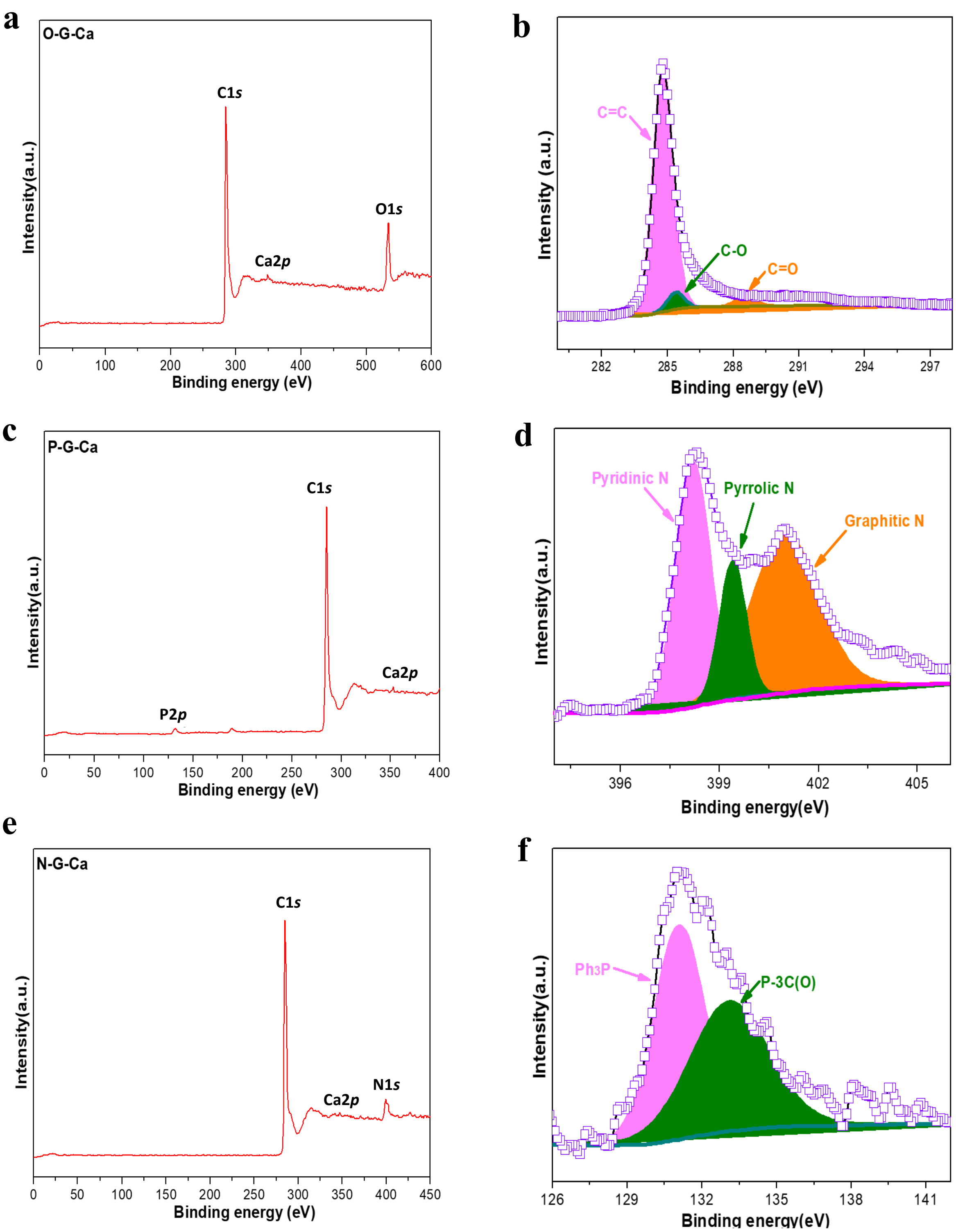

Supplementary Fig 11 Survey spectrum and high-resolution XPS spectra of heteroatoms in doped graphene samples. **a**, **c** and **e** correspond to survey spectrum of O-, P- and N-doped-graphene-supported Ca single atom samples, respectively. **b**, **d** and **f** correspond to high-resolution XPS spectra of O-, N- and P-doped graphene substrates.

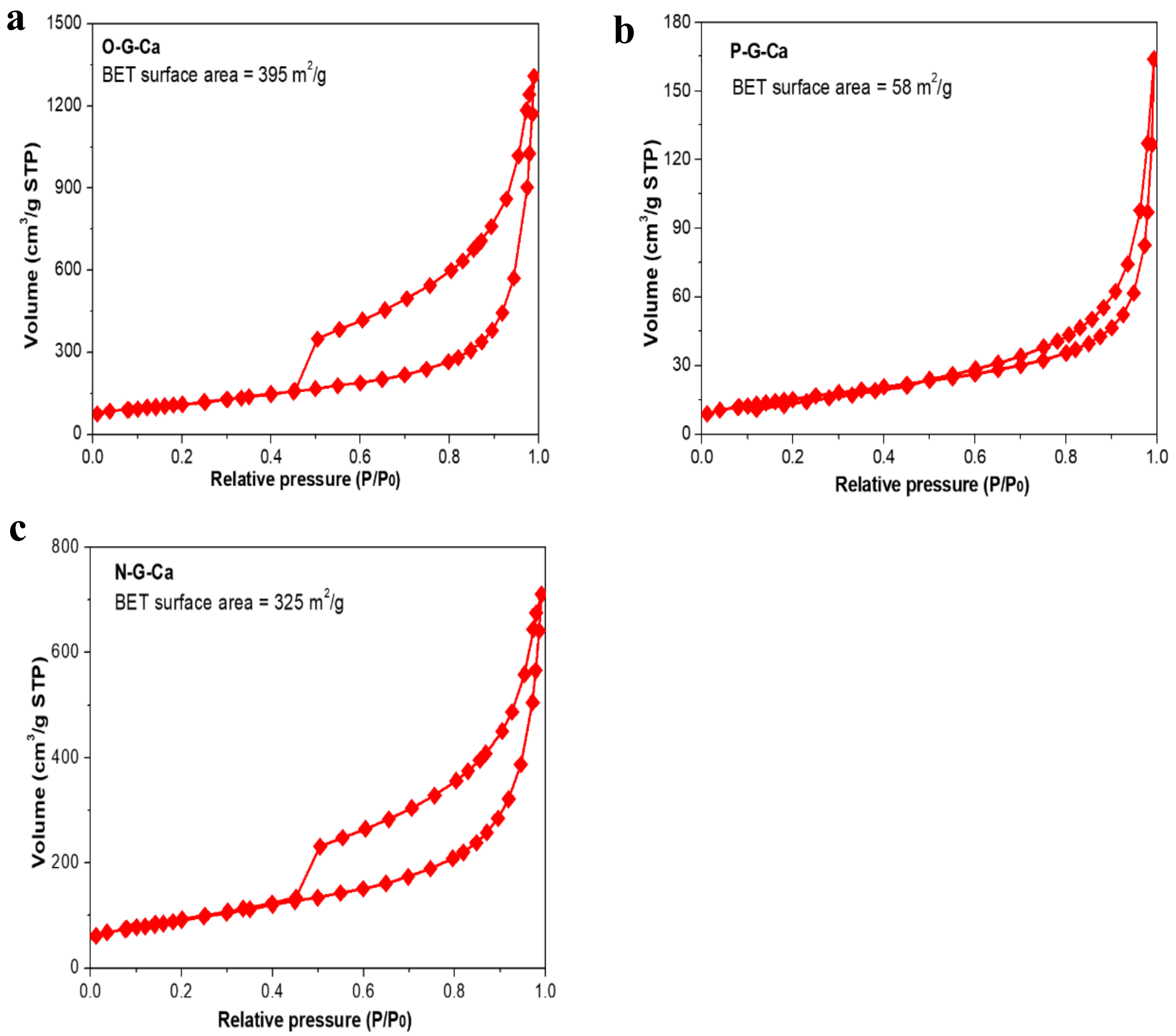

Supplementary Fig. 12 Nitrogen adsorption isotherms and BET surface areas for synthesized doped-graphene supported Ca single atoms. a. O-G-Ca, **b.** P-G-C and **c.** N-G-Ca sole-doped graphene with Ca single atoms used as carbon-based all-solid-state H_2 storage materials.

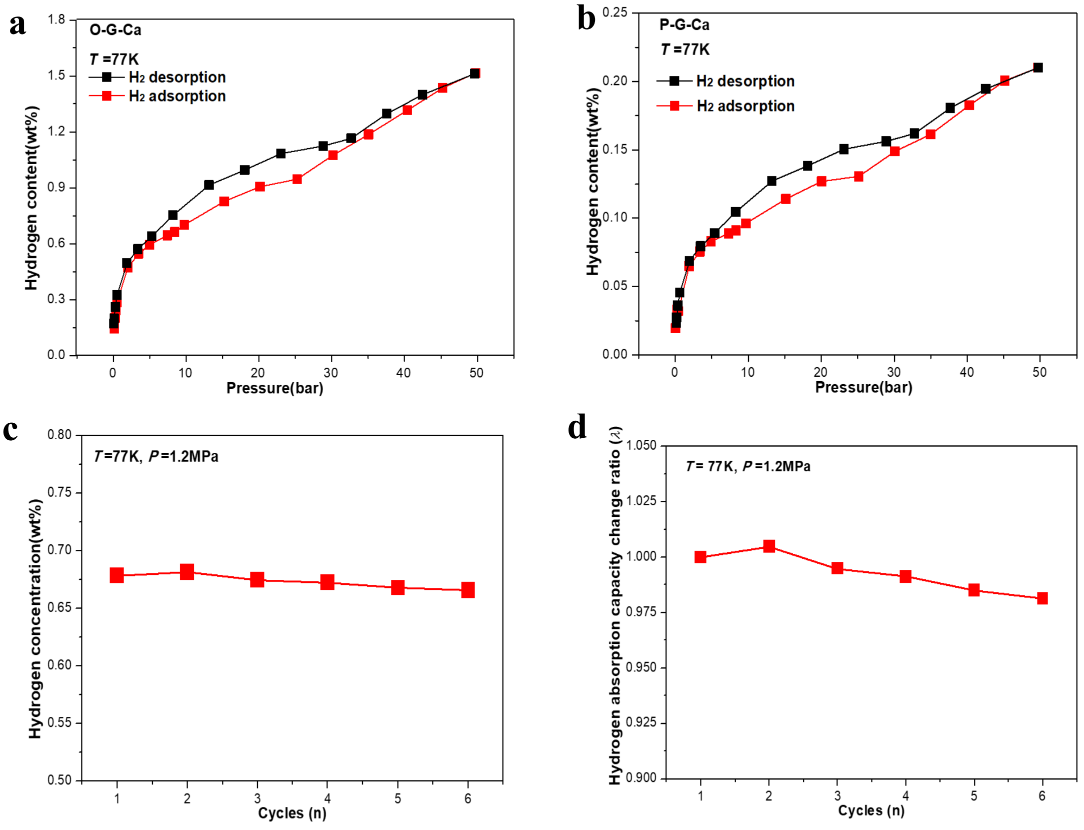

Supplementary Fig. 13 H_2 adsorption/desorption curve as function of pressure for sample a. O-G-Ca and b. P-G-Ca samples. c. hydrogen concentration (wt%) and d. hydrogen adsorption capacity change ratio (λ) as a function of cycle number (n) for representative sample N-G-Ca.

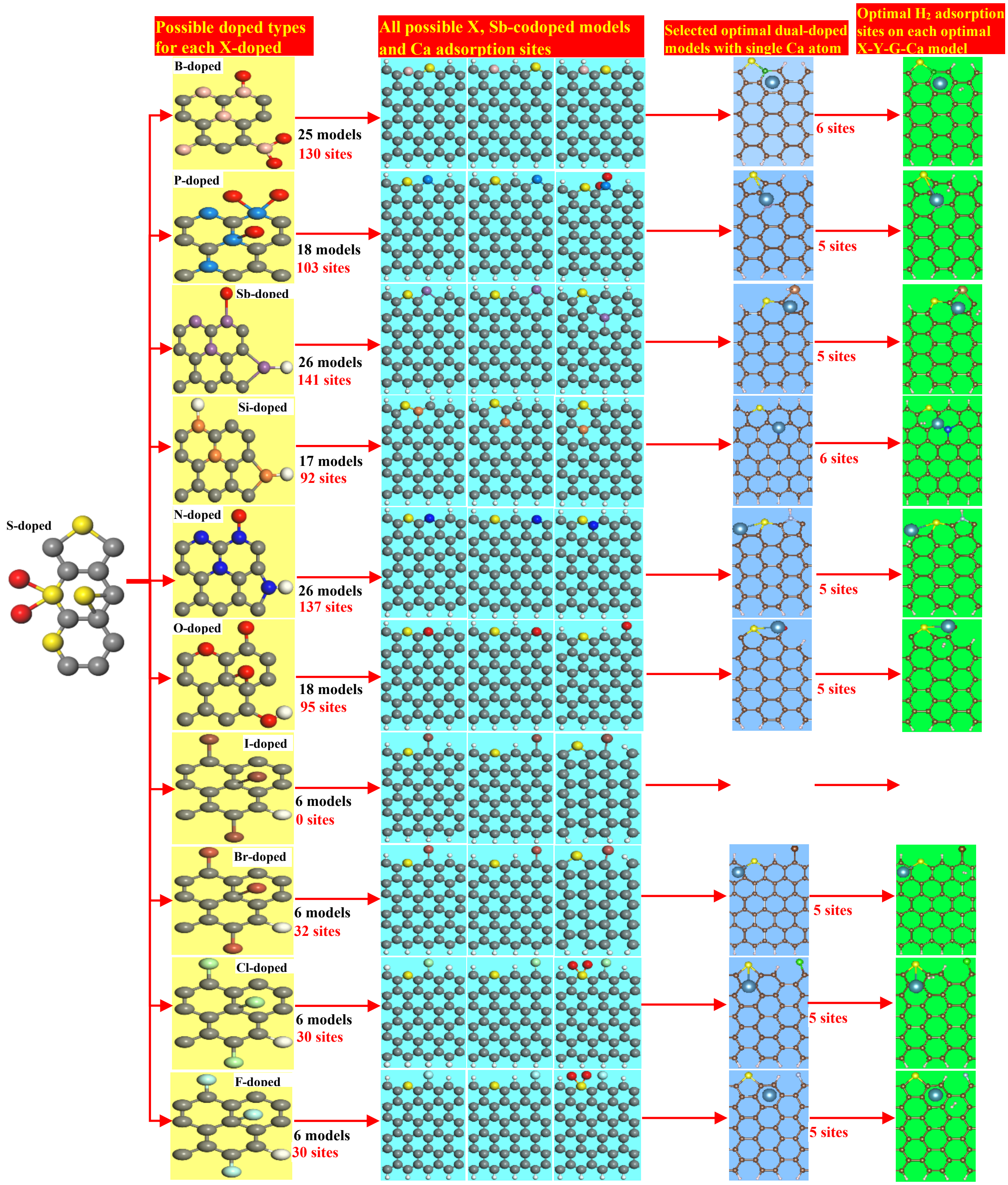

Supplementary Fig. 14 Selection process of optimal X, S-codoped graphene with Ca single atoms models and corresponding optimal H_2 adsorption sites. Here, all possible 154 models were constructed to cover each dual-doped configuration, and all possible 790 single atom Ca adsorption sites were tested via DFT calculation to screen out optimal X-Y-G-Ca models, which were used to search for optimal H_2 molecule adsorption sites from all possible 47 H_2 adsorption sites on selected optimal X-Y-G-Ca models. It deserves to note that all possible S-I-dual-doped models were tested to be unstable though DFT calculation, which cannot be employed to chemisorb single atom Ca.

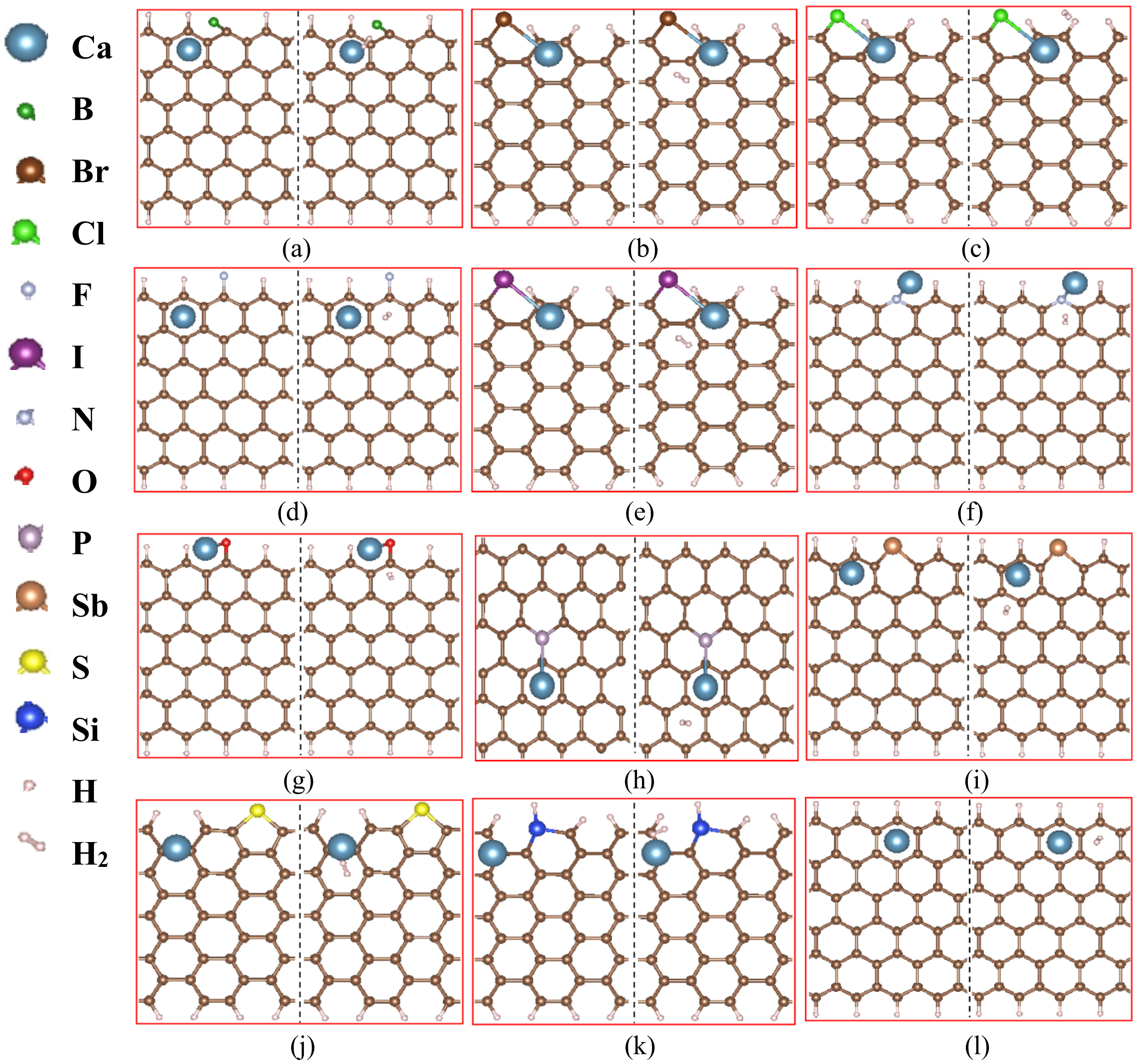

Supplementary Fig 15 Each optimal sole-doped graphene supported Ca single atom model where Ca was located on optimal active site and the corresponding optimal H_2 molecule adsorption site for (a) B=C-G-Ca, (b) a-Br-G -Ca, (c) a-Cl-G-Ca, (d) z-F-G-Ca, (e) a-I-G-Ca, (f) py-N-G-Ca, (g) py-O-G -Ca, (h) P-3C-G-Ca, (i) py-Sb-G-Ca, (j) th-S-G-Ca, (k) a-Si-G-Ca and (l) z-C-G-Ca models.

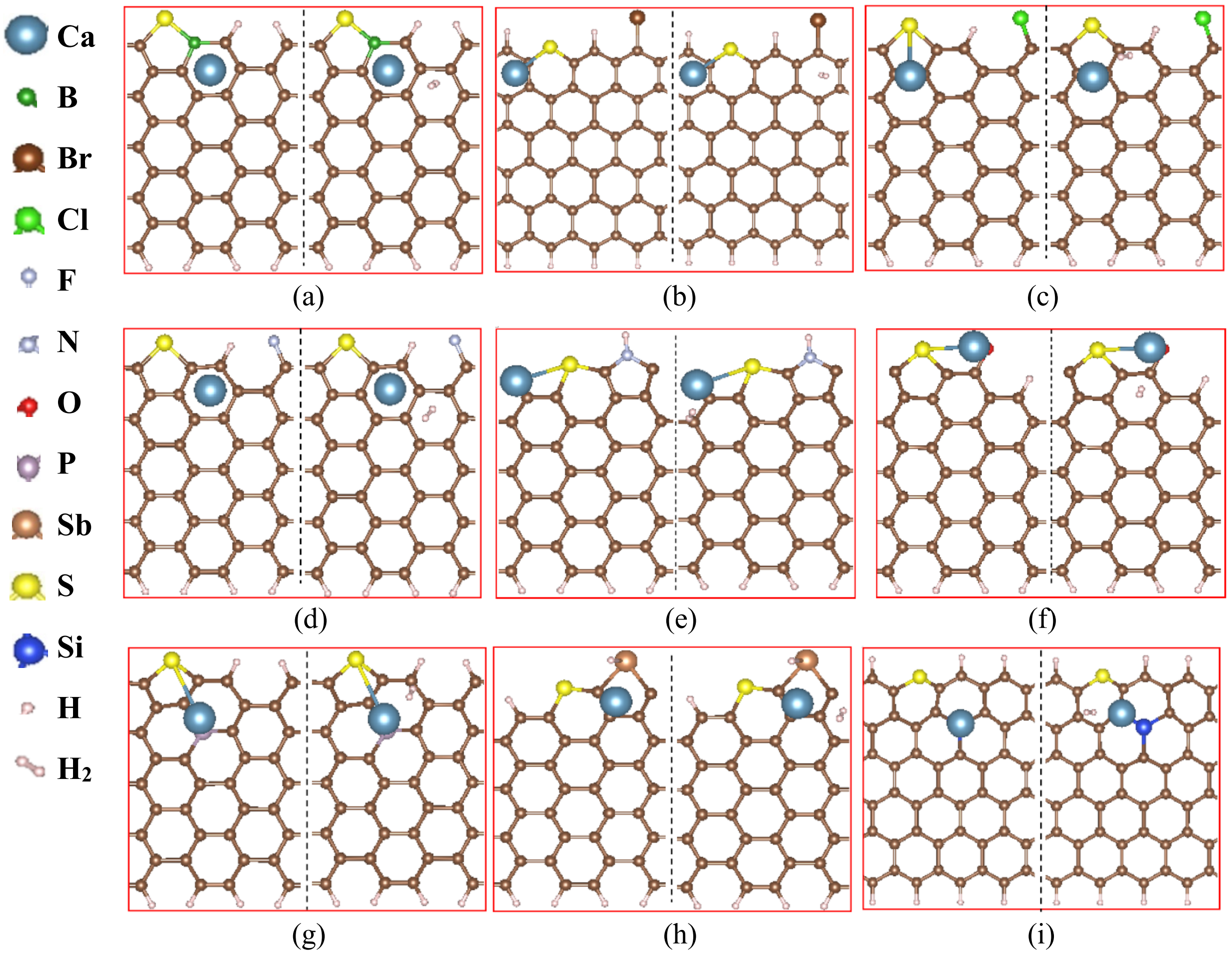

Supplementary Fig. 16 **Each optimal dual-doped graphene supported Ca single atom model where Ca was located on optimal active site and the corresponding optimal H_2 molecule adsorption site for (a) S-B-G-Ca, (b) S-Br-G-Ca, (c) S-Cl-G-Ca, (d) S-F-G-Ca, (e) S-N-G-Ca, (f) S-O-G-Ca, (g) S-P-G-Ca, (h) S-Sb-G-Ca and (i) S-Si-G-Ca dual-doped supported Ca single atom models.**

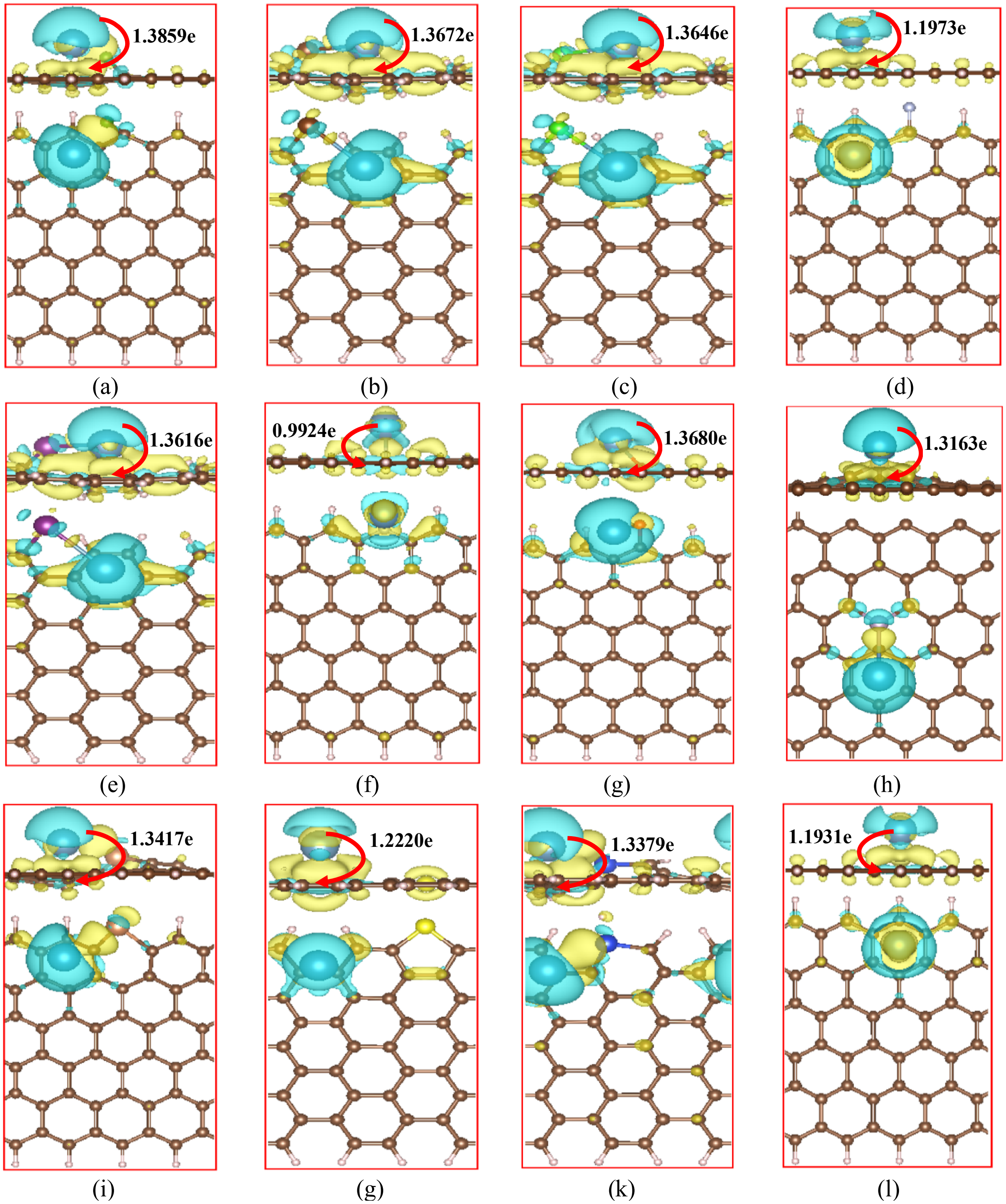

Supplementary Fig. 17 The charge transfer distribution and Bader charge on single atom Ca chemisorbed on optimal active site of each optimal sole-doped graphene model. The top view and side view of the electron charge distribution (a) B=C-G-Ca, (b) a-Br-G-Ca, (c) a-Cl-G-Ca, (d) z-F-G-Ca, (e) a-I-G-Ca, (f) py-N-G-Ca, (g) py-O-G-Ca, (h) P-3C-G-Ca, (i) py-Sb-G-Ca, (j) th-S-G-Ca, (k) a-Si-G-Ca and (l) z-C-G-Ca models. The blue and yellow colors indicate the positive and negative value of electron quantities, respectively, and red arrows imply the value of Bader charge transferred to substrate from Ca atom. The isosurface value is set to 0.003 e/Bohr^3.

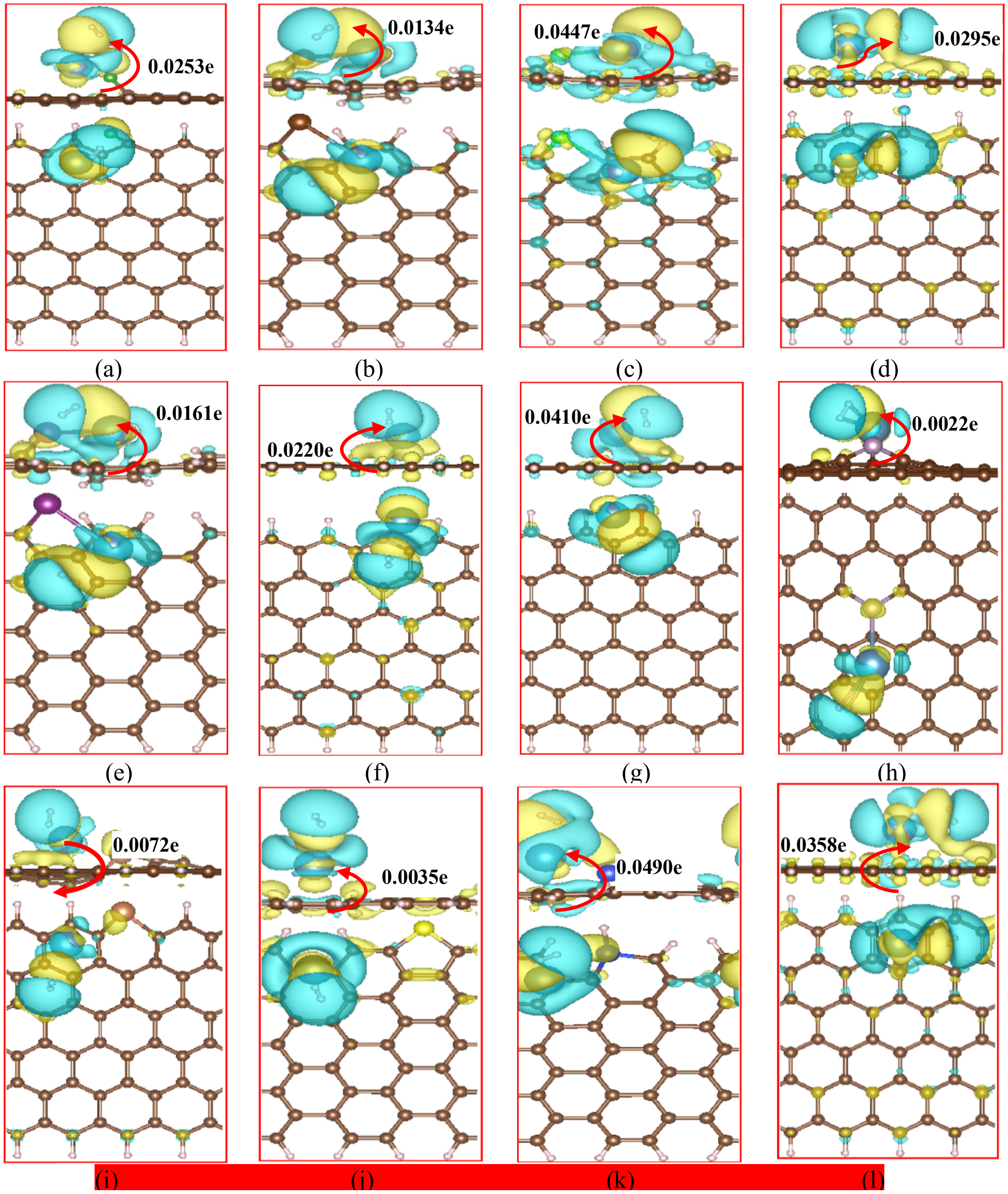

Supplementary Fig. 18 The charge transfer distribution and Bader charge on H_2 molecule adsorbed on optimal active site of each optimal sole-doped graphene model with single atom Ca. The top view and side view of the electron charge distribution (a) B=C-G-Ca(-H_2), (b) a-Br-G-Ca(-H_2), (c) a-Cl-G-Ca(-H_2), (d) z-F-G-Ca(-H_2), (e) a-I-G-Ca(-H_2), (f) py-N-G-Ca(-H_2), (g) py-O-G-Ca(-H_2), (h) P-3C-G-Ca(-H_2), (i) py-Sb-G-Ca(-H_2), (j) th-S-G-Ca(-H_2), (k) a-Si-G-Ca(-H_2) and (l) z-C-G-Ca(-H_2) models. The blue and yellow colors indicate the positive and negative value of electron quantities, respectively, and red arrows imply the value of Bader charge transferred to H_2 molecule from Ca atom. The isosurface value is set to 0.0002 e/Bohr^3.

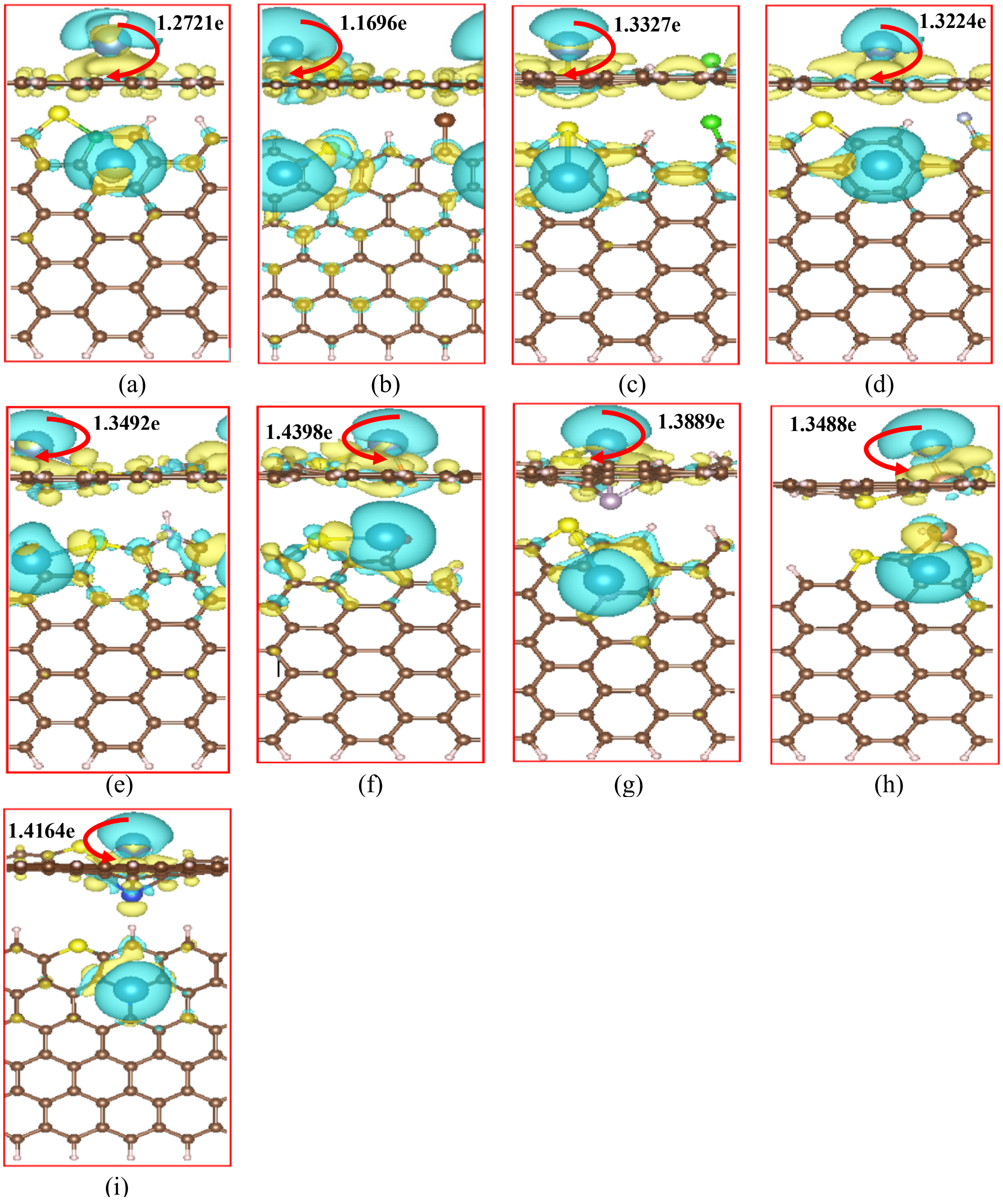

Supplementary Fig. 19 The charge transfer distribution and Bader charge on single atom Ca chemisorbed on optimal active site of each optimal dual-doped graphene model. The top view and side view of the electron charge distribution (a) S-B-G-Ca, (b) S-Br-G-Ca, (c) S-Cl-G-Ca, (d) S-F-G-Ca, (e) S-N-G-Ca, (f) S-O-G-Ca, (g) S-P-G-Ca, (h) S-Sb-G-Ca and (i) S-Si-G-Ca models. The blue and yellow colors indicate the positive and negative value of electron quantities, respectively, and red arrows imply the value of Bader charge transferred to substrate from Ca atom. The isosurface value is set to 0.003 e/Bohr^3.

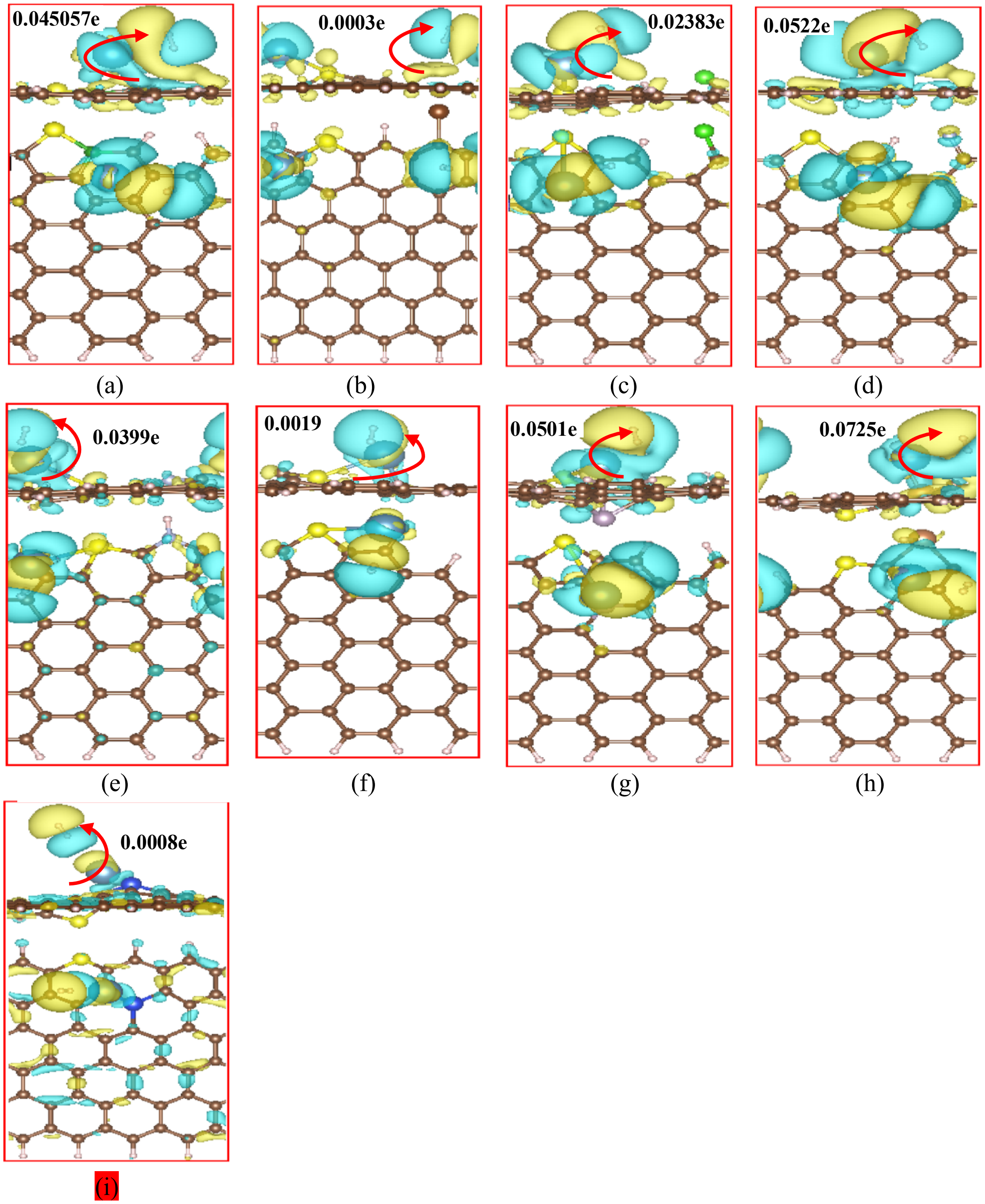

Supplementary Fig. 20 The charge transfer distribution and Bader charge on H_2 molecule adsorbed on optimal active site of each optimal dual-doped graphene model with single atom Ca. The top view and side view of the electron charge distribution (a) S-B-G-Ca(-H_2), (b) S-Br-G-Ca(-H_2), (c) S-Cl-G-Ca(-H_2), (d) S-F-G-Ca(-H_2), (e) S-N-G-Ca(-H_2), (f) S-O-G-Ca(-H_2), (g) S-P-G-Ca(-H_2), (h) S-Sb-G-Ca(-H_2) and (i) S-Si-G-Ca(-H_2) models. The blue and yellow colors indicate the positive and negative value of electron quantities, respectively, and red arrows imply the value of Bader charge transferred to H_2 molecule from Ca atom. The isosurface value is set to 0.0002 e/Bohr^3.

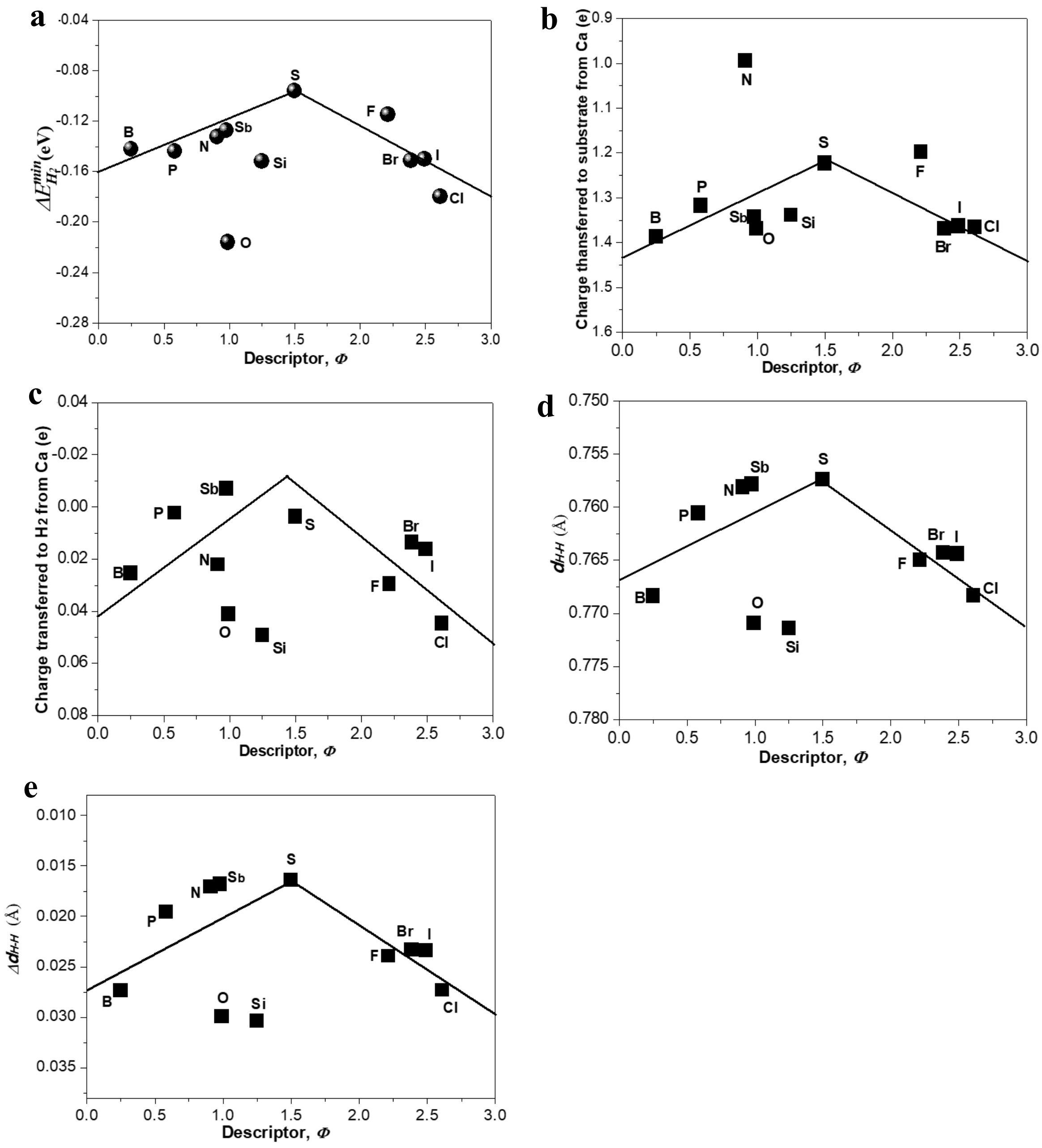

Supplementary Fig. 21 The relationships between descriptor *Φ* and related parameters extracted from each single-doped-graphene-supported Ca single-atom non-dissociative chemisorption solid-state H_2 storage materials structural model. a. relationship between the minimum H_2 adsorption energy change ($\Delta E_{H_2^*}^{min}$) and descriptor *Φ* for each doped-graphene with Ca single atoms. **b.** relationship between the charge transferred to doped-graphene substrate from Ca single atom and descriptor *Φ* for each doped-graphene with Ca single atoms. **c.** relationship between the charge transferred to H_2 adsorbed on optimal site from Ca single atom anchored doped-graphene substrate from and descriptor *Φ* for each doped-graphene with Ca single atoms. **d.** relationship between bond length (d_{H-H}) of H_2 molecule adsorbed on optimal site and descriptor *Φ* for each doped-graphene with Ca single atoms. **e.** relationship between bond length change (Δd_{H-H}) of H_2 molecule adsorbed on optimal site and descriptor *Φ* for each doped-graphene with Ca single atoms.

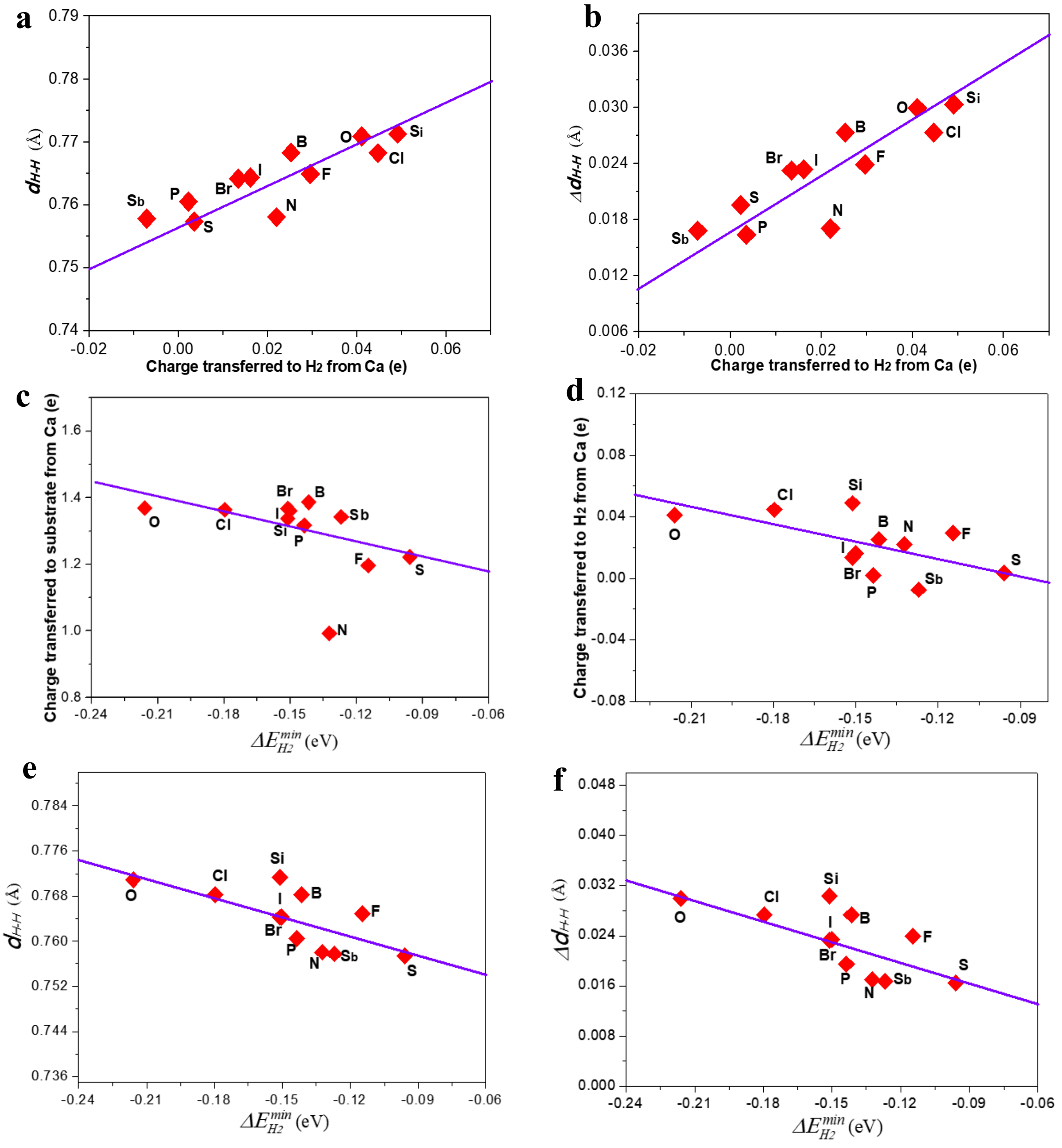

Supplementary Fig. 22 The relationships among related parameters extracted from each single-doped-graphene-supported Ca single-atom non-dissociative chemisorption solid-state H_2 storage materials structural model. a. relationship between $d_{H\text{-}H}$ and charge transferred to H_2 from Ca single atom for each doped-graphene with Ca single atoms. **b.** relationship between $d_{H\text{-}H}$ and charge transferred to H_2 from Ca single atom for each doped-graphene with Ca single atoms. **c.** relationship between the charge transferred to doped-graphene substrate from Ca single atom and $\Delta E_{H_2^*}^{min}$ for each doped-graphene with Ca single atoms. **d.** relationship between the charge transferred to H_2 molecule from Ca single atom and $\Delta E_{H_2^*}^{min}$ for each doped-graphene with Ca single atoms. **e.** relationship between $d_{H\text{-}H}$ and $\Delta E_{H_2^*}^{min}$ for each doped-graphene with Ca single atoms. f. relationship between $\Delta d_{H\text{-}H}$ and $\Delta E_{H_2^*}^{min}$ for each doped-graphene with Ca single atoms.

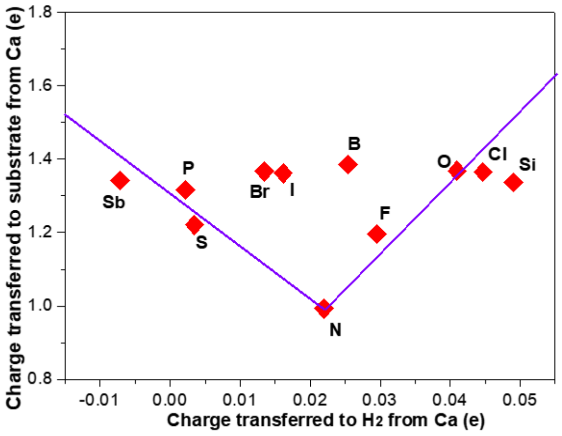

Supplementary Fig. 23 The relationships between charge transferred to doped-graphene substrate from Ca single atom and charge transferred to H_2 from Ca single atom for each single-doped-graphene-supported Ca single-atom non-dissociative chemisorption solid-state H_2 storage materials

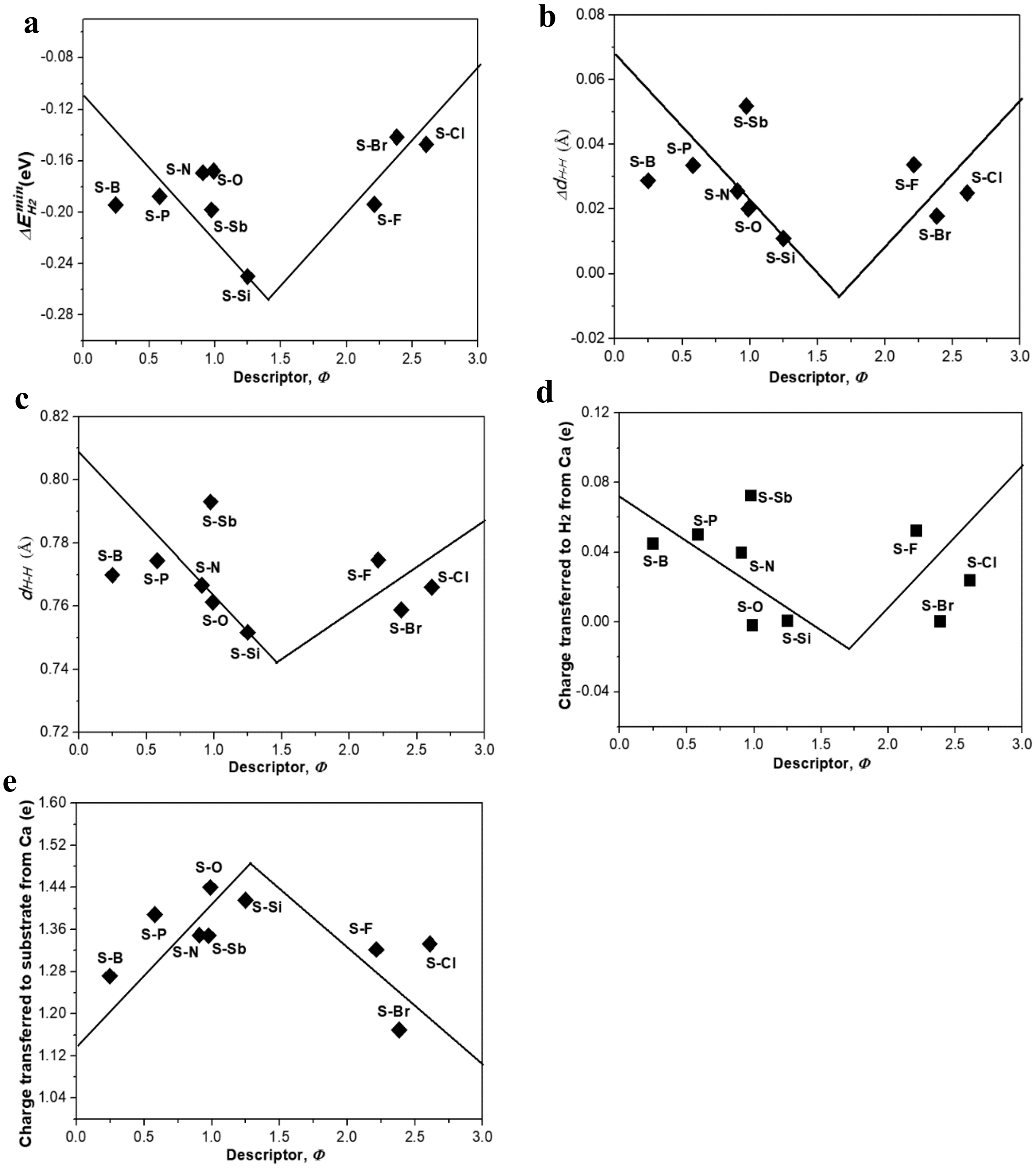

Supplementary Fig. 24 The relationships between descriptor *Φ* and related parameters extracted from each dual-doped-graphene-supported Ca single-atom non-dissociative chemisorption solid-state H_2 storage materials structural model. a. relationship between the minimum H_2 adsorption energy change ($\Delta E_{H_2^*}^{min}$) and descriptor *Φ* for each dual-doped-graphene with Ca single atoms. **b.** relationship between bond length change ($\Delta d_{H\text{-}H}$) of H_2 molecule adsorbed on optimal site and descriptor *Φ* for each dual-doped-graphene with Ca single atoms. **c.** relationship between bond length ($d_{H\text{-}H}$) of H_2 molecule adsorbed on optimal site and descriptor *Φ* for each dual-doped-graphene with Ca single atoms. **d.** relationship between the charge transferred to H_2 adsorbed on optimal site from Ca single atom anchored dual-doped-graphene substrate from and descriptor *Φ* for each dual-doped-graphene with Ca single atoms. **e.** relationship between the charge transferred to dual-doped-graphene substrate from Ca single atom and descriptor *Φ* for each dual-doped-graphene with Ca single atoms.

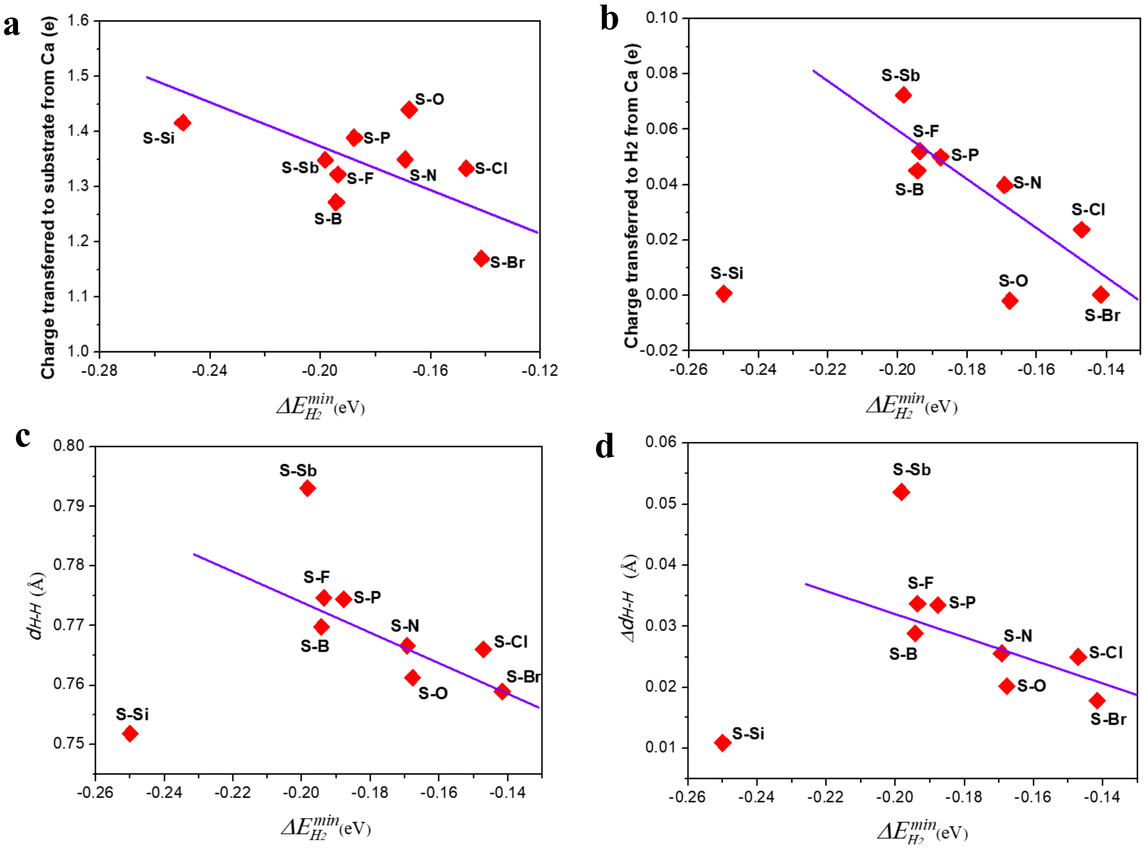

Supplementary Fig. 25 The relationships among related parameters extracted from each dual-doped-graphene-supported Ca single-atom non-dissociative chemisorption solid-state H_2 storage materials structural model. **a.** relationship between the charge transferred to dual-doped-graphene substrate from Ca single atom and $\Delta E_{H_2^*}^{min}$ for each dual-doped-graphene supported Ca single-atom model. **b.** relationship between the charge transferred to H_2 molecule from Ca single atom and $\Delta E_{H_2^*}^{min}$ for each dual-doped-graphene with Ca single atoms. **c.** relationship between $d_{H\text{-}H}$ and $\Delta E_{H_2^*}^{min}$ for each dual-doped-graphene with Ca single atoms. **d.** relationship between $\Delta d_{H\text{-}H}$ and $\Delta E_{H_2^*}^{min}$ for each dual-doped-graphene supported Ca single-atom model.

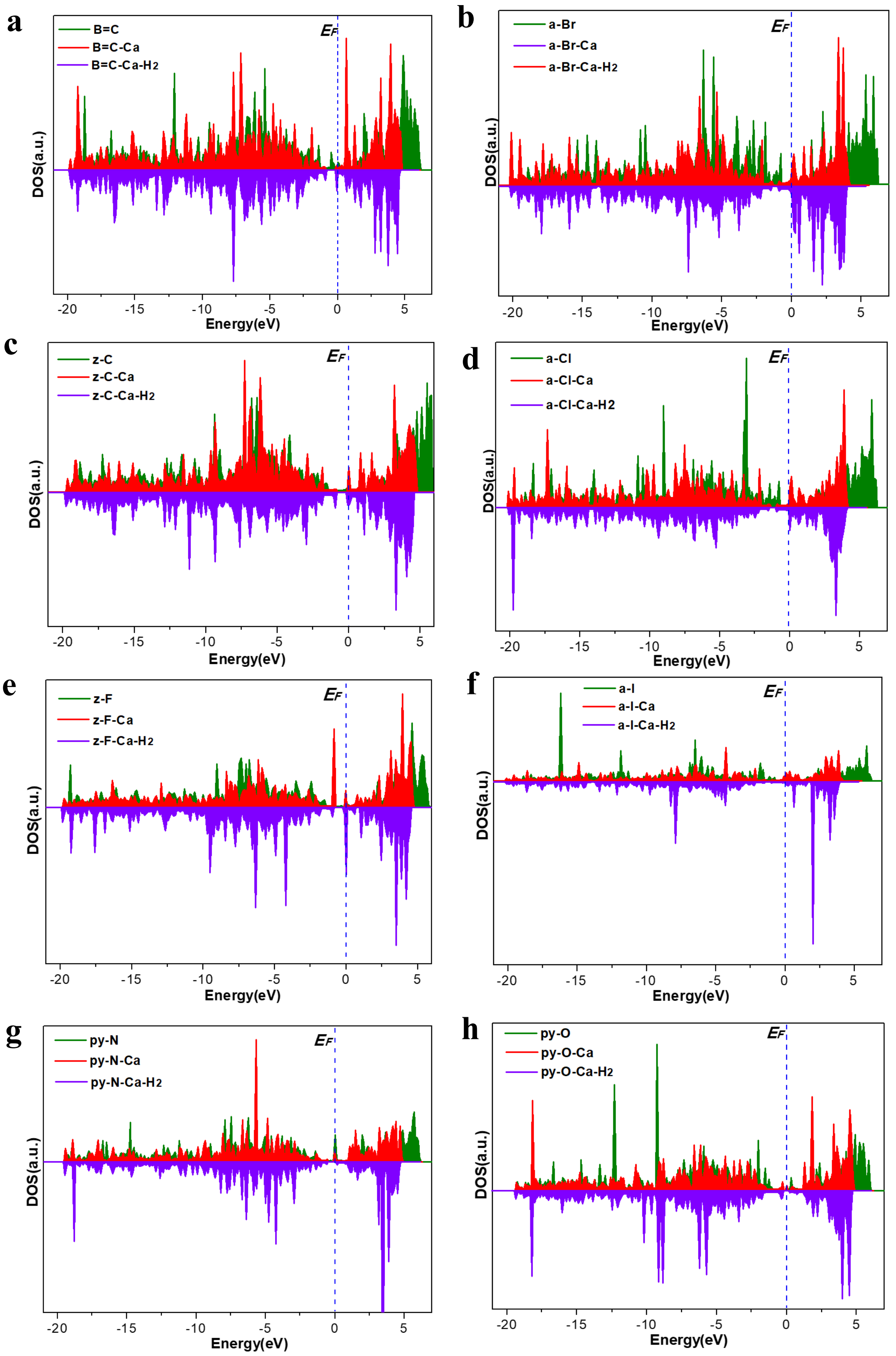
a
B=C
B=C-Ca
B=C-Ca-H2
EF
DOS(a.u.)
Energy(eV)
b
a-Br
a-Br-Ca
a-Br-Ca-H2
EF
DOS(a.u.)
Energy(eV)
c
z-C
z-C-Ca
z-C-Ca-H2
EF
DOS(a.u.)
Energy(eV)
d
a-Cl
a-Cl-Ca
a-Cl-Ca-H2
EF
DOS(a.u.)
Energy(eV)
e
z-F
z-F-Ca
z-F-Ca-H2
EF
DOS(a.u.)
Energy(eV)
f
a-I
a-I-Ca
a-I-Ca-H2
EF
DOS(a.u.)
Energy(eV)
g
py-N
py-N-Ca
py-N-Ca-H2
EF
DOS(a.u.)
Energy(eV)
h
py-O
py-O-Ca
py-O-Ca-H2
EF
DOS(a.u.)
Energy(eV)
-20
-15
-10
-5
0
5

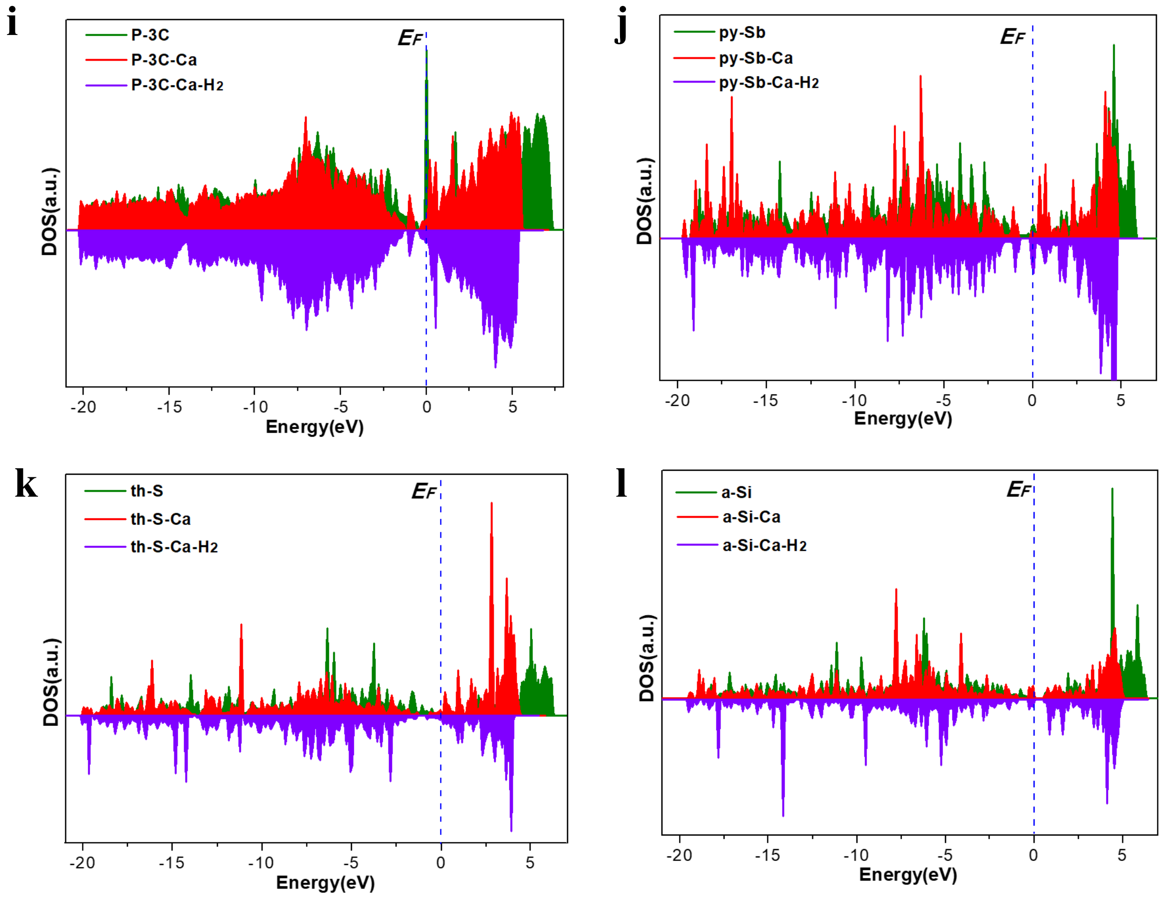

Supplementary Fig. 26 The DOSs of each optimal single-doped-graphene substrate (X-G), single-doped-graphene supported Ca single-atom mode (X-G-Ca) and single-doped-graphene supported Ca single-atom mode (X-G-Ca) with H_2 molecule adsorbed on optimal site (X-G-Ca-H_2). a. DOSs of B=C, B=C-Ca and B=C-Ca-H_2. **b.** DOSs of a-Br, a-Br-Ca and a-Br-Ca-H_2. **c.** DOSs of z-C, z-C-Ca and z-C-Ca-H_2. **d.** DOSs of a-Cl, a-Cl-Ca and a-Cl-Ca-H_2. **e.** DOSs of z-F, z-F-Ca and z-F-Ca-H_2. **f.** DOSs of a-I, a-I-Ca and a-I-Ca-H_2. **g.** DOSs of py-N, py-N-Ca and py-N-Ca-H_2. **h.** DOSs of py-O, py-O-Ca and py-O-Ca-H_2. **i.** DOSs of P-3C, P-3C and P-3C-Ca-H_2. **j.** DOSs of py-Sb, py-O-Sb and py-O-Ca-Sb. **k.** DOSs of th-S, th-S-Ca and th-S-Ca-H_2. **l.** DOSs of a-Si, a-Si-Ca and a-Si-Ca-H_2.

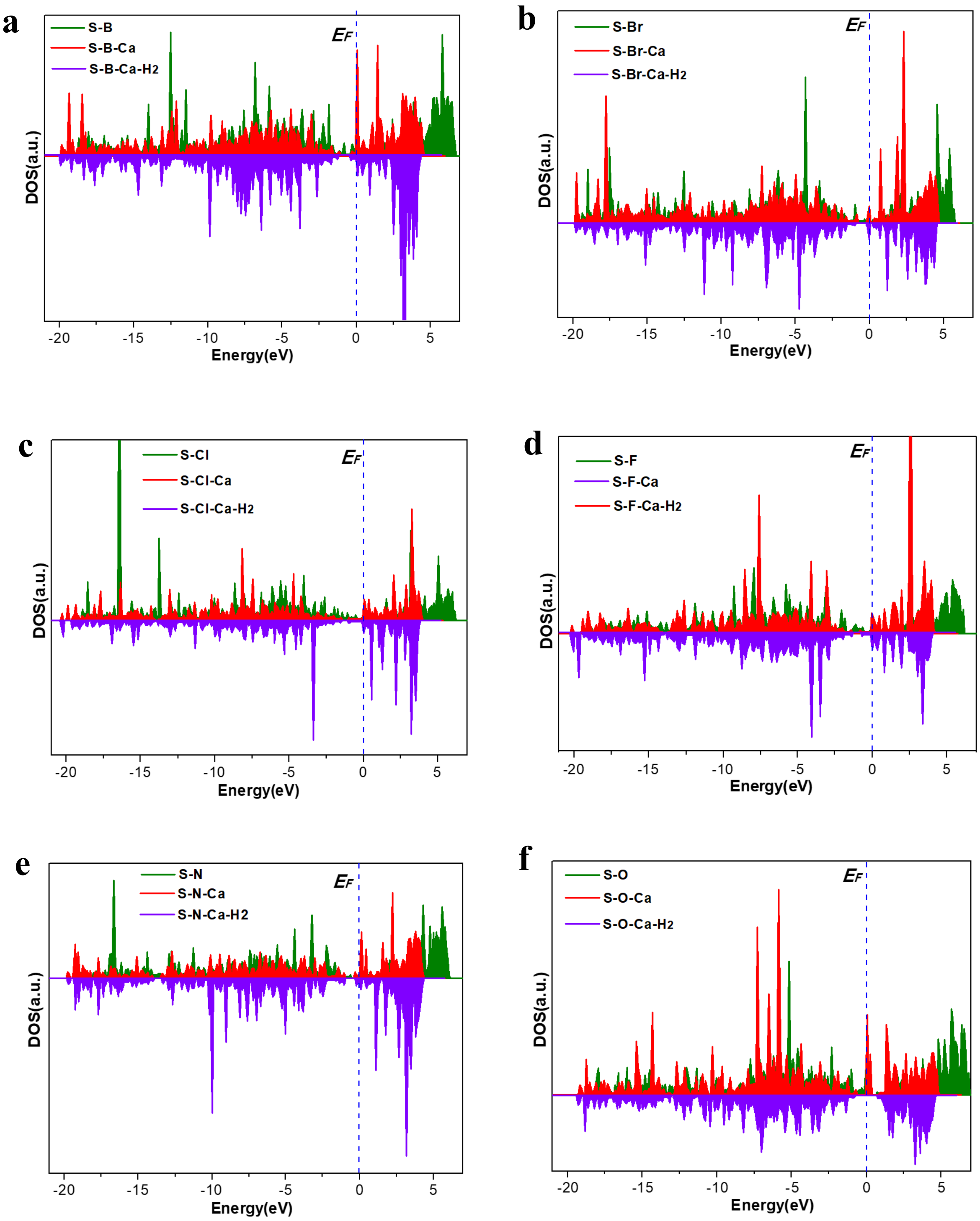
a
S-B
S-B-Ca
S-B-Ca-H2
EF
DOS(a.u.)
Energy(eV)
b
S-Br
S-Br-Ca
S-Br-Ca-H2
EF
DOS(a.u.)
Energy(eV)
c
S-Cl
S-Cl-Ca
S-Cl-Ca-H2
EF
DOS(a.u.)
Energy(eV)
d
S-F
S-F-Ca
S-F-Ca-H2
EF
DOS(a.u.)
Energy(eV)
e
S-N
S-N-Ca
S-N-Ca-H2
EF
DOS(a.u.)
Energy(eV)
f
S-O
S-O-Ca
S-O-Ca-H2
EF
DOS(a.u.)
Energy(eV)
-20 -15 -10 -5 0 5

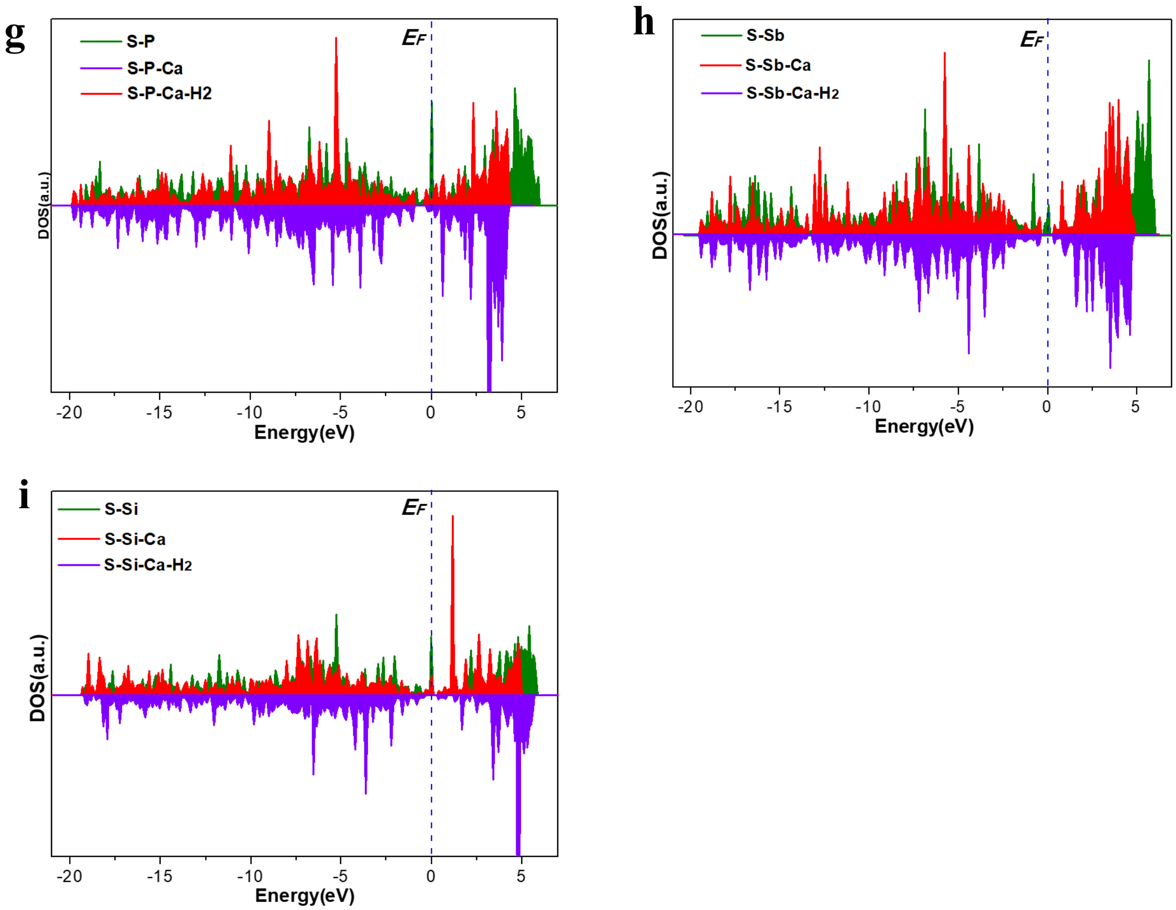

Supplementary Fig. 27 The DOSs of each optimal dual-doped-graphene substrate (X-Y-G), single-doped-graphene supported Ca single-atom mode (X-Y-G-Ca) and single-doped-graphene supported Ca single-atom mode (X-Y-G-Ca) with H_2 molecule adsorbed on optimal site (X-Y-G-Ca-H_2). a. DOSs of S-B-G, S-B-G-Ca and S-B-G-Ca-H_2. **b.** DOSs of S-Br-G, S-Br-G-Ca and S-Br-G-Ca-H_2. **c.** DOSs of S-Cl-G, S-Cl-G-Ca and S-Cl-G-Ca-H_2. **d.** DOSs of S-F-G, S-F-G-Ca and S-F-G-Ca-H_2. **e.** DOSs of S-N-G, S-N-G-Ca and S-N-G-Ca-H_2. **f.** DOSs of S-O-G, S-O-G-Ca and S-O-G-Ca-H_2. **g.** DOSs of S-P-G, S-P-G-Ca and S-P-G-Ca-H_2. **h.** DOSs of S-Sb-G, S-Sb-G-Ca and S-Sb-G-Ca-H_2. **i.** DOSs of S-Si-G, S-Si-G-Ca and S-Si-G-Ca-H_2.

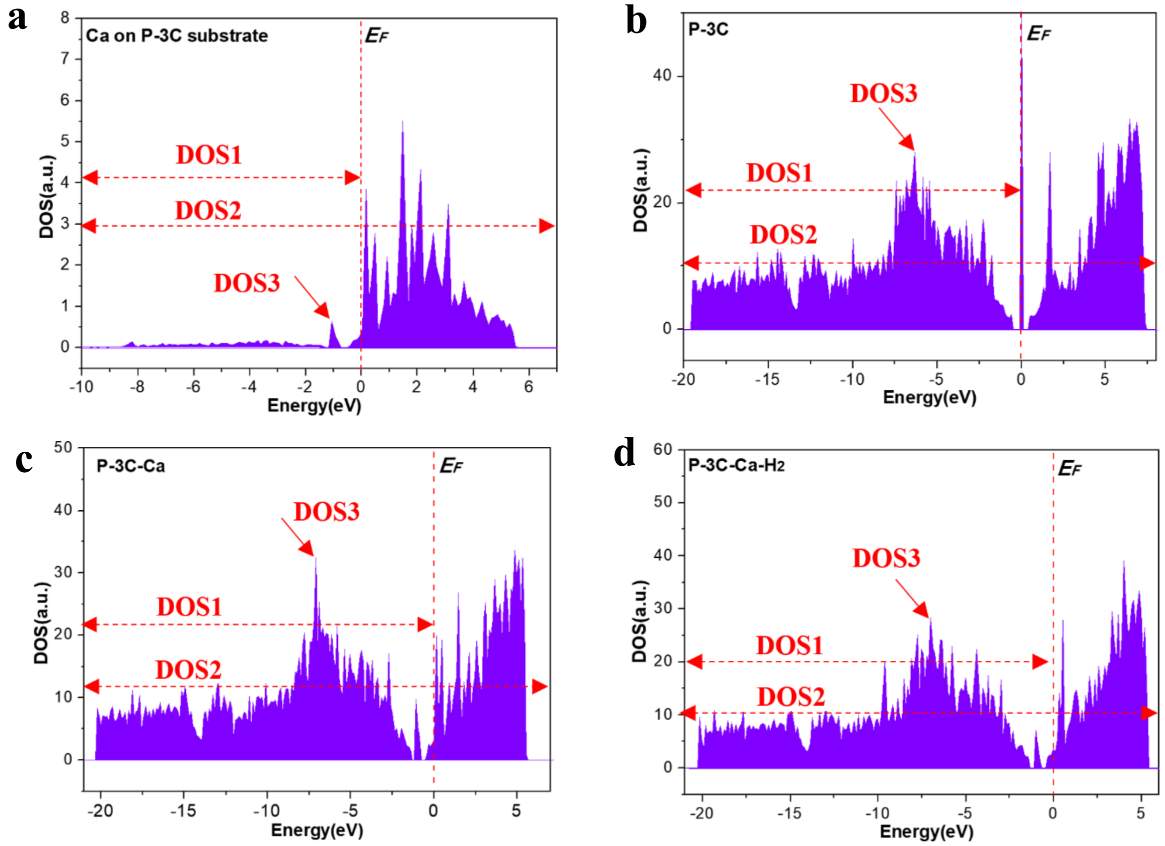

Supplementary Fig. 28 The definitions of three densities of electronic states (DOSs) evaluating the relationship between electronic structure and intrinsic descriptor Φ. As mentioned in previous work[2-3], the three DOSs are expressed as DOS1(the weighted DOS center below Fermi level), DOS2 (the weighted DOS center in overall range of energy) and DOS3 (the position of the highest peak of DOS below Fermi level). **a.** Three DOSs including DOS1, DOS2 and DOS3 of Ca single atom on single-/dual-doped-graphene substrate. **b.** Three DOSs including DOS1, DOS2 and DOS3 of single-/dual-doped-graphene substrate. **c.** Three DOSs including DOS1, DOS2 and DOS3 of single-/dual-doped-graphene-supported Ca single-atom model. **d.** Three DOSs including DOS1, DOS2 and DOS3 of single-/dual-doped-graphene-supported Ca single-atom model with H_2 molecule adsorbed on optimal site. Here, the calculation equation of weighted DOS was given as DOS=$(\Sigma_i \varepsilon_i d_i)/(\Sigma_i \varepsilon_i)$, where d_i is the DOS of each model at energy ε_i.

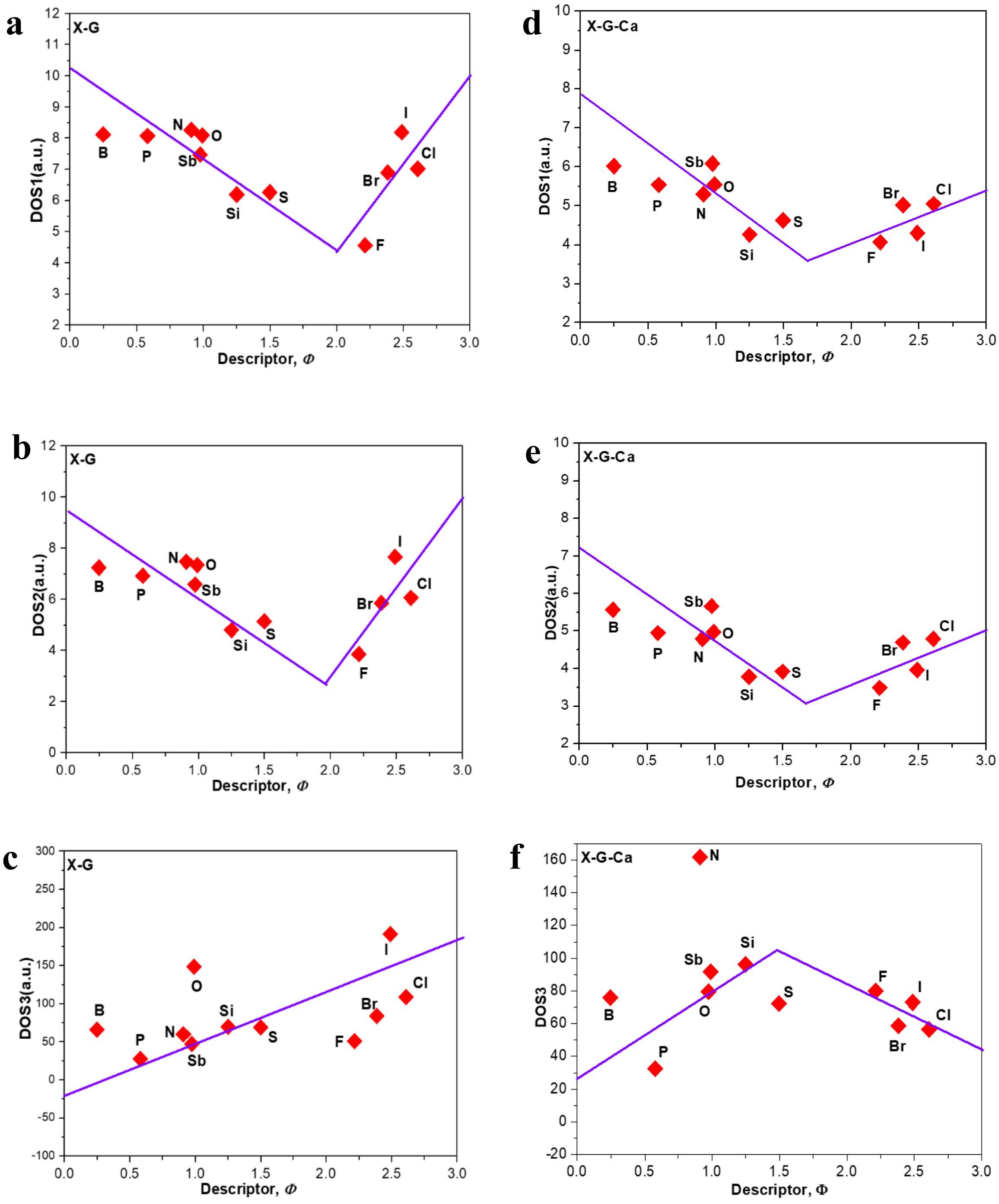

a
X-G
DOS1(a.u.)
Descriptor, Φ
B P N O Sb Si S I Cl Br F
d
X-G-Ca
DOS1(a.u.)
Descriptor, Φ
B P Sb N O Si S Br Cl F I
b
X-G
DOS2(a.u.)
Descriptor, Φ
B P N O Sb Si S I Cl Br F
e
X-G-Ca
DOS2(a.u.)
Descriptor, Φ
B P Sb N O Si S Br Cl F I
c
X-G
DOS3(a.u.)
Descriptor, Φ
B P O N Sb Si S I Cl Br F
f
X-G-Ca
DOS3
Descriptor, Φ
N B P Sb O Si S F I Cl Br

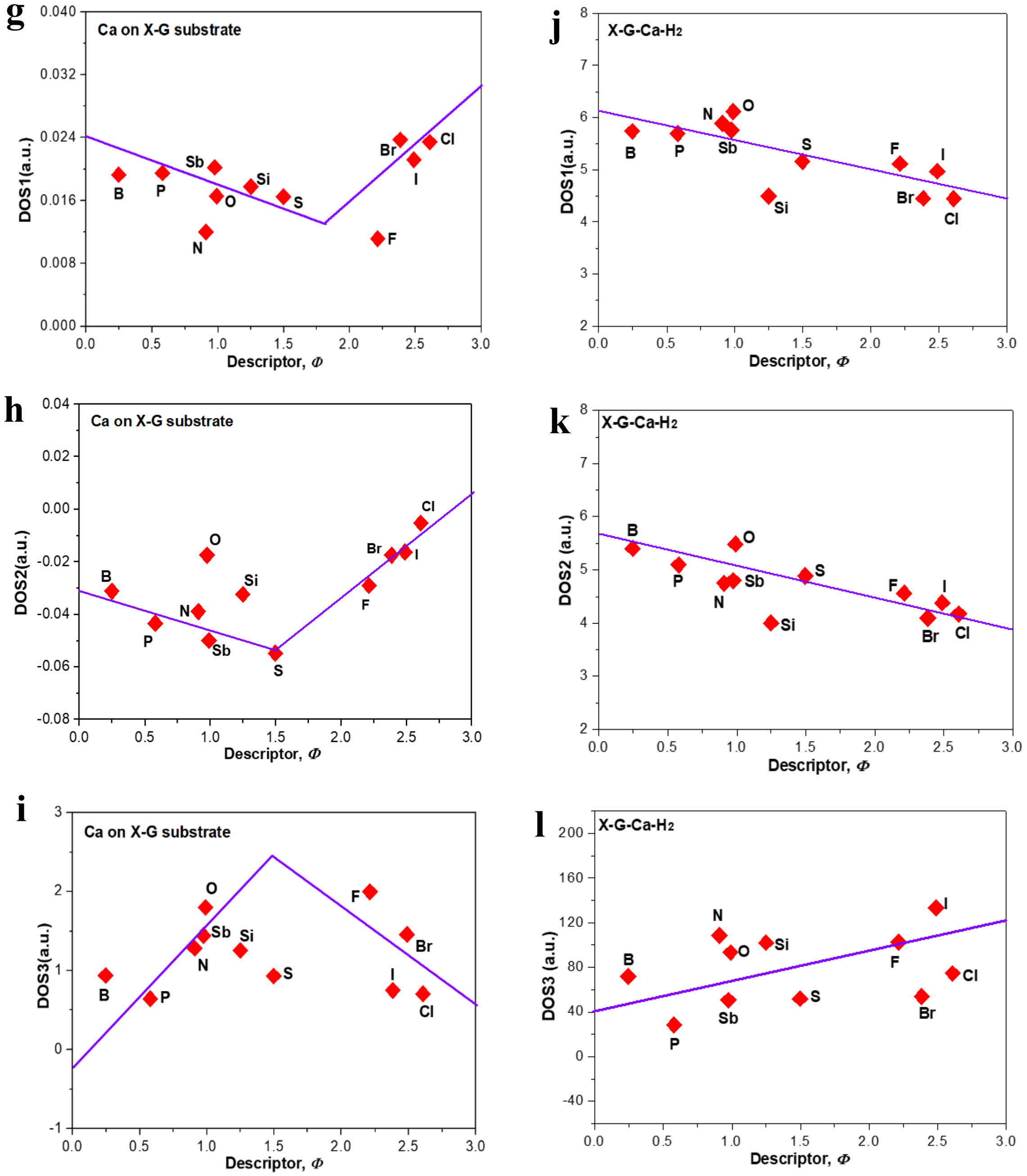

Supplementary Fig. 29 The relationships between DOS descriptors and intrinsic descriptor *Φ* for single-doped-graphene-supported Ca single-atom solid-state H_2 storage materials. a. b. and **c.** the relationships between DOS descriptors and intrinsic descriptor *Φ* for each optimal single-doped-graphene substrate (X-G). **d. e.** and **f.** the relationships between DOS descriptors and intrinsic descriptor *Φ* for the corresponding optimal single-doped- graphene with Ca atom chemisorption on optimal active site (X-G-Ca). **g. h.** and **i.** the relationships between DOS descriptors and intrinsic descriptor *Φ* for the corresponding Ca single atom anchored optimal single-doped-graphene substrate (Ca). **j. k.** and **l.** the relationships between DOS descriptors and intrinsic descriptor *Φ* for the corresponding optimal single-doped-graphene-supported Ca single-atom substrate with H_2 molecule adsorbed on optimal H2 storage site. Here, DOS descriptors consisted of DOS1, DOS2 and DOS3.

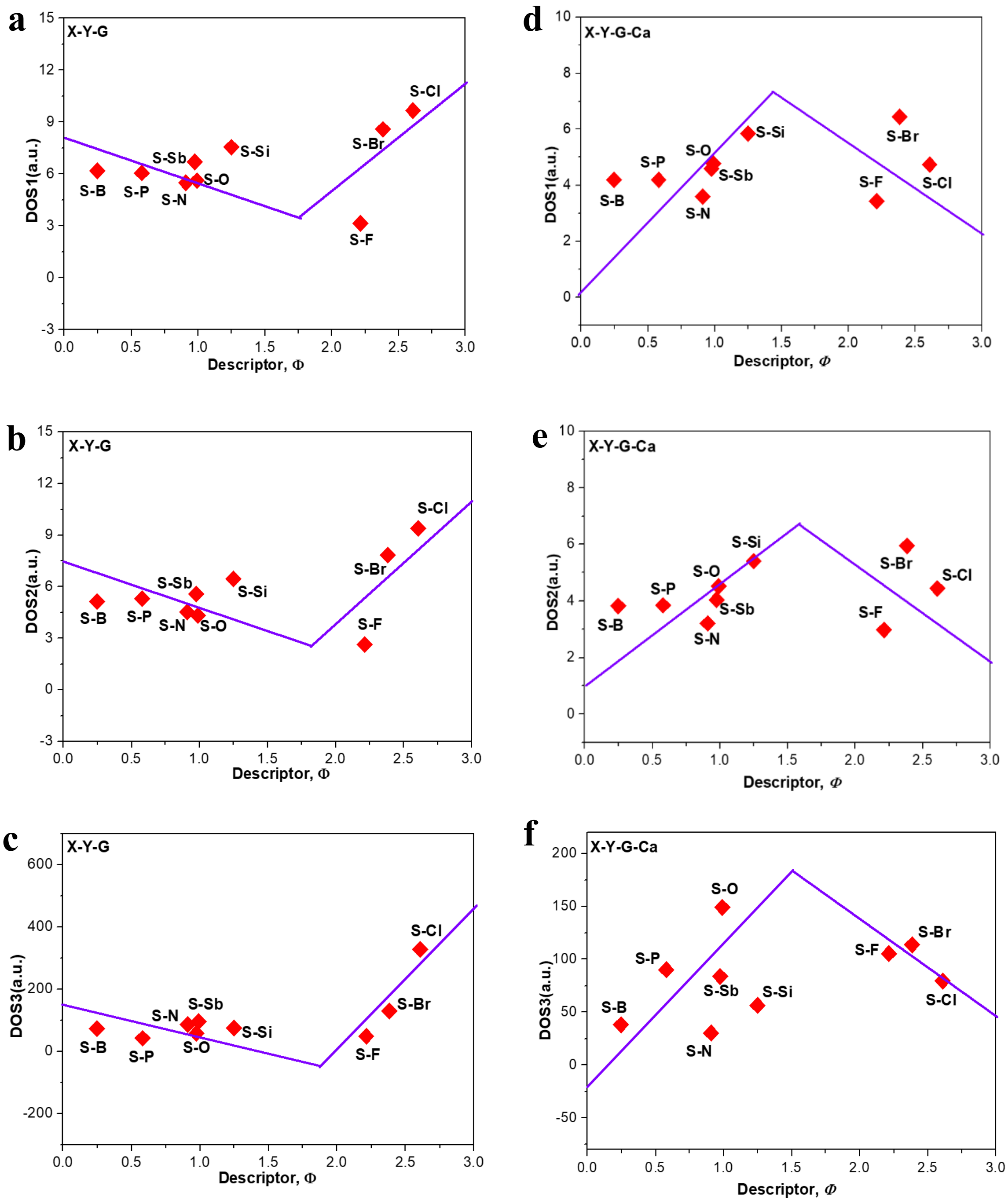

a
X-Y-G
DOS1(a.u.)
Descriptor, Φ
S-B
S-P
S-N
S-Sb
S-O
S-Si
S-Br
S-Cl
S-F
b
X-Y-G
DOS2(a.u.)
Descriptor, Φ
S-B
S-P
S-N
S-Sb
S-O
S-Si
S-Br
S-Cl
S-F
c
X-Y-G
DOS3(a.u.)
Descriptor, Φ
S-B
S-P
S-N
S-Sb
S-O
S-Si
S-Br
S-Cl
S-F
d
X-Y-G-Ca
DOS1(a.u.)
Descriptor, Φ
S-B
S-P
S-O
S-N
S-Sb
S-Si
S-Br
S-F
S-Cl
e
X-Y-G-Ca
DOS2(a.u.)
Descriptor, Φ
S-B
S-P
S-O
S-N
S-Sb
S-Si
S-Br
S-F
S-Cl
f
X-Y-G-Ca
DOS3(a.u.)
Descriptor, Φ
S-B
S-P
S-O
S-N
S-Sb
S-Si
S-F
S-Br
S-Cl

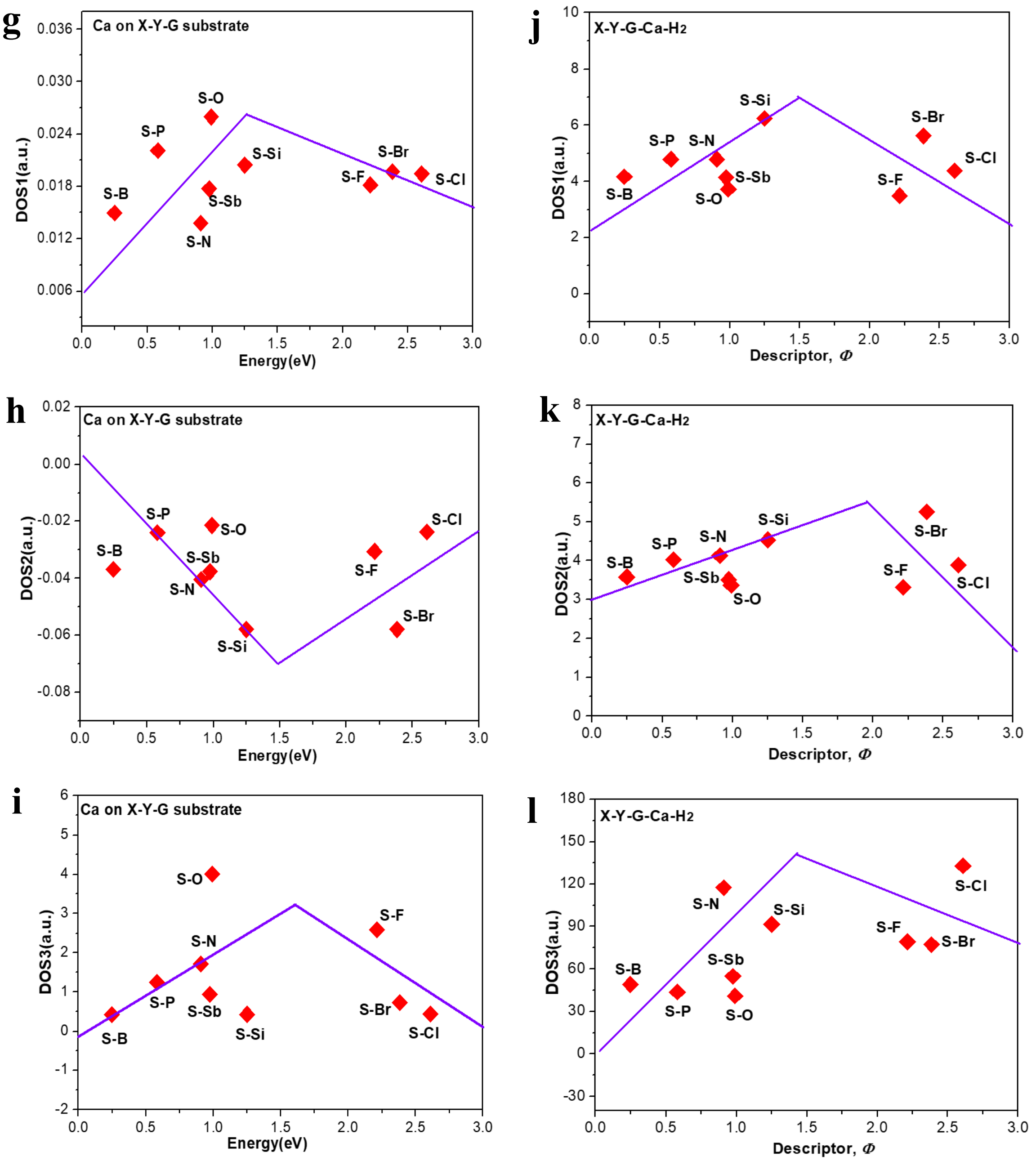

Supplementary Fig. 30 The relationships between DOS descriptors and intrinsic descriptor *Φ* for dual-doped-graphene-supported Ca single-atom solid-state H_2 storage materials. a. b. and **c.** the relationships between DOS descriptors and intrinsic descriptor *Φ* for each optimal dual-doped-graphene substrate (X-Y-G). **d. e.** and **f.** the relationships between DOS descriptors and intrinsic descriptor *Φ* for the corresponding optimal dual-doped-graphene with Ca atom chemisorption on optimal active site (X-Y-G-Ca). **g. h.** and **i.** the relationships between DOS descriptors and intrinsic descriptor *Φ* for the corresponding Ca single atom anchored optimal dual-doped-graphene substrate (Ca). **j. k.** and **l.** the relationships between DOS descriptors and intrinsic descriptor *Φ* for the corresponding optimal dual-doped-graphene-supported Ca single-atom substrate with H_2 molecule adsorbed on optimal H_2 storage site. Here, DOS descriptors consisted of DOS1, DOS2 and DOS3.

3. Supplementary Tables

Supplementary Table 1 Comparison of H_2 storage properties of various carbon-based materials

Carbon-based materials	H_2 storage condition (*T*, *P*)	H_2-uptake capacity (ρ_m & ρ_v)	Notes	Refs.
Zeolite-like carbon	77K, 2MPa	7wt.% & -	The zeolite-like carbons are prepared via chemical vapor deposition (CVD). The carbons possess surface area of up to 3200 m^2/g and pore volume of up to 2.41 cm^3/g.	4
CAs<br>Co-CA<br>Ni-CA	77K, 3MPa	5.3 wt.% & -<br>2.1 wt.% & -<br>2.3 wt.% & -	The Carbon aerogels (CAs) materials were prepared through the sol-gel polymerization of resorcinol with formaldehyde in aqueous solution to produce organic gels that are dried and subsequently pyrolyzed in an inert atmosphere.	5
N-doped microporous carbon	77K, 2MPa	6 wt.% & -	Nitrogen-doped, microporous carbon materials have been prepared using zeolite EMC-2 as a hard template and acetonitrile(C_2N_2) as the carbon source via chemical vapor deposition (CVD). The carbon products exhibited high surface areas (up to 3360 m^2/g), high pore volumes (up to 1.71 cm^3/g) and high proportion of porosity (up to 85% of surface area and 73% of pore volume).	6
Activated carbon (AC)	77K, 2MPa	7.08 wt.% & - 43.2 g H^2 L^{-1}	This kind of activated carbon with further CO_2 and KOH activations for hydrogen storage was prepared, the excellent hydrogen storage properties of which were attributed to high surface area of up to 3190 m^2 g^{-1}, more available adsorption sites, higher edge orientation, and these pores with diameter between 1 and 2 nm.	7
Ultrahigh surface area carbons	77K, 2MPa	7.03 wt.% & 37 g H^2 L^{-1}	Ultrahigh surface area carbons (3000-3500 m^2 g^{-1}) have been obtained via chemical activation of polypyrrole with KOH. The carbon materials exhibit large pore volumes (up to 2.6 cm^3 g^{-1}) and possess two pore systems: one of pores in the micropore range (1.2 nm) and the other in the small mesopore range (2.2-3.4 nm).	8
Li-doped CNTs	77K, 0.1 MPa	3.9wt.% & -	The doping of dispersed lithium (Li) onto multi-wall carbon nanotubes (CNTs) was performed by a solution method for hydrogen storage. This is an atomically dispersed Li, not Li nanoparticles.	9
CNT<br>Ca-CNTs<br>Co-CNTs<br>Fe-CNTs<br>Ni-CNTs<br>Pd-CNTs	298K,0.1MPa	0.3wt.% & -<br>1.05 wt.% & -<br>1.5 wt.% & -<br>0.75 wt.% & -<br>0.4 wt.% & -<br>7 wt.% & -	These multiwalled carbon nanotubes (MWCNTs) were decorated by Ca, Co, Fe, Ni, and Pd particles, of which hydrogen storage capacity can be improved in two approaches: (i) H_2 adsorption via Kubas interaction and (ii) dissociation of H_2 molecules on the metal particles.	10
Graphene-like nanosheets	77K, 1MPa	1.2 wt.% & -	Graphene-like nanosheets have been synthesized by the reduction of a colloidal suspension of exfoliated graphite oxide. The graphene sheets with a BET surface area of 640 m^2/g are found to be in a highly agglomerated state, with many wrinkles.	11
Hierarchical graphene	77K, 0.1MPa	4wt.% & -	The hierarchical graphene material composed of micropore (∼0.8 nm), mesopore (∼4 nm), and macropore (>50 nm) and with a specific surface area up to 1305 m^2 g^{-1}。	12
GO<br>GO-MWCNT<br>RGO-MWCNT	298K, 5 MPa	1.4 wt.% & -<br>2.6 wt.% & -<br>2.1 wt.% & -	Self-aligned GO-Multiwalled carbon nanotube (MWCNT) hybrid frameworks with a high degree of orientation. The remarkable synergistic properties from combining GO and MWCNTs in a hybrid structure offer a novel yet simple way of designing GO based hybrid frameworks with extraordinary hydrogen storage capacities.	13
Gr<br>Pt-Gr<br>Pd-Gr	303K, 5.7 MPa	0.07 wt.% &-<br>0.15 wt.% & -<br>0.16 wt.% &-	Graphene samples prepared by the exfoliation of graphitic oxide were decorated with platinum (Pt) and palladium (Pd) nano-particles by an electroless deposition technique.	14
HEG<br>Pd-HEG mixture<br>N-HEG<br>Pd-N-HEG	298K, 2MPa	0.53 wt.% & -<br>0.63 wt.% & -<br>0.88 wt.% & -<br>1.97 wt.% & -	Nitrogen doping is more effective in increasing the adsorption of hydrogen than the mechanically mixed Pd-HEG nanocomposite. It should be, however, borne that the Pd-HEG composite comprises of large sized Pd particle agglomerates. In case of the Pd-N-HEG specimen, where a more uniform dispersion has been achieved.	15
GP-NiB	77K, 0.1MPa	4.4 wt.% & -	A three-dimensional graphene material was doped with Ni-B nanoalloys via a chemical reduction process. The graphene doped with Ni (0.83 wt%) and B (1.09 wt%) shows the best hydrogen storage capacity, which is better than that of pristine graphene by three times.	16
rGO-Mg	473K,1.5MPa	6.5 wt.% & 0.105Kg H_2 L^{-1}	This is a new hydrogen storage material constructed of Mg nanocrystals encapsulated by atomically thin and gas-selective reduced graphene oxide	17

			(rGO)sheets. As rGO is atomically thin, this approach minimizes inactive mass in the composite.	
CMP	77K, 0.1MPa	1.6 wt.% & -	Conjugated microporous polymers (CMPs) exhibits a relative small hydrogen uptake value of 1.6 wt% at 1 bar. Lithium-doped conjugated microporous polymers (Li-CMP) with ionic state Li content of 0.5wt.% (optimal content) exhibits a significant maximum volume of 6.1 wt%.	18
Li-CMP		6.1 wt.% & -		
MXene	298K, 6MPa (H_2)	8.8 wt.% & -	Hydrofluoric acid incompletely etched MXene, namely, a multilayered Ti_2CT_x stack that shows an unprecedented hydrogen uptake. The hydrogen release is controllable by pressure and temperature below 95°C. The storage mechanism is deduced to be a nanopump-effect-assisted weak chemisorption in the sub-nanoscale interlayer space of the material.	19
	298K, 0.1MPa (air)	4 wt.% & -		
Oxygen-rich microporous carbons	77K, 2MPa	8.1 wt.% & 44 g H_2 L^{-1}	This study described cellulose acetate-derived carbons that combine high surface area (3800m^2g^{-1}) and pore volume (1.8 cm^3g^{-1}) that arise almost entirely (>90%) from micropores, with an oxygen-rich nature, which exhibited excellent hydrogen storage properties.	20
	77K, 3MPa	8.9 wt.% & 48 g H_2 L^{-1}		
	298K, 3MPa	1.2 wt.% & 6.5 g H_2 L^{-1}		
1 % Pd/Gr	298K, 5~6MPa	6.7~8.67 wt.% & -	This study developed a Pd-graphene nanocomposite with Pd loading amounts of 1 at.% (1% Pd/graphene) and 5 at. % (5% Pd/graphene) for hydrogen storage. The spherically shaped Pd nanoparticles of 5-45 nm in size are homogeneously distributed over the graphene matrix.	21
5 % Pd/Gr	298K, 6MPa	7.16 wt.% & -		
Ni/Gr	298K, 0.1MPa(H_2)	0.14 wt.% & -	This study has designed a Ni-graphene composite for hydrogen storage with Ni nanoparticles of 10 nm in size, uniformly dispersed over a graphene substrate. The hydrogen release could occur at an operating temperature below 150 °C and completes at 250 °C.	22
	298K, 6MPa(H_2)	1.18 wt.% & -		
Pt@GO/MOF hybrid composites	298K, 8MPa	0.77 wt.% & -	In this work, series of Pt-loaded GO/MOF hybrid composites were prepared via in-situ synthesis method, in which Pt nanoparticles were well-dispersive and anchored tightly into GO/MOF units.	23
LBMH@G	623K, 0.3MPa	8.9 wt.% & -	Flexible and 3D-structured graphene serves as the flexible structure support and MgH_2 nanoparticles homogeneously distributed on graphene serve as favorable heterogeneous nucleation sites to uniformly adsorb the solution containing $LiBH_4$ and hence, construct $LiBH_4$ around MgH_2 nanoparticles, monodisperse 2$LiBH_4$-MgH_2 nano-composite (LBMH@G) with a particle size of 10.5nm was controllably fabricated on graphene.	24
Graphene	303K, 0.1MPa	14.67 wt.% & -	A moderate, possibly scalable way involving Benkeser reaction was employed to obtain chemically hydrogenated Graphene nanosheets. Almost 15% (by weight) hydrogen was stored during hydrogenation of graphene under Benkeser reaction.	25
P-Doped Graphene	298K, 10MPa	2.2 wt.% & -	P-substituted graphene material with multi-layers of graphene was prepared through a simple and effective hydrothermal reaction under the assistance of H_3PO_4 as the phosphorous source, and showed a nearly ~2.2 wt% hydrogen sorption capacity at 298 K and 100 bar. This value is higher than that for reduced graphene oxide.	26
RGO		0.28 wt.% & -		
GO		0.20 wt.% & -		
Graphite		0.14 wt.% & -		
N-Doped graphene	298K, 9MPa	1.5 wt.% & -	The nitrogen doped graphene was synthesized by hydrothermal route utilizing 2-Chloroethylamine hydrochloride as nitrogen precursor in the presence of graphene oxide (GO). Nitrogen-doped graphene material is developed for its application in hydrogen storage at room temperature.	27
GO		0.21 wt.% & -		
Graphite Powder		0.14 wt.% & -		
Mg-Ni/GLM	623K,0.1MPa	6.5 wt.% & -	A novel method allowing to produce nickel-graphene composites containing nanosized Ni particles uniformly distributed over the graphene-like materials (GLM) surface has been developed, which are the effective catalysts of the hydrogenation of magnesium.	28
LiH@G	773K, 0.001 MPa	6.8 wt.% & -	This study reported a facile and scalable wet-chemical synthesis of LiH nanosheets with a thickness of only 2 nm, uniformly distributed on graphene. Chemical reaction of LiH@G with B_2H_6 and BH_3NH_3 synthesized $LiBH_4$ and $LiNH_2BH_3$ by adopting LiH@G as the nanoreactor. Consequently, the as-formed $LiBH_4$/graphene hybrids ($LiBH_4$@G) and $LiNH_2BH_3$/graphene hybrids ($LiNH_2BH_3$@G) exhibit a curved thin, flaky morphology, in which $LiBH_4$ with nanolayer thickness of 4nm and $LiNH_2BH_3$ nanosheets are uniformly anchored on the graphene.	29
$LiBH_4$@G	613K, 0.001 MPa	9.7 wt.% & -		
CNTs	77K(298K), 6.5 MPa	1.21 wt.% (<0.01wt.%) & -	The CNTs were grown by the CVD method using LPG and $LaNi_5$ as the carbon source and catalyst, respectively. Pd was impregnated on the CNTs by the reflux method with hydrogen gas as a reducing agent, while V was embedded on the CNTs by the vapor deposition method. The average metal particle size deposited on the CNTs was around 5.8 nm for Pd and 3.6 nm for V.	30
Pd-CNTs		0.37 wt.% (0.125 wt.%)& -		
V-CNTs		0.4 wt.% (0.1 wt.%) & -		
MWCNTs	373K, - MPa	0.15 wt.% & -		

MWCNT/h-BN		2.3 wt.% & -	The work reported the hydrogen storage performance of multi-walled carbon nanotubes (MWCNT)/hexagonal boron nitride (h-BN) nanocomposites (MWCNT/h-BN), where ultrasonication method is adopted for the synthesis of the MWCNT/h-BN nanocomposites.	31
$NaAlH_4$@GNS	504K, - MPa	- & -	A bottom-up strategy was employed to confine $NaAlH_4$ between graphene nanosheets with a millefeuille-liked multi-layer morphology, to further prepare the NaAlH4@GNS composite. The $NaAlH_4$ particles were uniformly arranged between graphene layers, with a high loading up to 90%.	32
Clay-graphene nanocomposites	298K, 20MPa	0.1 wt.% & -	This work proposed clay-graphene nanomaterials prepared by a green way using sucrose (caramel) and two types of natural clays (montmorillonite and sepiolite) as precursors to evaluate their potential use in hydrogen storage.	33
P-doped porous carbon	298K, 10MPa	1.2 wt.% & -	The synthesis of heteroatom (P)-doped porous carbon with specific surface area of 1406 m^2/g derived from the paste of newly growing Indian banyan tree (*Ficus benghalensis*) is described.	34
Mg-(Ni-TiO_2)@rGO	523K, 0.01MPa	4.3 wt.% & -	Ni and TiO_2 co-doped reduced graphene oxide [(Ni-TiO_2)@rGO] nanocomposite was synthesized and introduced into Mg via ball milling. The results demonstrated that the dispersive distribution of Ni and TiO_2 with a particle size of 20-200 nm in the reduced graphene oxide matrix led to superior catalytic effects on the hydrogen storage properties.	35
Porous graphene scaffold	193K, 1.2MPa	4 wt.% & -	This study used optimized KOH activation procedure to prepare highly porous graphene scaffold materials with SSA values up to 3400 m^2/g and pore volume up to 2.2 cm^3/g, which are among the highest for carbon materials.	36
rGO	298K, 8MPa	1.34 wt.% & -	In this paper, GO was found to exhibit better H_2 uptake capacity (1.90wt.%) as compared to rGO (1.34 wt.%) at room temperature. It can be said that the oxygen functional groups work as spacers in between the graphene layers and increase the inter-layer space which in turn accumulate more number of hydrogen molecules on surface of carbon nano-sheets.	37
GO		1.90 wt.% & -		
GO	77K(298K), 9MPa	4.8(0.5) wt.% & -	The maximum storage capacity is 4.8 (0.5) wt% at 77 K (298 K) and at 9.0 MPa pressure, and the optimum GO interlayer distance for maximum H_2 uptake is 6.5Å, similar to the predicted distances from first-principles calculations for graphite materials.	38
Mg	373K, 7MPa	0.0 wt.% & -	Reduced graphene-oxide-supported nickel (Ni@rGO) nanocomposite catalysts were synthesized, and incorporated into magnesium (Mg) hydrogen storage materials with the aim of improving the hydrogen storage properties of these materials. The experimental results revealed that the catalytic effect of the Ni@rGO nanocomposite on Mg was more effective than that of single nickel (Ni) nanoparticles or graphene.	39
Ni@rGO		0.0 wt.% & -		
Mg-Ni		4.7 wt.% & -		
Mg-Ni@rGO		5.48wt.% & -		
Gr	-	2wt.% & -	In this work, a Ca/graphene nanocomposite was successfully synthesized. The XRF characterization results proved that the Ca was successfully loaded on the graphene matrix with the Ca percentage moving from 0.516% in graphite flakes to 8.227% in Ca/graphene composite reduced by ammonia.	40
Ca/Gr reduced with ammonia	-	4.98 wt.% & -		
Ca/Gr reduced with L-Ascorbic acid	-	3.99wt.% & -		
Ni/Al/graphene	473K,	5.7wt.% & -	Dual active metals Ni and Al doped graphene composites are prepared through in-suit reaction and self-assembly with high-temperature reduction process. The maximum hydrogen storage uptake of such composites is up to 5.7 wt% at 473 K, and the dehydriding efficiency is high as 96%~97% at the dehydriding temperature of 380 K.	41
Li-doped CNT	653K,0.1MPa	20wt.% & 160 kg/m^3	Lithium- or potassium-doped carbon nanotubes can absorb 20 or 14 weight percent of hydrogen at moderate (200°C to 400°C) or room temperatures, respectively, under ambient pressure. The hydrogen stored in the lithium or potassium-doped carbon nanotubes can be released at higher temperatures, and the sorption-desorption cycle can be repeated with little decrease in the sorption capacity.	42
K-doped CNT	298K,0.1MPa	14wt.% & -112 kg/m^3		
GR-supported MgH_2 NPs	473K, 3MPa	5.7wt.% & -	GR-supported MgH_2 NPs were first fabricated by the hydrogenation of dibutyl magnesium ((C_4H_9)$_2$ Mg) in cyclohexane (C_6H_{12}) under the structure-directing role of GR.	43
Li-doped fulleranes	823K, -	5wt.% & -	This work synthesized polymeric lithium and sodium doped fulleranes (6:1 mol ratio, A_6C_{60}) which can reversibly store hydrogen via a chemisorption mechanism through the formation of C–H bonds on the fullerene owing to the presence of the alkali metal.	44
Na-doped fulleranes	823K, -	4wt.% & -		
Ti-covered graphene	400K-800K, -	-&-	This study reports on hydrogen adsorption and desorption on titanium-covered graphene in order to test theoretical proposals to use of graphene functionalized with metal atoms for hydrogen storage. At room temperature, titanium islands grow on graphene with an average diameter of about 10 nm.	45
Nanoporous materials	77K, -	10wt.% & 40g H_2 L^{-1}	In materials with particularly high BET areas, such as some MOFs, gravimetric storage capacities exceeding 10 wt% can be achieved, although only at cryogenic temperatures (77 K). The volumetric capacity achieved	46

			using either powder compaction, monolith formation or framework interpenetration can exceed 40 g H_2 L^{-1}, but again only at 77 K.	
Bulk graphene	298K, 12MPa	1wt.% & -	It can be concluded that bulk graphene-related materials do not exhibit superior hydrogen storage parameters compared to other nanostructured carbon materials like activated carbons, carbon nanotubes or nanofibers.	47
GNR-Mg nanocomposite	498K, 1.5MPa	7.1wt.% & -	This paper reports Mg nanocrystals encapsulated by narrow, bottom-up synthesized graphene nanoribbons (GNRs) as environmentally stable and high-capacity hydrogen storage materials. As an encapsulation medium, GNRs offer similar functionalities as reduced graphene oxide to protect the encapsulated Mg nanocrystals from extensive oxidation, while allowing penetrations of hydrogen.	48
Mg-GNP composites	573K, 2MPa	-&-	This work shows that loading magnesium by graphene nanoplatelets (GNP) enhances the kinetics by more than an order of magnitude. The GNP presence reduces the Mg agglomeration, induced by de/hydriding, and accelerates the kinetics by connecting between Mg particles.	49
Multivalent VO_2 epitaxial thin films	-	-	This study demonstrates that as many as two hydrogen atoms can be incorporated into each VO_2 unit cell, and that hydrogen is reversibly absorbed into, and released from VO_2 without destroying its lattice framework.	50

Supplementary Table 2 The definition of adsorption energy, maximum adsorption energy per H_2 (E_{ad}^{max} in unit of eV/H_2), theoretical maximum reversible hydrogen storage capacity (*C*), descriptor (*Φ*) corelating microstructure to hydrogen storage performance for current various carbon-based materials based on theoretical calculation investigation.

Materials	Simulation methods	Simulation software package	Hydrogen storage mechanisms	Definition of adsorption energy	E_{ad}^{max}	*C*	*Φ*	Refs.
Ni-Pd-Co metals decorated B-doped graphene	PAW-GGA-PW91	DFT@VASP	Hydrogen (H_2) adsorption and hydrogen atom (H) spillover	$E_{ad} = E_{Total} - E_{surface} - nE_{H_2}$ (39)	-2.410	-	-	51
Al-doped graphene	PAW-LDA	DFT @DMol & *ab initio* MD@CASTEP	Hydrogen (H_2) physisorption enhanced by doping Al into graphene	$E_{ad} = E_{graphene} - E_{surface} - nE_{H_2}$ (40)	-0.260	5.31wt.%	-	52
Al-adsorbed graphene	PAW-LDA	DFT @ DMol & *ab initio* MD	Hydrogen (H_2) physisorption by adsorbing Al into graphene	$E_{b-H_2} = [E_{iH_2+Al-graphene} - (E_{Al-graphene} + iE_{H_2})]/i$ (41)	-0.193	13.8wt.%	-	53
Ca-adsorbed graphene	PAW-GGA-PBE	DFT@VASP	Hydrogen (H_2) adsorption by Kubas effect and polarization	-	-0.200	5 wt.%	-	54
Ca-adsorbed CNTs	PAW-GGA-PBE	DFT@VASP	Hydrogen (H_2) adsorption by Kubas effect	-	-0.200	5 wt.%	-	55
Light-element-doped fullerenes	USPP-LDA	DFT@VASP & QMC@ CASINO	Non-dissociative adsorption of H_2 molecules	$E_a = E_{far} - E_{bond}$ (42)	0.650	1.7 wt.%	-	56
Li-adsorbed porous graphene	PAW-LDA	DFT@VASP	Polarization mechanism	$E_b = E(graphene + Li + H_2) - E(graphene + Li) - E(H_2)$ (43)	-0.265	12 wt.%	-	57
N-doped penta-graphene	PAW-GGA-PBE	DFT@ DMOL in MS	Stronger adsorption induced by negatively charged N-doped penta-Graphene	$E_{ads} = E_{total} - (E_{N-PG} + E_{gas})$ (44)	-0.032	2.7 wt.%	-	58
Li-adsorbed graphene	PAW-GGA-PBE	DFT@VASP	Each Li atom absorbing H_2 molecules by polarizing them	$\bar{E}_i = [E(Li/graphene + iH_2) - E(Li/graphene) - iE(H_2)]/i$ (45)	-0.170	16 wt.%	-	59
Al-adsorbed porous graphene	LDA with PWC	DFT@ DMOL	Each Al atom absorbing H_2 molecules by polarizing them	$E_{ad-H_2} = E_{iH_2+Al-graphene} - (E_{(i-1)H_2+Al-graphene} + E_{H_2})$ (46)	-1.110	10.5wt.%	-	60
Pillared graphene	PAW-GGA-PBE	DFT&GCMC	Adsorbing H_2 by charge induced dipole	-	-0.014	40g H_2/L	-	61
Ca-adsorbed porous graphene	GGA	DFT@VASP	Adsorption	-	-0.23	9 wt.%	-	62
CNTs	-	MD & VASP	Chemisorption	-	-	7.7 wt.%	-	63
Ti-anchored GO	PAW-GGA-PBE & PAW-LDA	DFT@VASP	Kubas effect	$E_b = E[Ti/HGO(H_2)_{n-1}] + E(H_2) - E[Ti/HGO(H_2)_n$ (47)	-0.410	4.9 wt.% & 64g H_2/L	-	64
Ca-adsorbed Graphene	-	DFT@VASP	Ionic bonding and weak van der Waals interaction	-	-0.400	8.4 wt.%	-	65
Transition metal (TM)	USPP-GGA & PAW-GGA-	DFT@VASP	Kubas effect	-	-0.300	9.0 wt.%	-	66

atoms bound to fullerenes	PBE							
Pd-adsorbed graphene	PAW-LDA	DFT@ DMOL	Polarization mechanism and orbital hybridization	$E_{av} = -[E_{Pd(H_2)_n} - E_{Pd} - nE_{H_2})]/n$ (48)	-1.315	3.6 wt.%	-	67
Pt_4 cluster supported on pristine, B-, and N-doped graphene sheets	PAW-GGA-PBE	DFT@VASP	Co-existed dissociative and molecular adsorption of multiple H_2 molecules	$E_{ads} = E[nH_2 - substrate] - E[(n-1)H_2 - substrate] - E[H_2]$ (49)	-1.540	-	-	68
Ni-adsorbed graphene	PAW-GGA-PBE	DFT@ SIESTA	Adsorption	$\Delta E_{ad-H_2} = [E_{Ni,G}(n_{H_2}, 1) - E_{Ni,G}(0,1) - nE_{H_2}(\infty)]/n_{H_2}$ (50)	-1.210	2.8 wt.%	-	69
Cu and Pd-decorated graphene	PAW-GGA-PBE	DFT@QE	Adsorption	$\Delta E_b(H_2) = [E_{Graphene+Metal\ atom+Hydrogen} - E_{Graphene+Metal\ atom} + E_{Hydrogen\ molecule}]$ (51)	-0.960	-	-	70
Sc-decorated graphene with pyridinic-N defects	PAW-GGA-PBE	DFT@VASP	Adsorption	$E_b = -(E_{M-G} + nE_{H_2} - E_{M-G+nH_2})/n$ (52)	-0.400	2.8 wt.%	-	71
Ti-decorated *cis*-polyacetyl-ene	PAW-GGA-PBE	-	Adsorption	-	-	7.6 wt.% & 63 kg/m^3	-	72
Pd-decorated B-doped defective graphene	PAW-GGA-PBE	DFT@VASP	Co-existed van der Waals adsorption, hybridization, and electrostatic interaction	$E_{ads} = E[Pd + G + nH_2] - E[Pd + G + (n-1)H_2] - E[H_2]$ (53)	-0.410	-	-	73
Mg-decorated graphene	PAW-GGA-PBE	DFT@ ESPRESSO	Weak van der Waals adsorption and electrostatic interaction	$E_{net-ads(H_2)} = E_{total-ads(H_2)} - E_{int(H_2)}$ (54)	-0.134	-	-	74
N- and B-doped penta-graphene	PAW-GGA-PBE	DFT@VASP	Co-existed dissociative and molecular adsorption of H_2 molecules	$E = E_{tot} - (E_{sub} + E_{H_2})$ for H_2 $E = E_{tot} - (E_{sub} + 1/2E_{H_2})$ for H (55)	-0.016	-	-	75
Li-decorated graphene	GGA-PBE+G+S+D	DFT@ CASTEP code in MS	Stroger chemisorption	$E_{ad} = \frac{E_{nH_2+LiGR} - E_{LiGR} - nE_{H_2}}{n}$ (56)	-0.263	-	-	76
Li-decorated graphene with defects	GGA-PBE +D	DFT @DMol code in MS	Polarization interaction	$E_{ad} = \frac{E_{nH_2+LiGR} - E_{LiGR} - nE_{H_2}}{n}$ (57)	-0.460	-	-	77
V-decorated porous graphene	GGA-PBE +D	DFT@ CASTEP code in MS	Attractive Coulomb interaction	$\overline{E_{ad}} = [E_{nH_2+V+PG} - E_{V+PG} - nE(H_2)]/n$ (58)	-0.791	4.6 wt.%	-	78
Defective graphene	GGA-PBE & vdW-DF2	DFT@QE	Adsorption	$E_b = \frac{E_{system+iH_2} - E_{system} - iE_{H_2}}{i}$ (59)	-0.073	7.0 wt.%	-	79
Ca-decorated graphene	GGA-PBE & GGA-LDA & vdW-DF	DFT@VASP	Adsorption	-	-0.062	2.6 wt.%	-	80
Sc and Ti Containing Single-Walled	GGA-PBE+D	DFT @DMol	Kubas effect	$E_{ads} = E_{Sc/4ND-CNxNT+nH_2} - E_{Sc/4ND-CNxNT} - E_{nH_2}$ (60)	-0.608	5.9 wt.%	-	81

Carbon Nanotubes				$E_{ads} = E_{Ti/4ND-CNxNT+nH_2} - E_{Ti/4ND-CNxNT} - E_{nH_2}$ (61)				
Ca-decorated boron-doped graphene	GGA-LDA & GGA-PBE	DFT@ SIESTA	Polarization and hybridization	-	-0.400	8.4 wt.%	-	82
Graphene with Boron Substitution	GGA-PBE	DFT @DMol	Chemisorption via H spillover	$\Delta E_H = E_{Sub-H} - E_{sub} - E_H$ (62)	-1.933	-	-	83
Defective graphene	USPP-LDA	DFT@QE & MD	Adsorption	-	-	-	-	84
Pt-adsorbed graphene	GGA-PBE	DFT@ CASTEP code in MS	Adsorption	$E_{ad} = E_{(g+H_2)} - E_g - E_{H_2}$ (63)	-0.083	8.4 wt.%	-	85
Li-adsorbed graphene	USPP-LDA	DFT@VASP	Weak ionic bond, attractive Coulomb interaction and weak van der Waals interaction	-	-0.410	12.8 wt.%	-	86
Li, Na and Mg-decorated BC_3 graphene	GGA-LDA	DFT @DMol	Hybridizations	$E_{ad} = \frac{E_{nH_2/M/BC3} - E_{M/BC3} - nE_{H_2}}{n}$ (64)	-0.310	-	-	87
TM-doped defected graphene	PAW-GGA-PBE	DFT@VASP	Adsorption of molecular hydrogen	-	-0.870	5.1 wt.%	-	88
Si-doped SWCNTs and graphene	GGA-LDA	DFT @DMol & GCMC@DREIDING	Physisorption	$E(adsorption) = E(hostmaterial - H_2) - E(hostmaterial) - E(H_2)$ (65)	-0.190	2.5 wt.%	-	89
Ti-embedded graphene	GGA-PBE	DFT@ ESPRESSO	Molecular chemisorption between physisorption and chemisorption	-	-0.310	6.3 wt.%	-	90
Bare Cu atom and Cu-functionalized boron-doped graphene	PAW-GGA-LDA	DFT @DMol in MS	Adsorption in molecular form	$E_{ad} = \frac{E_{G+M+iH_2} - E_{G+M} - iE_{H_2}}{i}$ (66)	-1.352	4.2 wt.%	-	91
Si-decorated graphene	LDA-CA	DFT @SIESTA	Adsorption in molecular form	$E_b(H_2) = \frac{E_{mH_2/Si-graph} - E_{Si-graph} - mE_{H_2}}{m}$ (67)	-0.190	15 wt.%	-	92
Ca atom- and Ca dimers-decorated graphene	PAW-GGA-LDA	DFT@VASP	Hydrogen spillover storage	$E_b = \frac{E_T - E_0 - nE_{H_2}}{n}$ (68)	-1.020	7.7 wt.%	-	93
B-doped Graphene	GGA-PBE+D	DFT @DMol	Adsorption in molecular form	$E_{ads} = -\frac{E_{adsorbent} - E_{adsorbent-H_2} + nE_{H_2}}{n}$ (69)	-0.060	5.3wt.%	-	94
Li-adsorbed graphene	-	DFT@ CAM-B3LYP/6-311G (d, p)	Adsorption in molecular form	$-E_{bind}(n, H_2) = -\frac{E(GR - Li - (H_2)_n) - E(GR - Li) + nE_{H_2}}{n}$ (70)	-0.170	-	-	95
Li-adsorbed B-doped Graphene	GGA-PBE	DFT @DMol	Polarization	-	-0.130	-	-	96
Graphene	GGA-PBE	DFT@ Gaussian	Spillover mechanism	$\varepsilon_b(n) = (E_{nH@g} - E_g - n\varepsilon_H)/n$ (71)	-2.350	-	-	97
Li-doped fluorinated graphene	GGA-PBE	DFT @DMol	Adsorption from hybridization between Li and graphene	$\Delta E_n = E[F + Li + graphene + (H_2)_n] - E[F + Li + graphene + (H_2)_{n-1}] - E[H_2]$ (72)	-0.266	16.2 wt.%	-	98
Li-doped Graphene and nanotubes	USPP-LDA	DFT@ DACAPO	Physisorption	$E_b = E(Li + graphene + H_2) - E(graphene + H_2) - E(H_2)$ (73)	-0.180	-	-	99

Li-doped carbon nanotube	PAW-GGA-LDA	DFT@ Real-space code	Adsorption in molecular form	$\mu = E(System + H_2) - E(System) - E(H_2)$ (74)	-1.090	3.5 wt.%	-	100
Cs-doped C_{60}	ωB97X-D	DFT@ Gaussian	Physisorption	$D_e(m) = E((H_2)_m C_{60} Cs^+) - E((H_2)_{m-1} C_{60} Cs^+) - E(H_2)$ (75)	-0.051	8.4 wt.%	-	101
Ca-decorated graphdiyne	GGA-PBE+D2	DFT@ ESPRESSO	Adsorption in molecular form	$\bar{E}_{ads} = (E_{nH_2-Ca-GDY} - E_{Ca-GDY} - nE_{nH_2})/n$ (76)	-0.266	18 wt.%	-	102
K-decorated graphdiyne	B3LYP/6-311G*	DFT@ Gaussian& MD@QNIOM	Physisorption in molecular form	-	-0.148	-	-	103
Corrugated graphene multilayer system	GGA-PBE	DFT&MD@CPMD	Physisorption in molecular form	-	-2.000	8.0 wt.%	-	104
g-C_4N_3 and g-C_3N_4	GGA-PBE	DFT @DMol	Physisorption in molecular form	$E_{ads}(n) = E_{adsorbent-nH_2}/n - E_{adsorbent} - nH_2$ (77)	-0.250	7.1 wt.%	-	105

Supplementary Table 3 The comparison between current established H_2 storage theories and generalized Sabatier principle proposed in this work for adsorption-type hydrogen storage materials.

	Basic thermodynamic theory	Refs.
**Physical picture of H_2 storage**	-	
**Universal theoretical equations**	$\Delta\mu = \Delta h - T\Delta S$ (78)<br>where $\Delta\mu$, Δh , ΔS and T are chemical potential change, enthalpy change and entropy change of hydrogen in free and adsorbed states and temperature, respectively.	106
**Comments**	This is a simple analysis based on the fact that the difference in chemical potential of hydrogen in free and adsorbed states $\Delta\mu = \Delta h - T\Delta s$ controls the direction of adsorption/desorption. However, it cannot express hydrogen storage capacity and rate directly. More significantly, this theory is not able to establish the relationship between hydrogen storage performance and hydrogen storage materials, which leads to inferior guidance for design of hydrogen storage materials.	
	**Statistical physics theory in equilibrium state**	
**Physical picture of H_2 storage**	-	
**Universal theoretical equations**	$f = kT\partial lnZ/\partial\mu$ (79)<br>$f = \frac{\sum_{l=0} l g_l e^{l(\mu-\varepsilon_l)/kT}}{\sum_{l=0} g_l e^{l(\mu-\varepsilon_l)/kT}}$ (80)<br>where Z is the grand partition function, μ is the chemical potential of H_2 in the gas phase at given pressure p and temperature T, k is the Boltzmann constant, and f is the occupation (adsorption) number of H_2 molecules per site.	72
**Comments**	This theory is built in equilibrium state of H_2 adsorption/desorption without external driving condition. In reality, both hydrogen storage and release are driven by external condition such as temperature T and pressure P. In addition, it cannot express hydrogen storage rate directly. More significantly, this theory is not able to establish the relationship between hydrogen storage performance and microstructure of hydrogen storage materials, which leads to inferior guidance for design of hydrogen storage materials as mentioned above.	

	Generalized design principle	

Physical picture of H_2 storage

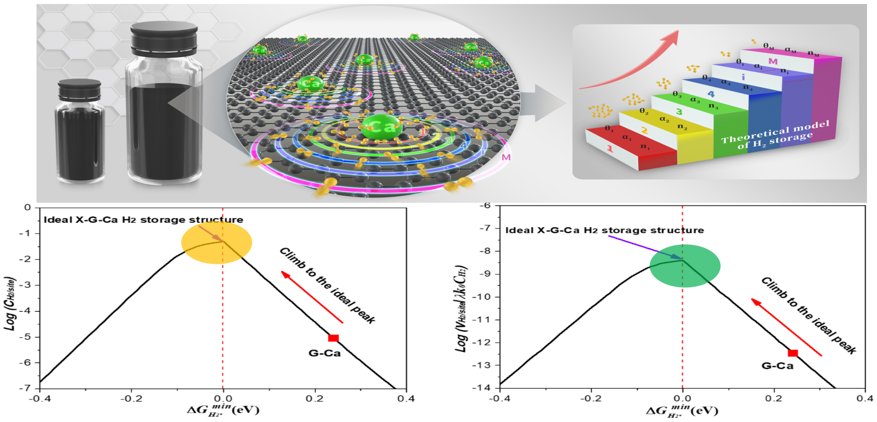

Universal theoretical equations

$$C_{H_2/site} = \begin{cases} \dfrac{-k_BT\left(e^{-\frac{\Delta G_{H_2^*}^{max}}{k_BT}} - e^{-\frac{\Delta G_{H_2^*}^{min}}{k_BT}}\right)}{\left(\Delta G_{H_2^*}^{max} - \Delta G_{H_2^*}^{min}\right) - k_BT\left(e^{-\frac{\Delta G_{H_2^*}^{max}}{k_BT}} - e^{-\frac{\Delta G_{H_2^*}^{min}}{k_BT}}\right)}, & \Delta G_{H_2^*}^{min} > 0 \\ \dfrac{k_BT\left(1 - e^{-\frac{\Delta G_{H_2^*}^{max}}{k_BT}}\right)}{\left(\Delta G_{H_2^*}^{max} - \Delta G_{H_2^*}^{min}\right) - k_BT\left(e^{-\frac{\Delta G_{H_2^*}^{max}}{k_BT}} - e^{-\frac{\Delta G_{H_2^*}^{min}}{k_BT}}\right)}, & \Delta G_{H_2^*}^{min} < 0 \end{cases} \quad (81)$$

$$v_{H_2/site/s} = \begin{cases} \dfrac{-\lambda k_0 c_{H_2} k_BT\left(e^{-\frac{\Delta G_{H_2^*}^{max}}{k_BT}} - e^{-\frac{\Delta G_{H_2^*}^{min}}{k_BT}}\right)}{\left(\Delta G_{H_2^*}^{max} - \Delta G_{H_2^*}^{min}\right) + k_BT\left(e^{\frac{\Delta G_{H_2^*}^{max}}{k_BT}} - e^{\frac{\Delta G_{H_2^*}^{min}}{k_BT}}\right)}, & \Delta G_{H_2^*}^{i} > 0 \\ \dfrac{\lambda k_0 c_{H_2} k_BT\left(1 - e^{-\frac{\Delta G_{H_2^*}^{max}}{k_BT}}\right)\left[\Delta G_{H_2^*}^{max} - k_BT\left(e^{-\frac{\Delta G_{H_2^*}^{max}}{k_BT}} - 1\right)\right]}{\left[\left(\Delta G_{H_2^*}^{max} - \Delta G_{H_2^*}^{min}\right) - k_BT\left(e^{-\frac{\Delta G_{H_2^*}^{max}}{k_BT}} - e^{-\frac{\Delta G_{H_2^*}^{min}}{k_BT}}\right)\right]\left[\Delta G_{H_2^*}^{max} + k_BT\left(e^{\frac{\Delta G_{H_2^*}^{max}}{k_BT}} - 1\right)\right]}, & \Delta G_{H_2^*}^{i} < 0 \end{cases} \quad (82)$$

$$\emptyset = \frac{E_X A_X R_X / N_X}{E_C A_C R_C / N_C}$$

This work

Comments

This theory is built based on actual situation of H_2 adsorption/desorption under external conditions such as temperature *T* and pressure *P*. In addition, it can give the accurate equations of hydrogen storage capacity and rate directly. More significantly, this theory is able to establish the relationship between hydrogen storage performance and microstructure of hydrogen storage materials, which results into the superior guidance for design of hydrogen storage materials. It is the most complete theory for the design of hydrogen storage materials at present, and can realize the bottom-up design from electronic structure of hydrogen storage materials to hydrogen storage performance.

Supplementary Table 4 Electron affinity (E_X, unit eV), electronegativity (A_X), atomic radius (R_X, unit Å) and number of outermost electrons (N_X) of dopants, descriptor (Φ), the most negative adsorption energy change (ΔE, unit eV) at ground state and corresponding Gibbs free energy change (ΔG, unit eV) at ambient condition (298K, 0.1MPa) upon adsorbing a H_2 molecule on optimal active site of selected optimal doped-graphene-supported Ca single atom structure.

Dopants	E_X (eV)	A_X	R_X (Å)	N_X	$\emptyset = \frac{E_X A_X R_X / N_X}{E_C A_C R_C / N_C}$	ΔE (eV)	ΔG(eV)	Optimal doped-graphene-supported Ca single atom structures with H_2
C	1.2621	2.55	0.863	4	1	-0.1127	0.2213	z-C-Ca (-H_2)
B	0.2797	2.04	0.905	3	0.2479	-0.1416	0.1924	B=C-Ca (-H_2)
P	0.7466	2.19	1.233	5	0.5807	-0.1436	0.1904	P-3C-Ca (-H_2)
N	1.4000	3.04	0.741	5	0.9084	-0.1324	0.2016	py-N-Ca (-H_2)
Sb	1.0474	2.05	1.577	5	0.9753	-0.1271	0.2069	py-Sb-Ca (-H_2)
O	1.4610	3.44	0.82	6	0.9892	-0.2160	0.118	py-O-Ca-H_2)
Si	1.3895	1.90	1.312	4	1.2471	-0.1512	0.1828	a-Si-Ca (-H_2)
S	2.0771	2.58	1.164	6	1.4972	-0.0959	0.2381	th-S-Ca (-H_2)
F	3.4011	3.98	0.794	7	2.2113	-0.1146	0.2194	z-F-Ca (-H_2)
Br	3.3636	2.96	1.164	7	2.3843	-0.1511	0.1829	a-Br-Ca (-H_2)
I	3.0590	2.66	1.487	7	2.4894	-0.1501	0.1839	a-I-Ca (-H_2)
Cl	3.6127	3.16	1.111	7	2.6095	-0.1797	0.1543	a-Cl-Ca (-H_2)
S-B	0.2797	2.04	0.905	3	0.2479	-0.1943	0.1397	S-B-Ca (-H_2)
S-P	0.7466	2.19	1.233	5	0.5807	-0.1877	0.1463	S-P-Ca (-H_2)
S-N	1.4000	3.04	0.741	5	0.9084	-0.16923	0.16477	S-N-Ca (-H_2)
S-Sb	1.0474	2.05	1.577	5	0.9753	-0.1983	0.1357	S-Sb-Ca (-H_2)
S-O	1.4610	3.44	0.82	6	0.9892	-0.16783	0.16617	S-O-Ca (-H_2)
S-Si	1.3895	1.90	1.312	4	1.2471	-0.2500	0.084	S-Si-Ca (-H_2)
S-F	3.4011	3.98	0.794	7	2.2113	-0.1937	0.1403	S-F-Ca (-H_2)
S-Br	3.3636	2.96	1.164	7	2.3843	-0.1416	0.1924	S-Br-Ca (-H_2)
S-I	3.0590	2.66	1.487	7	2.4894	-	-	-
S-Cl	3.6127	3.16	1.111	7	2.6095	-0.1471	0.1869	S-Cl-Ca (-H_2)

Supplementary Table 5 Summary of physicochemical characterization, hydrogen storage capacity measured experimentally at 77K and 5MPa, and calculation results on various doped-graphene-supported Ca single atoms non-dissociative chemisorption hydrogen storage materials (NCHSMs).

Sample	Surface area (m^2 g^{-1})	Dopant concentration (at %)	Ca loading content from XPS (at%)	C_m (wt%)	$C_{0/site}^*$ (H_2 $site^{-1}$)	ΔG_{min} (eV)
O-G-Ca	395	13.11	0.43	1.51	0.002594754	0.118
N-G-Ca	325	7.61	0.33	1.33	0.002142234	0.202
P-G-Ca	58	2.41	0.40	0.21	0.002163223	0.190

Notes: C_m represents the specific capacity for H_2 storage measured in automatic high pressure gas sorption instrument. $C_{0/site}^*$ refers to specific capacity per unit H_2 molecule storage site after normalization (See normalization process in Note 1).

Supplementary Table 6 Summary of calculated Bader charge transfer, bond length ($d_{H\text{-}H}$) and bond length change ($\Delta d_{H\text{-}H}$) of hydrogen molecule (H_2) upon H_2 molecule adsorbed on optimal active sites of various doped-graphene-supported single atom Ca models.

Dopants	$\emptyset = \frac{E_X A_X R_X / N_X}{E_C A_C R_C / N_C}$	Charge transferred to substrate from Ca (e)	Charge transferred to H_2 from Ca (e)	$d_{H\text{-}H}$(Å)	$\Delta d_{H\text{-}H}$ (Å)	Optimal doped-graphene-supported Ca single atom structures with H_2
C	1	1.1931	0.0358	0.76682	0.02582	z-C-Ca-H_2
B	0.2479	1.3859	0.0253	0.76832	0.02732	B=C-Ca-H_2
P	0.5807	1.3163	0.0022	0.76052	0.01952	P-3C-Ca-H_2
N	0.9084	0.9924	0.0220	0.75806	0.01706	py-N-Ca-H_2
Sb	0.9753	1.3417	-0.0072	0.75780	0.0168	py-Sb-Ca-H_2
O	0.9892	1.3680	0.0410	0.77091	0.02991	py-O-Ca-H_2
Si	1.2471	1.3379	0.0490	0.77134	0.03034	a-Si-Ca-H_2
S	1.4972	1.2220	0.0035	0.75737	0.01637	th-S-Ca-H_2
F	2.2113	1.1973	0.0295	0.76493	0.02393	z-F-Ca-H_2
Br	2.3843	1.3672	0.0134	0.76425	0.02325	a-Br-Ca-H_2
I	2.4894	1.3616	0.0161	0.76438	0.02338	a-I-Ca-H_2
Cl	2.6095	1.3646	0.0447	0.7683	0.0273	a-Cl-Ca-H_2
S-B	0.2479	1.2721	0.0451	0.76982	0.02882	S-B-Ca-H_2
S-P	0.5807	1.3889	0.0501	0.77447	0.03347	S-P-Ca-H_2
S-N	0.9084	1.3492	0.0399	0.76661	0.02561	S-N-Ca-H_2
S-Sb	0.9753	1.3488	0.0725	0.79305	0.05205	S-Sb-Ca-H_2
S-O	0.9892	1.4398	-0.0019	0.76119	0.02019	S-O-Ca-H_2
S-Si	1.2471	1.4164	0.0008	0.75185	0.01085	S-Si-Ca-H_2
S-F	2.2113	1.3224	0.0522	0.77466	0.03366	S-F-Ca-H_2
S-Br	2.3843	1.1696	0.0003	0.75887	0.01787	S-Br-Ca-H_2
S-I	2.4894	-	-	-	-	-
S-Cl	2.6095	1.3327	0.0238	0.76602	0.02502	S-Cl-Ca-H_2

Supplementary Table 7 Summary of calculated density of states (DOSs) including DOS1, DOS2 and DOS3 as defined above of single atom Ca chemisorbed on optimal doped-graphene substrates.

Dopants	$\emptyset = \frac{E_X A_X R_X / N_X}{E_C A_C R_C / N_C}$	DOS1	DOS2	DOS3	Ca chemisorbed on optimal doped-graphene substrates
C	1	0.0144	-0.0233	0.6499	z-C(-Ca)
B	0.2479	0.0193	-0.0311	0.9400	B=C(-Ca)
P	0.5807	0.0195	-0.0433	0.6430	P-3C(-Ca)
N	0.9084	0.0120	-0.0389	1.2824	py-N(-Ca)
Sb	0.9753	0.0202	-0.0175	1.4421	py-Sb(-Ca)
O	0.9892	0.0166	-0.0500	1.8044	py-O(-Ca)
Si	1.2471	0.0178	-0.0324	1.2547	a-Si(-Ca)
S	1.4972	0.0165	-0.0547	0.9325	th-S(-Ca)
F	2.2113	0.0112	-0.0291	6.1190	z-F(-Ca)
Br	2.3843	0.0237	-0.0174	0.7450	a-Br(-Ca)
I	2.4894	0.0212	-0.0162	1.4559	a-I(-Ca)
Cl	2.6095	0.0234	-0.0052	0.7031	a-Cl(-Ca)
S-B	0.2479	0.0149	-0.0369	0.4124	S-B(-Ca)
S-P	0.5807	0.0221	-0.0240	1.2408	S-P(-Ca)
S-N	0.9084	0.0138	-0.0404	1.7081	S-N(-Ca)
S-Sb	0.9753	0.0177	-0.0376	0.9288	S-Sb(-Ca)
S-O	0.9892	0.0260	-0.0214	4.0061	S-O(-Ca)
S-Si	1.2471	0.0204	-0.0579	0.4276	S-Si(-Ca)
S-F	2.2113	0.0182	-0.0305	2.5793	S-F(-Ca)
S-Br	2.3843	0.0198	-0.0580	0.7245	S-Br(-Ca)
S-I	2.4894	-	-	-	-
S-Cl	2.6095	0.0194	-0.0238	0.4381	S-Cl(-Ca)

Supplementary Table 8 Summary of calculated density of states (DOSs) including DOS1, DOS2 and DOS3 as defined above of optimal doped-graphene substrates.

Dopants	$\emptyset = \frac{E_X A_X R_X / N_X}{E_C A_C R_C / N_C}$	DOS1	DOS2	DOS3	Optimal doped-graphene substrates
C	1	8.6167	7.6763	63.1807	z-C
B	0.2479	8.1234	7.2536	66.0034	B=C
P	0.5807	8.0670	6.9374	28.0203	P-3C
N	0.9084	8.2645	7.4694	59.8546	py-N
Sb	0.9753	7.4815	6.5879	47.2730	py-Sb
O	0.9892	8.0967	7.3582	148.6560	py-O
Si	1.2471	6.1995	4.8070	70.0511	a-Si
S	1.4972	6.2615	5.1310	68.9286	th-S
F	2.2113	4.5627	3.8610	50.6584	z-F
Br	2.3843	6.8916	5.8625	84.2785	a-Br
I	2.4894	8.2029	7.6777	191.5190	a-I
Cl	2.6095	7.0157	6.0658	108.4480	a-Cl
S-B	0.2479	6.1938	5.1622	74.4984	S-B
S-P	0.5807	6.0762	5.3171	41.9430	S-P
S-N	0.9084	5.4990	4.5129	85.5226	S-N
S-Sb	0.9753	6.7059	5.5692	58.4166	S-Sb
S-O	0.9892	5.6394	4.3366	97.2307	S-O
S-Si	1.2471	7.5487	6.4523	75.6393	S-Si
S-F	2.2113	3.1751	2.6292	50.1810	S-F
S-Br	2.3843	8.5912	7.8241	130.6730	S-Br
S-I	2.4894	-	-	-	-
S-Cl	2.6095	9.6724	9.3778	328.9450	S-Cl

Supplementary Table 9 Summary of calculated density of states (DOSs) including DOS1, DOS2 and DOS3 as defined above of optimal doped-graphene-supported Ca single atoms models.

Dopants	$\emptyset = \frac{E_X A_X R_X / N_X}{E_C A_C R_C / N_C}$	DOS1	DOS2	DOS3	Optimal doped-graphene-supported Ca single atom structures
C	1	5.6698	5.2744	88.1415	z-C-Ca
B	0.2479	6.0114	5.5691	75.8996	B=C-Ca
P	0.5807	5.5392	4.9436	32.4135	P-3C-Ca
N	0.9084	5.2976	4.7920	162.0420	py-N-Ca
Sb	0.9753	6.0837	5.6604	79.8720	py-Sb-Ca
O	0.9892	5.5540	4.9721	91.8413	py-O-Ca
Si	1.2471	4.2544	3.7817	96.2426	a-Si-Ca
S	1.4972	4.6233	3.9177	72.2745	th-S-Ca
F	2.2113	4.0618	3.4906	80.0595	z-F-Ca
Br	2.3843	5.0244	4.6906	58.5805	a-Br-Ca
I	2.4894	4.3074	3.9701	72.9811	a-I-Ca
Cl	2.6095	5.0459	4.7899	56.7659	a-Cl-Ca
S-B	0.2479	4.1954	3.8263	37.9123	S-B-Ca
S-P	0.5807	4.1883	3.8459	89.8705	S-P-Ca
S-N	0.9084	3.6005	3.2098	30.0677	S-N-Ca
S-Sb	0.9753	4.6005	4.0481	83.8409	S-Sb-Ca
S-O	0.9892	4.7736	4.5261	149.3290	S-O-Ca
S-Si	1.2471	5.8536	5.4089	56.5592	S-Si-Ca
S-F	2.2113	3.4477	2.9755	105.4540	S-F-Ca
S-Br	2.3843	6.4568	5.9491	113.9600	S-Br-Ca
S-I	2.4894	-	-	-	-
S-Cl	2.6095	4.7411	4.4453	79.6752	S-Cl-Ca

Supplementary Table 10 Summary of calculated density of states (DOSs) including DOS1, DOS2 and DOS3 as defined above of optimal doped-graphene-supported Ca single atoms with H_2 molecules adsorbed on their optimal active sites.

Dopants	$\emptyset = \frac{E_X A_X R_X / N_X}{E_C A_C R_C / N_C}$	DOS1	DOS2	DOS3	Optimal doped-graphene-supported Ca single atom structures with H_2
C	1	5.1807	4.6770	51.1567	z-C-Ca-H_2
B	0.2479	5.7347	5.4044	71.8251	B=C-Ca-H_2
P	0.5807	5.6977	5.1001	28.2980	P-3C-Ca-H_2
N	0.9084	5.8917	4.7511	108.7990	py-N-Ca-H_2
Sb	0.9753	5.7639	4.8021	50.5066	py-Sb-Ca-H_2
O	0.9892	6.1136	5.4886	93.4630	py-O-Ca-H_2
Si	1.2471	4.4999	4.0013	102.2200	a-Si-Ca-H_2
S	1.4972	5.1612	4.8884	51.8576	th-S-Ca-H_2
F	2.2113	5.1177	4.5584	102.4620	z-F-Ca-H_2
Br	2.3843	4.4520	4.0942	53.8409	a-Br-Ca-H_2
I	2.4894	4.9701	4.3851	133.6880	a-I-Ca-H_2
Cl	2.6095	4.4468	4.1668	74.6184	a-Cl-Ca-H_2
S-B	0.2479	4.1796	3.5808	49.2472	S-B-Ca-H_2
S-P	0.5807	4.7861	4.0307	43.8572	S-P-Ca-H_2
S-N	0.9084	4.7952	4.1247	117.7220	S-N-Ca-H_2
S-Sb	0.9753	4.1277	3.5123	55.1704	S-Sb-Ca-H_2
S-O	0.9892	3.7217	3.3677	41.1686	S-O-Ca-H_2
S-Si	1.2471	6.2488	4.5389	91.7678	S-Si-Ca-H_2
S-F	2.2113	3.4967	3.3166	79.4677	S-F-Ca-H_2
S-Br	2.3843	5.6319	5.2488	77.2373	S-Br-Ca-H_2
S-I	2.4894	-	-	-	-
S-Cl	2.6095	4.3792	3.8848	132.8440	S-Cl-Ca-H_2

4. Supplementary Notes

Note 1

Normalization process from experimental H_2 storage specific capacity C_m (wt%) to specific capacity per unit H_2 storage site $C_{0/site}^*$ (H_2 site^{-1})

First, H_2 molecule storage site can be simply regarded as hollow site of doped-graphene-supported Ca single-atom surface, implying that a H_2 molecule occupies averagely two net carbon atoms on doped-graphene-supported Ca single-atom surface. Based on geometric surface area of a carbon atom on graphene (~2.6×10^{-20} m^2), geometric surface area per H_2 molecule storage site should be 5.2×10^{-20} m^2 due to a hollow site comprising two net carbon atoms. Assuming the host material mass for H_2 storage is 1g, the H_2 mass stored on host material, namely the mass of H_2 stored per unit mass host material (g/g), can be calculated according to

$$C_m(wt\%) = \frac{m_{H_2}}{m_{H_2}+m_{Host}} \times 100\% \tag{83}$$

Here, $C_m(wt\%)$, m_{H_2} and m_{Host} are hydrogen storage specific capacity, the hydrogen molecules mass and host material mass, respectively. Taking nitrogen-doped-graphene-supported Ca single-atom host material (N-G-Ca) as an example, C_m =1.33 wt% (experimental results at 77K and 5MPa) will lead to

$$m_{H_2} = \frac{C_m m_{Host}}{1-C_m} = 0.0135g. \tag{84}$$

Thus, the amount of H_2 molecules stored was calculated to be 4.06×10^{21} according to

$$N=(m/M)N_A \tag{85}$$

Here, N, m, M and N_A are the amount, mass, molar mass, and Avogadro constant (~6.02×10^{23}), respectively. In addition, combining the surface area of N-G-Ca sample (~325 m^2 g^{-1}), the total amount of H_2 molecules storage sites can roughly be estimated to be $325/(5.2 \times 10^{-20})=6.25\times10^{21}$. Consequently, specific capacity per unit H_2 storage site $C_{0/site}^*$ (H_2 site^{-1}) of doped-graphene-supported Ca single-atom surface mainly originating from heteroatom doping and Ca single atom loading can be estimated to be $0.33\%\times (4.06\times10^{21})/(6.25\times10^{21})=2.14\times10^{-3}$. Here, it deserves to note that although doping concentration has an impact on specific capacity of H_2 storage, its role is realized mainly via tuning metal Ca single atoms. Therefore, compared with Ca single atom loading, contribution of heteroatom doping concentration to specific capacity can be roughly ignored.

Conclusively, specific capacity per unit H_2 storage site $C_{0/site}{}^{*}$ (H_2 site^{-1}) of doped-graphene-supported Ca single-atom surface can be given by

$$C_{H_2/site}{}^{*} = \frac{5.2\times10^{-20}\times at\%(Ca)\times\frac{C_m}{1-C_m}\times\frac{N_A}{M}}{S_a} \qquad (86)$$

where $at\%(Ca)$, C_m, N_A, M, and S_a are Ca loading content, hydrogen capacity (wt%), Avogadro constant, molar mass and surface area, respectively.

Note 2

Normalization process from gravimetric capacity C_m (wt%) to area specific capacity C_a (g m^{-2}) for carbon-based adsorption-type H_2 storage host materials

According to the calculation method of gravimetric capacity C_m (wt%) for H_2 storage, namely

$$C_m(wt\%) = \frac{m_{H_2}}{m_{H_2}+m_{Host}}\times 100\% \qquad (87)$$

where $C_m(wt\%)$, m_{H_2} and m_{Host} are hydrogen storage gravimetric capacity, the hydrogen molecules mass and host material mass, respectively, the area specific capacity C_a (g m^{-2}) can be expressed as

$$C_a(g\ m^{-2}) = \frac{C_m}{(1-C_m)S_a} \qquad (88)$$

where S_a is BET surface area of H_2 host materials.

Note 3

Predicted H_2 storage capacity for dual-doped-graphene-supported single-atom Ca NC-SHSMs (S-Si-G-Ca).

In experiment, the hydrogen storage capacity C_m ($wt\%$) of as-prepared N-G-Ca at 77K and 2MPa was tested to be 0.8 wt%, the area specific capacity C_a (g m^{-2}) can be calculated to 2.48 × 10^{-5} g m^{-2} according to the above Equation (88) $C_a(g\ m^{-2}) = \frac{C_m}{(1-C_m)S_a}$ (S_a (~325 m^2 g^{-1}) is BET surface area of N-G-Ca).

In theory, based on the calculated Gibbs free energy change $\Delta G_{H_2^*}^{min}$ (~0.21 eV) before and after H_2 molecule adsorbed on the optimal site of N-G-Ca, for given $\Delta G_{H_2^*}^{max}$ (~0.5 eV), the specific capacity $C_{H_2/site}$, namely the averaged amount of H_2 molecules adsorbed one site can be calculated to be to 2.64 × 10^{-5} H_2/site according to the above Equation (26). Thus, based on the geometric area of one H_2 molecule storage site ($2S_c$=5.2×10^{-20} m^2) and Equation (85) N=(m/M)N_A (N, m, M

and N_A (~6.02×10^{23}) are the amount, mass, molar mass, and Avogadro constant, respectively), the theoretical area specific capacity C_a*(g m^{-2}) was calculated to be 1.69 × 10^{-9} g m^{-2}.

Thus, the correction factor μ was calculated to 1.47×10^4 according to Equation (89).

$$\mu = C_a / C_a^* \quad (89)$$

Here, C_a and C_a* are the experimental area specific capacity (g m^{-2}) and the theoretical area specific capacity (g m^{-2}), respectively.

Under the same physical properties including BET surface area (325m^2 g^{-1}), Ca loading mass (0.33 at%), doping concentration (7.61 at%), and external conditions (77K and 2MPa) as that of as-prepared N-G-Ca NC-SHSMs in our experiment, the correction factor μ should be the same. Therefore, for dual-doped-graphene-supported single-atom Ca (S-Si-G-Ca), the calculated $\Delta G_{H_2^*}^{min}$ (~0.008 eV) can lead to the theoretical specific capacity C_a* =1.776 ×10^{-7} g m^{-2}. Thus, the predicted practical area specific capacity C_a (g m^{-2}) of S-Si-G-Ca under the same conditions and physical properties as that of N-G-Ca was calculated to 2.61 × 10^{-3} g m^{-2}. Finally, the logarithm of area specific capacity

ln (C_a) was obtained to compare with the H_2 storage properties of other host materials including zeolite-like carbon[4], N-doped microporous carbon[6], activated carbon (AC)[7], ultrahigh surface area carbon[8], graphene-like nanosheet[11] and oxygen-rich microporous carbon[20] under the same external conditions (Supplementary Table S1).

5. Supplementary References

1. Gao, Y. et al. Highly pseudocapacitive storage design principles of heteroatom-doped graphene anode in calcium-ion batteries. *Adv. Funct. Mater.***1**, 1-9 (2023).

2. Jiao, Y., Zheng, Y., Kenneth, D. & Qiao, S. Z. Activity origin and catalyst design principles, for electrocatalytic hydrogen evolution on heteroatom-doped graphene. *Nat. Energy* **1**, 1-9 (2016).

3. Liu, Y. Y., He, G. J., Jiang, H., Parkin, I. P., Shearing, P. R. & Brett, D. J. L., Cathode design for aqueous rechargeable multivalent ion batteries: challenges and opportunities. *Adv. Funct. Mater.* **3**, 1-9 (2021).

4. Yang, Z., Xia, Y. & Mokaya, R. Enhanced hydrogen storage capacity of high surface area zeolite-like carbon materials. *J. Am. Chem. Soc.* **129**, 1673-1679 (2007).

5. Kabbour, H., Baumann, T. F., Satcher, J. H. & Saulnier, A. Toward new candidates for hydrogen storage: high-surface area carbon aerogels. *Chem. Mater.* **18**, 6085-6092 (2006**)**.

6. Xia, Y. D., Mokaya, R., Grant, D. M. & Walker, G. S. A simplified synthesis of N-doped zeolite-templated carbons, the control of the level of zeolite-like ordering and its effect on hydrogen storage properties. *Carbon.* **49**, 844-853 (2011).

7. Wang, H., Gao, Q. & Hu, J. High hydrogen storage capacity of porous carbons prepared by using activated carbon. *J. Am. Chem. Soc.***131**, 7016-7022 (2011).

8. Sevilla, M., Mokaya, R. & Fuertes, A. B. Ultrahigh surface area polypyrrole-based carbons with superior performance for hydrogen storage. *Energy Environ. Sci.* **4**, 2930-2938 (2011).

9. Wang, Y. et al. Reversible hydrogen storage of multi-wall carbon nanotubes doped with atomically dispersed lithium. *J. Mater. Chem.* **20**, 6490-6494 (2010).

10. Reyhani, A. et al. Hydrogen storage in decorated multiwalled carbon nanotubes by Ca Co, Fe, Ni, and Pd nanoparticles under ambient conditions. *J. Phys. Chem. C* **115**, 6994-7001(2011).

11. Srinivas, G. et al. Synthesis of graphene- like nanosheets and their hydrogen adsorption capacity. *Carbon* **48**, 630-635 (2010).

12. Guo, C. X., Wang, Y. & Li, C. M. Hierarchical graphene-based material for Over 4.0 wt% physisorption hydrogen storage capacity. *ACS Sustain. Chem. Eng.* **1**,14-18(2013).

13. Aboutalebi, S. H. et al. Enhanced hydrogen storage in graphene oxide- MWCNTs composite at room temperature. *Adv. Energy Mater.* **2**,1439-1446(2012).

14. Huang, C. C. et al. Hydrogen storage in graphene decorated with Pd and Pt nano-particles using an electroless deposition technique. *Sep. Purif. Technol.* **82**, 210-215(2011).

15. Parambhath, V. B., Nagar, R. & Ramaprabhu, S. Effect of nitrogen doping on hydrogen storage capacity of palladium decorated graphene. *Langmuir* **28**,7826-7833(2012).
16. Wang, Y. et al. Hydrogen storage in a Ni-B nanoalloy-doped three-dimensional graphene material. *Energy Environ. Sci.* **4**, 195-200 (2011).
17. Cho, E. S. et al. Graphene oxide/metal nanocrystal multilaminates as the atomic limit for safe and selective hydrogen storage. *Nat. Commun.* **7**, 10804-10812 (2016).
18. Li, A. et al. Lithium-Doped Conjugated Microporous Polymers for Reversible Hydrogen Storage. *Angew. Chem. Int. Ed.***122**, 3402-3405(2010).
19. Liu, S. Y. et al. Hydrogen storage in incompletely etched multilayer Ti_2CT_x at room temperature. *Nat. Nanotechnol.* **16**, 331-336 (2021).
20. Blankenship, T. S., Balahmar, N. & Mokaya, R. Oxygen-rich microporous carbons with exceptional hydrogen storage capacity. *Nat. Commun.* **8**,1545-1552 (2017).
21. Zhou, C. Y. & Szpunar, J. A. Hydrogen Storage Performance in Pd/Graphene Nanocomposites. *ACS Appl. Mater. Interfaces* **8**, 25933-25940 (2016).
22. Zhou, C. Y., Szpunar, J. A. & Cui, X. Y. Synthesis of Ni/Graphene Nanocomposite for Hydrogen Storage. *ACS Appl. Mater. Interfaces* **8**, 15232-15241(2016).
23. Zhou, H. et al. Enhanced room-temperature hydrogen storage capacity in Pt-loaded graphene oxide/HKUST-1 composites, *Int. J. Hydrogen Energy* **39**, 2160-2167(2014).
24. Xia, G. L. et al. Graphene-wrapped reversible reaction for advanced hydrogen storage, *Nano Energy* **26**, 488-495(2016).
25. Sarkar, A. K. et al. Hydrogen storage on graphene using Benkeser reaction. *Int. J. Energy Res.* **38**, 1889-1895(2014).
26. Ariharan, A., Viswanathan, B. & Nandhakumar, V. Heteroatom doped multi-layered graphene material for hydrogen storage application. *Graphene* **5**, 39-50 (2016).
27. Ariharan, A., Viswanathan, B. & Nandhakumar, V. Nitrogen doped graphene as potential material for hydrogen storage, *Graphene* **6**, 41-60 (2017).
28. Tarasov, B. P. et al. Hydrogen storage behavior of magnesium catalyzed by nickel-graphene nanocomposites, *Int. J. Hydrogen Energy* **44**, 29212-29223 (2019).
29. Xia, G. L. et al. Oxygen-free layer-by-layer assembly of lithiated composites on graphene for advanced hydrogen storage. *Adv. Sci.* **4**, 1-10 (2017).

30. Suttisawat, P. Y. et al. Investigation of hydrogen storage capacity of multi-walled carbon nanotubes deposited with Pd or V. *Int. J. Hydrogen Energy* **34**, 6669-6675(2009).
31. Muthu, R. N., Rajashabala, S. & Kannan, R. Hexagonal boron nitride (h-BN) nanoparticles decorated multi-walled carbon nanotubes (MWCNT) for hydrogen storage. *Renew. Energy* **85**, 387-394 (2016).
32. Huang, Y. K. et al. Layer-by-layer uniformly confined graphene-$NaAlH_4$ composites and hydrogen storage performance. *Int. J. Hydrogen Energy* **45**, 28116-28122 (2020).
33. Cristina, R. G. et al. Clay-supported graphene materials: application to hydrogen storage. *Phys. Chem. Chem. Phys.* **15**, 18635-18641(2013).
34. Ariharan, A., Viswanathan, B. & Nandhakumar, V. Phosphorous-doped porous carbon derived from paste of newly growing Ficus benghalensis as hydrogen storage material. *Indian J. Chem.* **55**, 649-656 (2016).
35. Zeng, L. A. et al. Enhanced hydrogen storage properties of MgH_2 Using a Ni and TiO_2 co-doped reduced graphene oxide nanocomposite as a catalyst. *Front. Chem.* **8**, 1-8 (2020).
36. Klechikov, A. et al. Hydrogen storage in high surface area graphene scaffolds. *Chem. Commun.* **51**, 15280-15283(2015).
37. Rajaura, R. S. et al. Role of interlayer spacing and functional group on the hydrogen storage properties of graphene oxide and reduced graphene oxide. *Int. J. Hydrogen Energy* **41**, 9454-9461(2016).
38. Kim, B. H. et al. Thermally modulated multilayered graphene oxide for hydrogen storage. *Phys. Chem. Chem. Phys.* **14**, 1480-1484(2012).
39. Lan, Z. Q. et al. Synthetical catalysis of nickel and graphene on enhanced hydrogen storage properties of magnesium. *Int. J. Hydrogen Energy* **44**, 24849-24855(2019).
40. Ngqalakwezi, A., Nkazi, D., Seifert, G. & Ntho, T. Effects of reduction of graphene oxide on the hydrogen storage capacities of metal graphene nanocomposite. *Catal. Today* **358**, 338-344 (2020).
41. Gu, J., Zhang, X. P., Fu, L. & Pang, A. M. Study on the hydrogen storage properties of the dual active metals Ni and Al doped graphene composites. *Int. J. Hydrogen Energy* **44**, 6036-6044 (2019).
42. Chen, P., Wu, X., Lin, J. & Tan, K. L. High H_2 Uptake by alkali-doped carbon nanotubes under ambient pressure and moderate temperatures. *Science* **285**, 91-93 (1999).

43. Xia, G. L. et al. Monodisperse magnesium hydride nanoparticles uniformly self-assembled on graphene. *Adv. Mater.* **27**, 5981-5988(2015).
44. Teprovich, J. A. et al. Comparative study of reversible hydrogen storage in alkali-doped fulleranes. *J. Alloys Compd.* **580**, 364-367(2013).
45. Mashoff, T. et al. Hydrogen storage with titanium-functionalized graphene, *Appl. Phys. Lett.* **103**, 013903-013907(2013).
46. Broom, D. P. et al. Concepts for improving hydrogen storage in nanoporous materials. *Int. J. Hydrogen Energy* **44**, 7768-7779 (2019).
47. Klechikov, A. G. et al. Hydrogen storage in bulk graphene-related materials. *Micropor. Mesopor. Mat.* **210**, 46-51(2015).
48. Wan, L. F. et al. Edge-functionalized graphene nanoribbon encapsulation to Enhance stability and control kinetics of hydrogen storage materials. *Chem. Mater.* **31**, 2960-2970(2019).
49. Ruse, E. et al. Hydrogen storage kinetics: the graphene nanoplatelet size effect. *Carbon* **130**, 369-376(2018).
50. Yoon, H. et al. Reversible phase modulation and hydrogen storage in multivalent VO_2 epitaxial thin films. *Nat. Mater.* **15**,1113-1119(2015).
51. Nachimuthu, S., Lai, P. J. & Jiang, J. C. Efficient hydrogen storage in boron doped graphene decorated by transition metals-A first-principles study. *Carbon* **73**, 132-140(2014).
52. Ao, Z. M., Jiang, Q., Zhang, R. Q., Tan, T. T. & Li, S. Al doped graphene: A promising material for hydrogen storage at room temperature. *J. Appl. Phys.***105**, 074307-074315(2009).
53. Ao, Z. M. & Peeters, F. M. High-capacity hydrogen storage in Al-adsorbed graphene. *Phys. Rev. B*, **81**, 205406-205412(2010).
54. Lee, H. et al. Calcium-decorated graphene-based nanostructures for hydrogen storage. *Nano Lett.***10**, 793-798(2010).
55. Lee, H. et al. Calcium-decorated carbon nanotubes for high-capacity hydrogen storage: First-principles calculations. *Phys. Rev. B* **80**, 115412-115416(2009).
56. Kim, Y. H. et al. Nondissociative Adsorption of H_2 Molecules in light-element-doped fullerenes. *Phys. Rev. Lett.* **96**, 016102-016106(2006).
57. Du, A. J., Zhu, Z. H. & Smith, S. C. Multifunctional porous graphene for nanoelectronics and hydrogen storage: new properties revealed by first principle calculations. *J. Am. Chem. Soc.* **132**, 2876-2877(2010).

58. Sathishkumar, N., Wu, S. Y. & Chen, H. T. Charge-modulated/electric-field controlled reversible CO_2/H_2 capture and storage on metal-free N-doped penta-graphene. *Chem. Eng. J.* **391**, 123577-123585(2020).
59. Zhou, W. W. et al. First-principles study of high-capacity hydrogen storage on graphene with Li atoms. *J. Phys. Chem. Solids* **73**, 245-251(2012).
60. Dou, S. X. et al. Hydrogen storage in porous graphene with Al Decoration. *Int. J. Hydrogen Energy*, **3**, 1-8(2014).
61. Dimitrakakis, G. K., Tylianakis, E. & Froudakis, G. E. Pillared graphene: a new 3-D network nanostructure for enhanced hydrogen storage. *Nano Lett*. **8**, 3166-3170(2008).
62. Reunchan, P. & Jhi, S. H. Metal-dispersed porous graphene for hydrogen storage. *Appl. Phys. Lett.* **98**, 093103-093109(2011).
63. Furuta, T., Goto, H., Ohashi, T. & Fujiwara, Y. Theoretical evaluation of hydrogen storage capacity in pure carbon nanostructures. *J. Chem. Phys.* **114**, 2376-2385(2003).
64. Wang, L. et al. Graphene oxide as an ideal substrate for hydrogen storage. *ACS Nano* **3**, 2995-3000 (2009).
65. Ataca, C., Aktürk, E. & Ciraci, S. Hydrogen storage of calcium atoms adsorbed on graphene: first-principles plane wave calculations, *Phys. Rev. B* **79**,1-4 (2009).
66. Zhao, Y. F. et al. Hydrogen storage in novel organometallic buckyballs. *Phys. Rev. Lett.* **94**, 155504-155511(2005).
67. Faye, O. et al. Hydrogen adsorption and storage on Palladium-functionalized graphene with NH-dopant: A first principles calculation. *Appl. Surf. Sci.* **392**, 362-374 (2017).
68. Chen, I. N., Wu, S. Y. & Chen, H. T. Hydrogen storage in N- and B-doped graphene decorated by small platinum clusters: A computational study. *Appl. Surf. Sci.* **441**, 607-612(2018).
69. Sigal, A., Rojas, M. I. & Leiva, E. P. Interferents for hydrogen storage on a graphene sheet decorated with nickel: A DFT study. *Int. J. Hydrogen Energy* **36**, 3537-3546(2011).
70. Choudhary, A. et al. First principles calculations of hydrogen storage on Cu and Pd-decorated graphene. *Int. J. Hydrogen Energy* **41**, 17652-17656 (2016).
71. Luo, Z. F., Fan, X. L., Pan, R. & An, Y. R. A first-principles study of Sc-decorated graphene with pyridinic-N defects for hydrogen storage. *Int. J. Hydrogen Energy* **42**, 3106-3113(2017).
72. Lee, H., Choi, W. I. & Ihm, J. Combinatorial search for optimal hydrogen-storage nanomaterials based on polymers. *Phys. Rev. Lett.* **97**, 056104-056110(2006).

73. Ma, L., Zhang, J. M., Xu, K. W. & Ji, V. Hydrogen adsorption and storage on palladium-decorated graphene with boron dopants and vacancy defects: A first-principles study. *Phys. E* **66**, 40-47(2015).
74. Amaniseyed, Z. & Tavangar, Z. Hydrogen storage on uncharged and positively charged Mg-decorated graphene. *Int. J. Hydrogen Energy* **44**, 3803-3811(2019).
75. Sathishkumar, N. & Wu, S. Y. Boron- and nitrogen-doped penta-graphene as a promising material for hydrogen storage: A computational study. *Int. J. Energy Res.* **43**, 4867-4878(2019).
76. Zheng, N. et al. A DFT study of the enhanced hydrogen storage performance of the Li-decorated graphene nanoribbons. *Vacuum* **171**,109011-109018(2020).
77. Zhang, X., Tang, C. M. & Jiang, Q. G. Electric field induced enhancement of hydrogen storage capacity for Li atom decorated graphene with Stone-Wales defects. *Int. J. Hydrogen Energy.* **2**, 1-10 (2016).
78. Yuan, L. H. et al. First-principles study of V-decorated porous graphene for hydrogen storage, *Chem. Phys. Lett.* **726**, 57-61(2019).
79. Yadav, S., Zhu, Z. H. & Singh, C. V. Defect engineering of graphene for effective hydrogen storage. *Int. J. Hydrogen Energy* **39**, 4981-4995(2014).
80. Wang, V. et al. Calcium-decorated graphene for hydrogen storage: A van der Waals density functional study. *Comp. Mater. Sci.* **55**, 180-185(2012).
81. Mananghaya, M., Yu, D., Santos, G. N. & Rodulfo, E. Scandium and titanium containing single-walled carbon nanotubes for hydrogen Storage: a thermodynamic and first principle calculation. *Sci. Rep.* **6**, 27370-27376 (2016).
82. Beheshti, E., Nojeh, A. & Servati, P. A first-principles study of calcium-decorated, boron-doped graphene for high capacity hydrogen storage. *Carbon* **49**, 1561-1567(2011).
83. Wu, H. Y., Fan, X. F., Kuo, J. L. & Deng, W. Q. DFT study of hydrogen storage by spillover on graphene with boron substitution. *J. Phys. Chem. C* **115**, 9241-9249(2011).
84. Sunnardianto, G. K. et al. Efficient hydrogen storage in defective graphene and its mechanical stability: A combined density functional theory and molecular dynamics simulation study. *Int. J. Hydrogen Energy* **46**, 5485-5494(2021).
85. He, Y. L. & Wang, S. X. Hydrogen storage of platinum atoms adsorbed on graphene: first-principles plane wave calculations. *Appl. Mech. Mater.* **2**, 1057-1060(2013).

86. Ataca, E. C., Aktürk, S. C. & Ustunel, H. High-capacity hydrogen storage by metallized graphene. *Appl. Phys. Lett.* **93**, 043123-043130(2008).
87. Aydin, S. & Simsek, M. The enhancement of hydrogen storage capacity in Li, Na and Mg-decorated BC_3 graphene by CLICH and RICH algorithms. *Int. J. Hydrogen Energy* **44**, 7354-7370 (2019).
88. Bhattacharya, A., Bhattacharya, S., Majumder, C. & Das, G. P. Transition-metal decoration enhanced room-temperature hydrogen storage in a defect-modulated graphene sheet. *J. Phys. Chem. C* **114**, 10297-10301(2010).
89. Cho, J. H., Yang, S. J. & Park, C. R. Si-doping effect on the enhanced hydrogen storage of single walled carbon nanotubes and graphene. *Int. J. Hydrogen Energy* **36**, 12286-12295(2011).
90. Chu, S. B. et al. Titanium-embedded graphene as high-capacity hydrogen-storage media. *Int. J. Hydrogen Energy* **36**, 12324-12328(2011).
91. Faye, O. et al. Hydrogen storage on bare Cu atom and Cu-functionalized boron-doped graphene: A first principles study. *Int. J. Hydrogen Energy* **42**, 4233-4243(2017).
92. Ganji, M. D., Emami, S.N., Khosravi, A., & Abbasi, M. Si-decorated graphene: A promising media for molecular hydrogen storage. *Appl. Surf. Sci.* **332**, 105-111(2015).
93. Gao, Y. et al. Hydrogen spillover storage on Ca-decorated graphene. *Int. J. Hydrogen Energy* **37**,11835-11841(2012).
94. Tan, X., Tahini, H. A. & Smith, S. C. Conductive boron-doped graphene as an ideal material for electrocatalytically switchable and high-capacity hydrogen storage. *ACS Appl. Mater. Interfaces* **8**, 32815-32822(2016).
95. Tachikawa, H. & Iyama, T. Mechanism of hydrogen storage in the graphene nanoflake-lithium-H_2 system. *J. Phys. Chem. C* **123**, 8709-8716 (2019).
96. Liu. C. S. & Zeng, Z. Boron-tuned bonding mechanism of Li-graphene complex for reversible hydrogen storage. *Appl. Phys. Lett.* **96**, 123101-123108(2010).
97. Lin, Y., Ding, F. & Yakobson, B. I. Hydrogen storage by spillover on graphene as a phase nucleation process. Phys. Rev. B 78, 041402-041409 (2008).
98. Li, Y. et al. The structural and electronic properties of Li-doped fluorinated graphene and its application to hydrogen storage. *Int. J. Hydrogen Energy* **37**, 5754-5761(2012).
99. Cabria, I., López M. J. & Alonso, J. A. Enhancement of hydrogen physisorption on graphene and carbon nanotubes by Li doping. *J. Chem. Phys.* **123**, 204721-204728(2005).

100. Sabir, A. K., Lu, W.C., Roland, C. & Bernholc, J. Ab initio simulations of H_2 in Li-doped carbon nanotube systems. *J. Phys-Condens Mater.* **19**, 086226-086232(2007).
101. Kaiser, A. et al. On enhanced hydrogen adsorption on alkali (cesium) doped C_{60} and effects of the quantum nature of the H_2 molecule on physisorption energies. *Int. J. Hydrogen Energy* **42**, 3078-3086(2017).
102. Ebadi, M., et al. Calcium-decorated graphdiyne as a high hydrogen storage medium: Evaluation of the structural and electronic properties. *Int. J. Hydrogen Energy* **43**, 23346-23356 (2018).
103. Froudakis, G. E. et al. Why alkali-metal-doped carbon nanotubes possess high hydrogen uptake. *Nano Lett.* **1**, 531-533(2001).
104. Tozzini, V. & Pellegrini, V. Reversible hydrogen storage by controlled buckling of graphene layers. *J. Phys. Chem. C.* **115**, 25523-25528(2011).
105. Tan, X. et al. Charge modulation in graphitic carbon nitride as a switchable approach to high-capacity hydrogen storage. *ChemSusChem* **8**, 3626-3631(2015).
106. Li, J. et al. Theoretical evaluation of hydrogen storage capacity in pure carbon nanostructures. *J. Chem. Phys.* **119**, 2376-2385(2003).
